# Dietary Interventions with *Bletilla striata* Polysaccharides and/or Composite Polysaccharides Remodel Liver Lipid Profiles and Ameliorate Gut Metabolic Disturbances in High-Fat Diet-Induced Obese Mice

**DOI:** 10.3390/foods14152653

**Published:** 2025-07-29

**Authors:** Peiting Zhang, Jinjin Dong, Jiamin Lu, Zijian Cai, Bingde Zhou, Qian Zhang, Chenglin Zhu, Luca Laghi

**Affiliations:** 1 College of Pharmacy and Food, Southwest Minzu University, Chengdu 610041, China; zpeiting@outlook.com (P.Z.); dongjinjin1224@outlook.com (J.D.); lujiamin1979@outlook.com (J.L.); zhoubingde123@outlook.com (B.Z.); zhangqian1121@outlook.com (Q.Z.); chenglin.zhu@swun.edu.cn (C.Z.); 2 Department of Agricultural and Food Sciences, University of Bologna, 47521 Cesena, Italy; l.laghi@unibo.it

**Keywords:** *Bletilla striata* polysaccharide, composite polysaccharide, high-fat diet, gut–liver axis, multi-omics

## Abstract

The global obesity epidemic and associated metabolic disorders present urgent public health challenges. This study employed a multi-omics approach (lipidomics, metabolomics, and gut microbiome analysis) to investigate how *Bletilla striata* polysaccharides (BSPs) and composite polysaccharides modulate liver lipid metabolism and gut microbiota in high-fat diet (HFD)-induced obese mice. HFD elevated hepatic phosphatidylcholines, cholesteryl esters (CEs), and acylcarnitines (CARs), alongside increased cecal choline and trimethylamine. BSP interventions reduced hepatic CEs, free fatty acids (FAs), CARs, and cecal sarcosine while restoring gut microbial diversity. Notably, BSP enriched beneficial genera, including *Jeotgalicoccus* and *Atopostipes*, and the network analysis revealed negative correlations between these genera and hepatic triglycerides (TGs), implicating the gut–liver axis in lipid metabolism regulation. These findings elucidate the anti-obesity mechanisms of polysaccharides through gut microbiota remodeling and cross-tissue metabolic interactions, providing a foundation for leveraging plant polysaccharides in developing safer, effective obesity therapies.

## 1. Introduction

The rising global prevalence of obesity and related metabolic disorders presents a significant challenge to public health worldwide, with high-fat diet (HFD) consumption identified as a primary factor [1]. HFD-induced metabolic dysregulation is characterized by disrupted energy homeostasis and alterations in gut microbiome, which collectively establish a pathological foundation for chronic diseases [2]. Various plant-derived polysaccharides, such as those from alfalfa and *Artemisia argyi*, could alleviate metabolic disorders by modulating the gut microbiota, thereby helping reduce obesity [3,4]. Arabinoxylan (AX) and xyloglucan (GX) are novel prebiotics that play an important role in the prevention and treatment of overweight or obesity [5,6]. Moreover, in our team’s previous study, composite polysaccharides (AX + GX) were found to have beneficial anti-obesity properties. In addition, traditional Chinese medicine offers promising therapeutic avenues. *Bletilla striata*, a well-known herbal remedy, is rich in bioactive compounds and has attracted considerable attention for its potential health benefits [7]. *Bletilla striata* polysaccharides (BSPs) are a significant constituent of this traditional medicine, and numerous studies have demonstrated their multifaceted bioactivities, including anti-inflammatory, antioxidant, immunomodulatory, and anti-aging effects [8,9,10]. Notably, BSPs have been shown in vitro to stimulate short-chain fatty acid production and to regulate gut microbiota [11]. Furthermore, *Bletilla striata* oligosaccharides have been found to prevent weight gain in HFD-fed mice and to alleviate metabolic syndrome by modulating gut microbiota and intestinal metabolites [12]. Our previous in vivo study found that supplementation with BSP and/or complex polysaccharides was effective in ameliorating HFD-induced obesity without affecting appetite and also significantly ameliorated disturbances in fasting blood glucose (FBG), total cholesterol (TC), total glyceride (TG), high-density lipoprotein cholesterol (HDL-C), and low-density lipoprotein cholesterol (LDL-C). In addition, BSP supplementation alters the metabolisms of purine, amino acid, ascorbate, aldarate, and pyrimidine; reduces the Firmicutes/Bacteroidetes; and improves the balance of the gut microbiome [13]. However, little research has been carried out to investigate the potential anti-obesity modulatory effects of BSP via the “gut–liver axis”.

The liver plays a central role in lipid–glucose crosstalk, making it particularly susceptible to metabolic disruptions induced by an HFD [14]. HFD consumption leads to liver lipid dysregulation through three primary mechanisms: (1) imbalanced TG and cholesteryl ester (CE) metabolism, resulting in steatosis [15]; (2) lipotoxicity-induced hepatocyte damage, which stimulates the body to release cytokines with pro-inflammatory effects (such as TNF-*α* and IL-6) and activates Kupffer cells, thus exacerbating non-alcoholic fatty liver disease (NAFLD) [16]; and (3) ceramide-mediated impairment of insulin signaling (via the PI3K/Akt pathway), which increases glucose dysregulation [17]. These liver disturbances propagate systemic metabolic consequences, including alterations in the atherogenic lipoprotein profile (elevated VLDL/LDL and reduced HDL) and vascular cholesterol deposition [18]. Specifically, disturbances in liver lipid metabolism have been identified as key contributors to obesity and associated metabolic diseases, such as type 2 diabetes, as well as cerebrovascular diseases like insulin resistance [19,20,21]. Moreover, emerging evidence suggests that liver lipid dysregulation plays a critical role in “gut–liver” axis dysfunction through alterations in bile acid metabolism and subsequent microbiome imbalances [22]. This creates a vicious cycle that exacerbates metabolic disturbances. However, the relationship between liver lipid metabolism and the gut microbiome in the anti-obesity effects of BSP remains unclear, and further investigation into the regulatory mechanisms of the “gut–liver axis” is warranted.

The present study aims to address this gap by comprehensively investigating the role of BSPs and/or composite polysaccharides in HFD-induced lipid metabolism disorders and the regulation of the “gut–liver axis” using an integrated approach that combines lipidomics, metabolomics, and microbiome analysis. This study seeks to harness the therapeutic potential of traditional Chinese medicine to prevent and alleviate obesity and associated metabolic disorders, thereby providing a theoretical foundation for accessing plant polysaccharide resources and exploring safer, more effective anti-obesity approaches.

## 2. Materials and Methods

### 2.1. Materials and Animals

*Bletilla striata* was purchased from the Yunnan local market, China, and authenticated by Prof. Yuan Liu (College of Grassland Resources, Southwest Minzu University). A voucher specimen was deposited at Southwest Minzu University with specimen number SWUCM-BS-2023-001. Arabinoxylan (AX) and xyloglucan (XG) were purchased from Shanghai Ryan Biotechnology Co., Ltd. (Shanghai, China). A total of twenty-five KM mice (six weeks old, male, specific pathogen-free) were obtained from Chengdu Dashuo Biotechnology Co., Ltd. (Chengdu, China). The license number is SCXK (Chuan) 2020-030.

### 2.2. BSP Preparation

BSP samples were prepared following the protocol by Liao et al. [23]. Dried *Bletilla striata* was pulverized and sieved through a 30-mesh screen after being dried at 45 °C. The resulting powder was soaked in 95% ethanol (1:5 *m*/*v* ratio, Sinopharm chemical Reagent co., Ltd., Shanghai, China) for 30 min and then extracted with ethanol (5 mL/g, 95%, Sinopharm chemical Reagent co., Ltd., Shanghai, China) and petroleum ether (5 mL/g, 60–90 °C, Sinopharm chemical Reagent co., Ltd., Shanghai, China) at 70 °C for 2 h. This extraction process was repeated twice. To obtain an aqueous extract, the samples were filtered, dried, and diluted with pure water (1:40 *m*/*v*). The so-obtained mix was stirred for 2 h at 70 °C. The extract was concentrated, and proteins were removed using the Sevag reagent. The concentrated solution was then treated with anhydrous ethanol to achieve an 80% (*v*/*v*) concentration and stored for 12 h at 4 °C. Ethanol, acetone (Sinopharm chemical Reagent co., Ltd., Shanghai, China), and ether (Sinopharm chemical Reagent co., Ltd., Shanghai, China) were used to wash the precipitate before drying at 30 °C. Dialysis with a DEAE-52 cellulose column (Beijing Solarbio Science & Technology Co., Ltd., Beijing, China) was used to purify the crude BSP. The chemical structure of BSP consists primarily of mannose and glucose, with a molar ratio of 2.946:1. Its main chain is predominantly composed of (1→4)-linked *β*-D-mannopyranose. In addition, BSP is a branched polysaccharide comprising a variety of glycosidic bond linkages, such as (1→2), (1→3), and (1→4) linked sugar units. In terms of physicochemical properties, the molecular weight (Mw) of BSP was determined to be 3.73 × 10^–5^ g/mol, and the mean number of molecules (Mn) was calculated to be 6.75 × 10^–4^ g/mol. The total carbohydrate content of BSP was found to be 63.46%. Furthermore, BSP did not show absorption peaks for proteins and nucleic acids in the UV spectrum, indicating that it did not contain these components. For further information regarding BSP, please refer to Liao’s article [23].

### 2.3. Experimental Design

**Figure 1:**
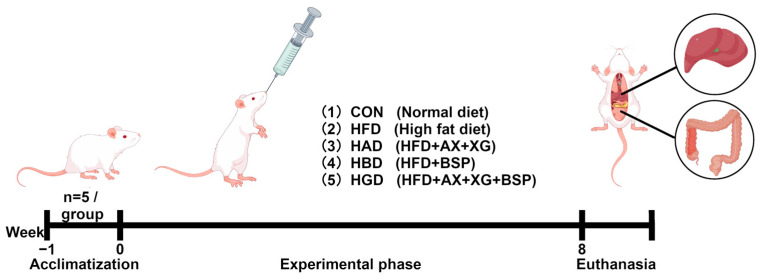
A scheme of animal experimental design.

All mice were maintained under controlled conditions at 24 ± 2 °C and 50 ± 10% relative humidity, and they underwent a cycle of 12 h of light and 12 h of darkness. Prior to the experiment, the mice were acclimatized for one week (week 0) while being fed a normal diet. Subsequently, the mice were assigned randomly to 5 groups: the normal-diet group (CON), the high-fat diet group (HFD), the HFD supplemented with BSP (300 mg/kg/day) (HBD) group, the HFD supplemented with AX (150 mg/kg/day) + XG (150 mg/kg/day) (HAD) group, and the HFD supplemented with AX (100 mg/kg/day) + BSP (100 mg/kg/day) + XG (100 mg/kg/day) (HGD) group. The above doses were in accordance with our previous study [13]. The normal diet provided 10% of energy from fat, while the HFD contained 60% fat energy. Food and water were provided ad libitum throughout the experiment. In the eighth week of experimentation, following a 12 h fast, the mice were rendered unconscious with ether and subsequently euthanized by cervical dislocation [24]. Samples of liver and cecum contents were collected immediately and then stored at −80 °C for subsequent analysis, as depicted in Figure 1. All experimental designs and protocols were in accordance with the guidelines of the Academy for Animal Research and were approved by the Southwest Minzu University Committee for Animal Ethics (Chengdu, China; approval number: SWUN-A-0060).

### 2.4. Lipidomic Analysis

Liver samples of mice were freeze-dried and then ground. A sample of 10 mg was selected, and 500 µL of dichloromethane–methanol (50/50, *v*/*v*, Chengdu Jinshan Chemical Reagent Co., Ltd., Sichuan, China) was added, along with 20 µL of a 1/10 dilution of a Lip Mtx lipid internal standard. The samples were then vortex-mixed for 1 min. Extraction was performed at −20 °C for 20 min, followed by ultrasonic treatment for 5 min. Then, the samples were centrifuged for 5 min at 13,000 rpm and 4 °C. Next, 300 µL of the supernatant was collected and evaporated with a stream of nitrogen. The residue was dissolved again in dichloromethane–methanol (9:1, *v*/*v*), vortex-mixed for 1 min, and centrifuged again for 5 min (4 °C, 13,000 rpm). A 70 µL aliquot of the supernatant was collected for analysis.

The supernatant was subjected to chromatographic separation using an AQ C18 column (100 × 2.1 mm, 1.8 µm, Thermo, Waltham, MA, USA) and subsequently introduced into the mass spectrometry detection system. The mobile phase consisted of two components: phase A was acetonitrile–water (3:2) with formic acid (0.1%, Sinopharm chemical Reagent co., Ltd., Shanghai, China) and amyl acetate (10 mM, Sinopharm chemical Reagent co., Ltd., Shanghai, China), while phase B was constituted by isopropanol–acetonitrile (9:1, Sinopharm chemical Reagent co., Ltd., Shanghai, China) with the same additives. The flow rate of the injection was set to 0.3 mL/min, and the temperature of the column was kept at 35 °C. The optimized gradient elution procedure is detailed in Table 1.

Mass Spectrometry Conditions: Mass spectrometry was conducted in both negative and positive ion modes. The mass scan range was set from 150.0 to 2250.0 *m*/*z*, with data acquisition conducted in full mass/dd-MS2 mode. The resolution was set to 70,000 for full scans and 17,500 for MS/MS. The flow rate of both auxiliary and sheath gasses was set at 40 Arb, while the capillary temperature was maintained at 320 °C and 300 °C for the positive and negative ion modes, respectively. The voltage of spray was 3.3 kV and 2.8 kV for the positive and the negative ion modes, respectively, with a nebulized gas temperature of 350 °C. Data acquisition was carried out over a period of 20 min.

### 2.5. Metabolomic Analysis

Cecum content samples for ^1^H-NMR analysis were prepared following a previously described method [25]. In brief, 1 mL of deionized water was added to 80 mg of each cecum content and mixed by vortex for 5 min. To remove solid residues, centrifugation was performed at 18,630× *g* and 4 °C for 15 min. The supernatant (0.7 mL) was collected and mixed with 0.2 mL of the NMR analysis solution, followed by centrifugation under the same conditions.

Using a 600.13 MHz AVANCE III spectrometer (Bruker, Wuhan, China), ^1^H-NMR spectroscopy was performed at 298 K. Each spectrum was phase-corrected using version 4.2 of Topspin software. The subsequent processing of spectra and the quantification of molecules were performed by relying on R scripts customized for our purposes. The baseline of each spectrum was adjusted by removing the signal of residual water, followed by the application of the algorithm “rolling ball” from the R (v.4.4.2) baseline package based on the detection of peaks. For molecular identification, the chemical shifts and multiplicities of signals were compared with the spectra of standard pure compounds available in the Chenomx library (Chenomx Inc., Edmonton, AB, Canada, version 8.4).

Proteins and water variations among the samples were accounted for by probabilistic quotient normalization (PQN). TSP (3-(trimethylsilyl)-propionic-2,2,3,3-d4) acid sodium salt was used as an internal standard for molecule quantification. Rectangular integration was applied to calculate the area under each signal for quantification.

### 2.6. Microbiome Analysis

The extraction of total DNA from cecum contents was performed using the E.Z.N.A.^®^ Soil DNA Kit (Omega Bio-tek, Norcross, GA, USA). The region V3-V4 of the 16S rRNA gene was amplified with PCR, using the primer pairs 338F (5′-ACTCCTACGGGGAGGCAGCAG-3′) and 806R (5′-GGACTACHVGGTWTCTAAT-3′). Gel electrophoresis, purified using the AxyPrep DNA Gel Extraction Kit (Shanghai Majorbio Bio-Pharm Technology Co.,Ltd., Shanghai, China), was used to verify the products of PCR, which were then quantified using the QuantiFluor™-ST fluorometer (Promega, Madison, WI, USA). Subsequently, the samples were sequenced using the Illumina platform (Illumina, San Diego, CA, USA). QIIME2 (version 2022.2) was used to process and analyze raw sequencing data and to acquire amplicon sequence variants (ASVs) for further investigation.

### 2.7. Statistical Analysis

R language was employed for statistical analyses. Before any univariate analysis, non-normally distributed data were brought to normality through the Box-Cox method [26]. Data are presented as mean ± standard deviation (SD). One-way ANOVA followed by Tukey’s Honest Significant Difference (HSD) test (*p* < 0.05) was used to evidence significant differences in molecules and microbiota among the investigated groups. Independent t-tests were used to compare the CON and HFD groups by accepting a significance level of *p* < 0.05.

Robust principal component analysis (rPCA) models were constructed to visualize trends in the metabolomic and microbiome data. For each rPCA model, a score plot and Pearson correlation plot were generated based on the loadings. MetaboAnalyst 5.0 (www.metaboanalyst.ca, accessed on 2 January 2025) was used to conduct partial least squares discriminant analysis (PLS-DA). The free online platform for data analysis, Metware Cloud (https://cloud.metware.cn, accessed on 12 Febrary 2025), was then used for correlation network analysis.

## 3. Results

### 3.1. HFD Induces Disturbances in Liver Lipid Metabolism and Microbial Activity of Cecum Contents in Mice

**Figure 2:**
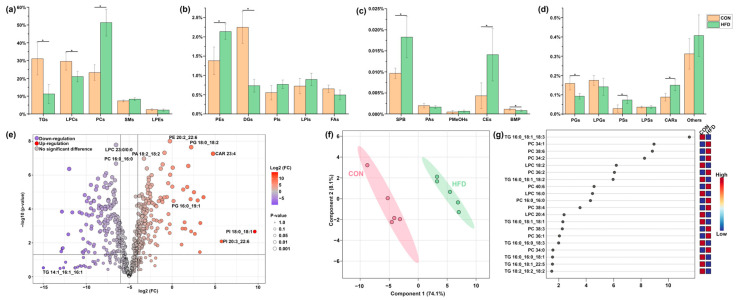
(**a**–**d**) Bar plots showing the content of lipidic species in the liver of mice following a high-fat diet (HFD). “*” indicates statistical significance (*p* < 0.05). (**e**) Volcano plot depicting the differential expression of lipids in the liver of mice between groups. (**f**) PLS-DA model of liver lipids in mice between groups. (**g**) VIP (variable importance in projection) score plot of the PLS-DA model for liver lipids in the CON and HFD groups, highlighting the top 20 lipids with VIP > 1.

A total of 599 lipids were characterized in the livers of the CON and HFD groups, as detailed in Table S1. These included 118 phosphatidylcholines (PCs), 69 phosphatidylinositols (PIs), 71 phosphatidylethanolamines (PEs), 33 phosphatidylglycerols (PGs), 5 phosphatidic acids (PAs), 5 phosphatidylserines (PSs), 54 SMs, 65 TGs, 35 diglycerides (DGs), 2 cholesteryl esters (CEs), 3 lyso-phosphatidylserines (LPSs), 13 lyso-phosphatidylinositols (LPIs), 11 lyso-phosphatidylglycerols (LPGs), 22 lyso-phosphatidylethanolamines (LPEs), 42 LPCs, 23 fatty acids (FAs), 18 acylcarnitines (CARs), 4 phosphatidylmethanols (PMeOHs), 1 sphingoid base (SPB), 1 bis (monoacylglycero)phosphate (BMP), and 4 others. As shown in Figure 2a–d, the levels of PCs, PEs, SPB, CEs, PSs, and CARs were significantly higher (*p* < 0.05) in the HFD group compared to the CON group. In contrast, the CON group exhibited higher levels of TGs, LPCs, DGs, PGs, and BMP than the HFD group. A volcano plot was generated to facilitate the analysis of liver lipids in the CON and HFD groups, with thresholds set at fold change (FC) > 2 and *p* < 0.05; the results are shown in Table S2. As demonstrated in Figure 2e, a total of 168 lipids were found to be upregulated, while 154 lipids were found to be downregulated in the HFD group in comparison to the CON group. To further investigate the effects of HFD on the lipidomic profiles of the liver, the data were subjected to multivariate statistical analysis, and a PLS-DA model was constructed to visualize the data, as shown in Figure 2f,g. The PLS-DA score plot (Figure 2f) clearly separated the lipid compositions of the livers of mice on a normal diet and those on HFD. The VIP score plot of the PLS-DA model identified 27 lipids with VIP > 1, including LPC 16:0_0:0, LPC 18:2, LPC 20:4_0:0, LPE 18:1, PC 16:0_16:0, PC 32:1, PC 34:0, PC 34:1, PC 34:2, PC 34:3, PC 36:1, PC 36:2, PC 36:3, PC 38:3, PC 38:4, PC 38:6, PC 40:6, PC 40:7, PC 42:10, TG 16:0_16:0_18:1, TG 16:0_16:0_18:3, TG 14:0_18:2_18:2, TG 16:0_18:1_18:1, TG 16:0_18:1_18:2, TG 16:0_18:1_18:3, TG 18:2_18:2_18:2, and TG 16:0_18:1_22:5. Among these, PCs were identified as the most significant lipids contributing to the distinction between the HFD and CON groups, while TGs were the most important lipids in the CON group. Figure 2g presents the top 20 lipids with VIP > 1.

**Figure 3:**
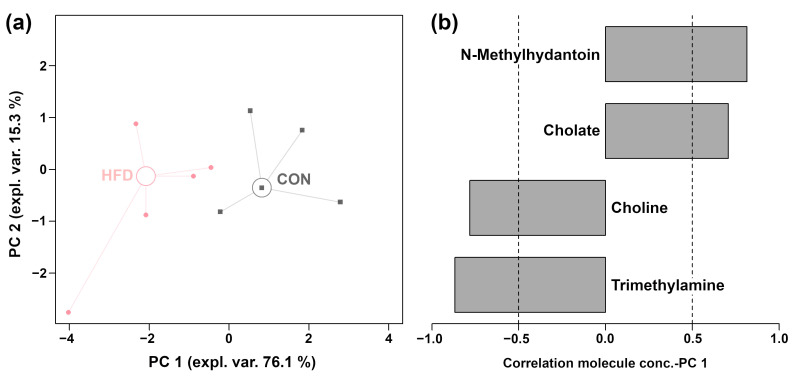
rPCA models were constructed using molecules that exhibited significant concentration differences in the cecum contents between the CON and HFD groups. The score plot (**a**) displays the two sample groups: Squares represent the CON group, and circles represent the HFD group. A large empty circle indicates the median for each group. The loading plot (**b**) highlights significant (*p* < 0.05) correlations between the importance of molecules on principal component 1 (PC 1) and their concentrations.

To understand in detail the effect of HFD on gut metabolism in obese mice. Following the approach of Zhou et al. [27], an rPCA model was constructed to investigate the overall metabolomic profiles of cecum contents in mice from the HFD and CON groups, as shown in Figure 3. In the rPCA model, principal component 1 (PC 1) represented 76.1% of the total variance in the dataset, effectively capturing the metabolomic differences between the two groups (Figure 3a). Compared to the CON group, cecum contents from the HFD group were characterized by significantly higher (*p* < 0.05) concentrations of choline and trimethylamine, as well as significantly lower (*p* < 0.05) concentrations of N-methylhydantoin and cholate (Figure 3b)

**Figure 4:**
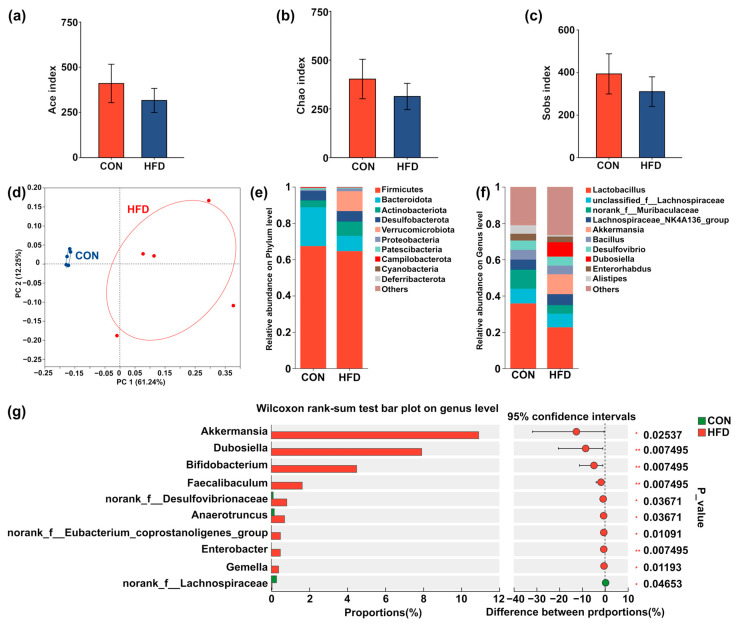
Effects of HFD on the microbiological community in the cecum contents of mice. (**a**–**c**) The α-diversity of the cecum contents microbiota, assessed using the Ace, Chao, and Sobs indices. (**d**) Principal coordinate analysis (PCoA) model based on the *β*-diversity of the CON and HFD groups. (**e**) Microbiota composition at the level of phylum (**f**) genus in the CON and HFD groups. (**g**) Comparison of significantly altered genera between the CON and HFD groups. “*” and “**” represent *p* < 0.05 and *p* < 0.01, respectively.

A total of 3614 amplicon sequence variants (ASVs) were obtained from the cecum contents samples of the CON and HFD groups. The Ace, Chao, and Sobs indices calculated on the HFD group were lower than the CON group (Figure 4a–c). *β*-Diversity analysis was employed to differentiate the microbial profiles of the cecum contents between the two groups, as shown in Figure 4d. The results from the PCoA model revealed that the microbiological profile of the HFD group was markedly different from the one of the CON group. As illustrated in Figure 4e, *Bacteroidetes* and *Firmicutes* were identified as the dominant phyla in the samples from the cecum. Specifically, the relative abundance of *Bacteroidetes* and *Firmicutes* in the CON group accounted for over 80% of the total phylum composition. Notably, the relative abundance of *Firmicutes* in both the CON and HFD groups exceeded 60%. Furthermore, Figure 4g shows that the abundance of genera such as *Akkermansia*, *Dubosiella*, *Bifidobacterium*, *Faecalibaculum*, *norank_f__Desulfovibrionaceae*, *Anaerotruncus*, *norank_f__Eubacterium_coprostanoligenes_group*, *Enterobacter*, and *Gemella* was significantly higher in the HFD group compared to the CON group (*p* < 0.05). Conversely, the abundance of *norank_f__Lachnospiraceae* was lower, again significantly (*p* < 0.05), in the HFD group.

### 3.2. Polysaccharide Dietary Intervention Remodels Liver Lipid Profiles and Microbial Community Composition of Cecum Contents in Obese Mice on HFD

**Figure 5:**
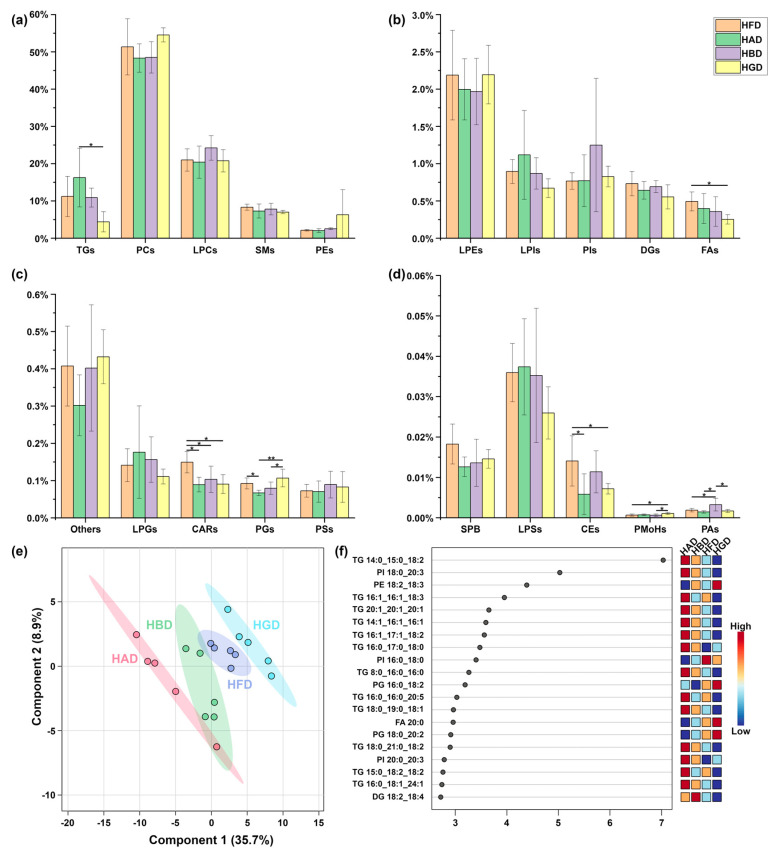
(**a**–**d**) Bar plots showing the lipid species content in the liver of mice following HFD induction. (**e**) PLS-DA model of liver lipid molecules in mice after HFD induction. (**f**) VIP score plots from the PLS-DA model of liver lipids in the CON and HFD groups. “*” and “**” represent *p* < 0.05 and *p* < 0.01, respectively.

We investigated the effects of BSP and/or composite polysaccharide dietary intervention on liver lipid metabolism in HFD-induced obese mice when obesity was alleviated. Similarly, liver lipids were analyzed in the HFD and polysaccharide intervention groups. A total of 595 lipids were characterized in the livers of the HFD, HAD, HBD, and HGD groups, as shown in Table S3. These included 119 PCs, 68 PIs, 33 PGs, 70 PEs, 4 PAs, 5 PSs, 53 SMs, 68 TGs, 32 DGs, 2 CEs, 3 LPSs, 13 LPIs, 11 LPGs, 22 LPEs, 42 LPCs, 23 FAs, 18 CARs, 3 PMeOHs, 1 SPB, and 5 others. In the livers of mice in the HFD group and following polysaccharide dietary intervention, PCs and LPCs were identified as the major lipid species. As shown in Figure 5a–d, after polysaccharide dietary intervention, a significant decrease in the levels of FAs, CEs, and CARs was observed in the HGD group relative to the HFD group.

To further explore the effects of polysaccharide intervention on the liver of HFD-induced obese mice, multivariate statistical analysis was performed on the lipidomic data. A PLS-DA model was constructed for data visualization, as shown in Figure 5e,f. After polysaccharide dietary intervention, distinct separations were observed between the HAD and HGD groups and the HFD group, while the HBD group partially overlapped with the HFD group (Figure 5e). The VIP score plots from the PLS-DA analysis revealed 113 lipids with VIP > 1 (Table S4). Notably, TGs, PIs, PGs, and PEs were identified as the most important lipids for differentiating the polysaccharide diet group post-intervention. Furthermore, ANOVA analysis identified 114 lipids as significantly (*p* < 0.05) different among the groups (Table S5). Among these, 46 potential lipid biomarkers (*p* < 0.05, VIP > 1) were identified, including TG 18:1_18:1_28:1, TG 19:0_18:1_18:1, TG 17:0_18:1_20:1, TG 18:0_18:1_18:1, TG 17:0_18:2_18:2, TG 17:0_18:1_18:1, TG 16:0_18:1_18:3, TG 15:0_18:1_18:1, TG 16:0_16:0_18:3, TG 15:0_16:1_18:2, TG 16:0_16:0_17:1, TG 12:0_12:0_12:0, SM 58:3;2O, PMeOH 16:0_22:6, PI 20:1_20:3, PI 20:0_20:4, PI 20:0_20:3, PI 18:0_22:3, PI 18:0_20:3, PI 17:0_20:4, PI 16:0_18:2, PI 16:0_18:0, PG O-18:2_18:1, PG O-16:1_18:1, PG 18:0_20:2, PG 16:0_20:4, PG 18:0_18:1, PG 17:0_18:2, PG 17:0_18:1, PG 16:0_18:2, PG 16:0_18:1, PE O-16:1_20:3, PE O-16:1_18:2, PE 20:0_22:6, PE 18:0_22:5, PE 17:0_22:6, PE 19:0_20:4, PE 18:1_20:4, PE 18:2_18:3, PE 16:0_20:3, PE 16:0_18:2, PC 44:4, PA 16:0_18:2, PA 16:0_16:0, dodecylbenzenesulfonic acid, and FA 20:0.

Of the 72 molecules characterized in the cecum contents (Table S6), 2 metabolite molecules were found to be significantly (*p* < 0.05) different in concentration between the HFD, HAD, HBD, and HGD groups (Table 2). Specifically, relative to the HAD group, mannose was significantly (*p* < 0.05) higher in the HGD group. Sarcosine concentrations were significantly lower in the HFD group than in the HAD and HGD groups.

**Figure 6:**
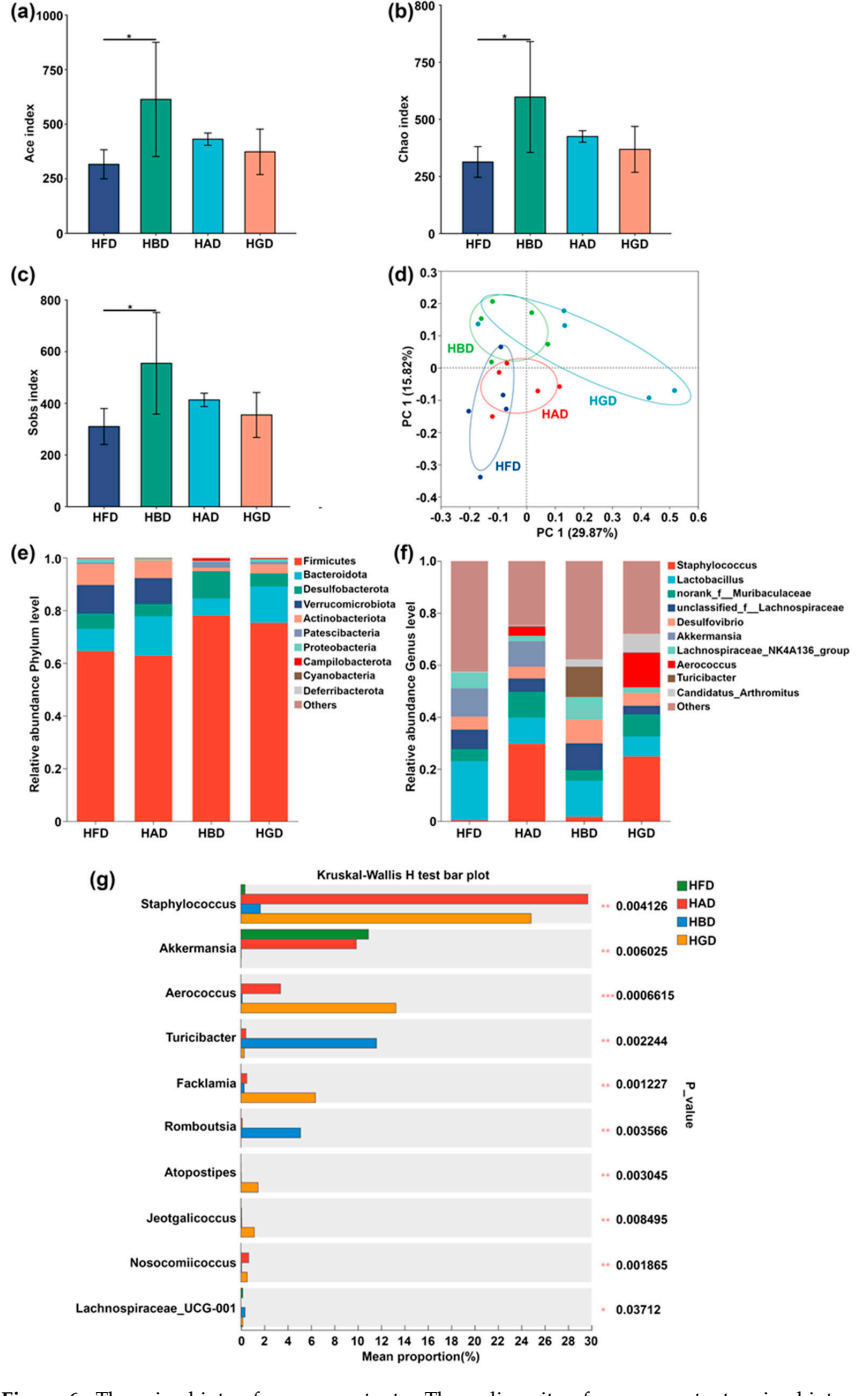
The microbiota of cecum contents. The *α*-diversity of cecum contents microbiota was assessed using the Ace (**a**), Chao (**b**), and Sobs (**c**) indices. PCoA based on *β*-diversity for the HFD, HAD, HBD, and HGD groups is shown in (**d**). Microbiota composition of these groups at the phylum (**e**) and genus (**f**) levels is also depicted. Panel (**g**) compares significantly altered genera among the groups. Asterisks (*), (**), and (***) indicate *p* < 0.05, *p* < 0.01 and *p* < 0.001, respectively.

A total of 6514 amplicon sequence variants (ASVs) were identified from the cecum contents of the HFD, HAD, HBD, and HGD groups. In relation to the HFD group, the indices of Ace, Sobs, and Chao were significantly higher in the HBD group (*p* < 0.05), as shown in Figure 6a–c. *β*-diversity analysis was considered to differentiate the profiles of microorganisms between groups, as depicted in Figure 6d. The results from the PCoA model showed a clear separation of the HGD and HFD groups, indicating distinct microbiological profiles in the cecum contents. Notably, differences between the groups appeared at various levels after polysaccharide treatment.

As shown in Figure 6e, *Firmicutes* and *Bacteroidetes* were identified as the dominant phyla in the cecum contents of all groups. The relative abundance of *Firmicutes* exceeded 60% in all groups. Additionally, in comparison to the HFD and HAD groups, *Verrucomicrobiota’s* relative abundance was significantly lower in all groups. At the level of the genus, the relative abundances of *Staphylococcus* and *Aerococcus* were also significantly lower in both the HGD and HAD groups when compared to the HFD group, as shown in Figure 6f.

To further investigate the impact of polysaccharide supplementation on the microbial community in the cecum contents of HFD-induced obese mice, microbial community analysis was conducted at the genus level (Figure 6g). Notably, among the four groups, *Akkermansia* reached the highest abundance in the HFD group. The HAD group exhibited the highest abundances of Staphylococcus and *Nosocomiicoccus*, while *Turicibacter* and *Romboutsia* reached the highest abundance in the HBD group. The HGD group showed the highest levels of *Aerococcus*, *Facklamia*, *Atopostipes*, and *Jeotgalicoccus*.

### 3.3. Formatting of Mathematical Components

**Figure 7:**
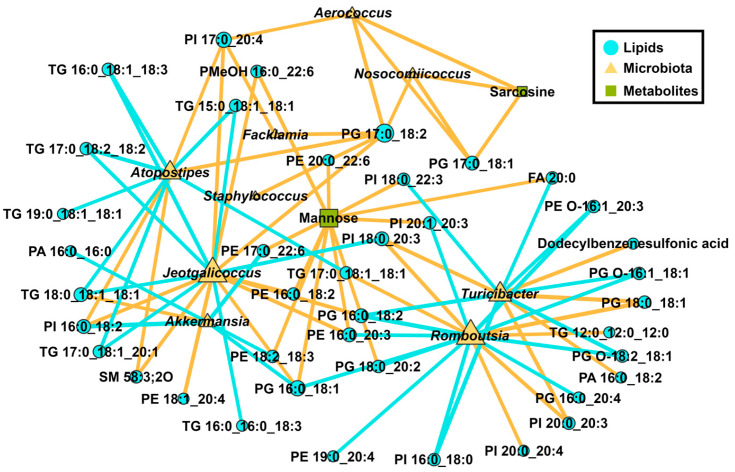
Network analysis of liver lipids, cecum contents microbiome, and metabolome in mice treated with polysaccharide intervention. Positive and negative correlations are highlighted by orange and blue lines, respectively.

To better understand the relationship between gut microbiota and liver following polysaccharide intervention, network analyses were conducted on liver lipid biomarkers, differential metabolites in cecum contents, and differential microbial communities in the cecum contents of the polysaccharide intervention and HFD groups, retaining only strong (|cor| > 0.5) and statistically significant (*p* < 0.05) associations, as shown in Figure 7. Specifically, a negative correlation was observed between *Atopostipes* and most of the TGs, whereas a positive correlation was found between *Atopostipes* and PI 17:0_20:4, SM 58:3;2O, and PI 16:0_18:2. Similarly, *Jeotgalicoccus* was negatively correlated with most TGs but positively correlated with PI 17:0_20:4, PMeOH 16:0_22:6, PI 16:0_18:2, SM 58:3;2O, PG 16:0_18:2, PE 18:1_20:4, PG 16:0_18:1, PE 16:0_20:3, and PG 17:0_18:2. *Akkermansia* was positively correlated with TG 18:0_18:1_18:1, while showing a negative correlation with PA 16:0_16:0, PI 16:0_18:2, PG 16:0_18:1, PE 18:2_18:3, and PE 17:0_22:6. Additionally, positive correlations were observed between *Aerococcus* and *Nosocomiicoccus* with PG 17:0_18:2, PG 17:0_18:1, and sarcosine. Interestingly, *Romboutsia* showed a positive correlation with TGs (TG 12:0_12:0_12:0 and TG 17:0_18:1_18:1), PIs (PI 20:0_20:3, PI 20:0_20:4, and PI 18:0_20:3), and PG 18:0_18:1. In contrast, *Romboutsia* exhibited a negative correlation with most of the PGs, as well as PI 20:1_20:3, PI 16:0_18:0, PE 19:0_20:4, PE 16:0_20:3, and PE O-16:1_20:3. Lastly, *Turicibacter* was positively correlated with PG 18:0_18:1, PA 16:0_18:2, PI 20:0_20:3, PI 18:0_20:3, and *dodecylbenzenesulfonic* acid. However, *Turicibacter* was negatively correlated with PI 18:0_22:3, PI 16:0_18:0, FA 20:0, PE O-16:1_20:3, PG 16:0_18:2, PG O-16:1_18:1, and PG O-18:2_18:1.

## 4. Discussion

Dietary interventions have gained widespread recognition as an effective and safe strategy for managing obesity [28]. Plant polysaccharides are highly regarded in dietary interventions for their ability to alleviate HFD-induced gut microbial dysbiosis [29]. While previous studies have identified the *Bletilla striata* polysaccharide (BSP) as a potential natural anti-obesity agent [13], its specific effects on cecum microbiota composition and liver lipid regulation in HFD-induced obese mice remain insufficiently explored. This study systematically investigates the regulatory roles of BSP and/or composite polysaccharides in modulating liver lipid metabolism and gut microbiome dynamics in HFD-fed obese mice.

Dysregulation of the metabolism of lipids is a key characteristic of many metabolic disorders, contributing to the inefficient processing of lipids that leads to the development of obesity and related metabolic disorders [30]. The liver, a critical organ responsible for energy metabolism, plays a pivotal role in maintaining the homeostasis of lipids through the regulation of the synthesis and catabolism of lipids, including cholesterol, free fatty acids (FFAs), and TGs. In the present study, the levels of PCs, PEs, CEs, and PSs were significantly higher in the HFD group compared to the CON group, in agreement with previous findings [21]. PCs, PEs, PSs, and sphingomyelin (SM) are key components of glycerophospholipids (GPLs), the major lipid constituents of animal cell membranes. Some PEs have been shown to induce liver injury through mechanisms such as promoting lipid accumulation, disrupting mitochondrial function, inhibiting cell growth, generating oxidative stress, and activating liver stellate cells [31]. Additionally, an increased abundance of PEs has been associated with the enhanced aggregation of lipid droplets, contributing to larger droplet sizes [21]. PCs are linked to primary biliary cholangitis and subclinical atherosclerosis in type 1 diabetes patients [32]. Furthermore, PSs are known to enhance *β*-oxidation by activating the peroxisome proliferator-activated receptor alpha (PPAR-*α*), suggesting that certain PS species may act as potent PPAR agonists to promote *β*-oxidation [33].

Sphingolipids, which contain SPB, play a crucial structural role in membrane fluidity and the regulation of lipid raft substructures [34]. SPB is produced by the catabolism of ceramides via acid ceramidase, and the abnormal accumulation of ceramides has been linked to negative effects on glucose tolerance and lipid metabolism [35]. Supplementation with BSP and/or composite polysaccharides led to reduced levels of CARs, potentially preventing oxidative stress and mitochondrial dysfunction, which can impair insulin signaling pathways [36]. Furthermore, the supplementation of BSP and composite polysaccharides may exert anti-obesity effects by inhibiting CEs biosynthesis, thus alleviating liver steatosis [37]. Fatty acids (FAs) are crucial components of membrane lipids and are involved in energy metabolism and signaling pathways. The synthesis of endogenous FAs is catalyzed by stearoyl-CoA desaturase 1 (SCD1) and fatty acid synthase (FAS) to form TGs [38]. PSs and PGs are endogenous phospholipids capable of inducing anti-inflammatory responses. PGs, in particular, serve as precursors for cardiolipin (CL) synthesis, a mitochondrial-specific phospholipid that is essential for maintaining mitochondrial function. A deficiency of PGs in mammalian cells leads to CL depletion, mitochondrial dysfunction, and reduced ATP production [39].

Research has shown that an excess of choline can lead to the accumulation of adipose tissue. Additionally, the gut microbiota metabolizes choline and L-carnitine to produce trimethylamine, which is then absorbed into the bloodstream and rapidly oxidized by liver enzymes to trimethylamine N-oxide (TMAO). Both trimethylamine and TMAO have been implicated in promoting the development of the fatty liver. Elevated levels of TMAO and choline have also been associated with an increased cardiometabolic risk profile [40]. N-methylhydantoin is primarily produced by gut microorganisms metabolizing creatinine [41]. Sarcosine can be synthesized through further metabolism of N-methylhydantoin and creatine or via choline metabolism, which avoids microbial degradation and is eventually oxidized to betaine. Betaine is then converted to N, N-dimethylglycine, which is subsequently converted into sarcosine [42]. Choline is a basic constituent of lecithin, which is important as a precursor of acetylcholine as a methyl donor in lipid metabolism [43]. The current study revealed elevated levels of N-methylhydantoin and sarcosine in both the standard diet and polysaccharide diet groups, suggesting that BSP and/or composite polysaccharides may enhance gut microbiota metabolism, contributing to anti-obesity effects.

The impact of a long-term HFD on the gut microbiome has been extensively studied, with evidence indicating that such a diet can significantly disrupt the microbiome composition [32]. In contrast, polysaccharide supplementation has been shown to modulate the composition and balance of the gut microbiome [4]. The present study found that BSP and/or composite polysaccharide treatment improved the gut microbiome, although with some variations in the microbiome involved. The Ace, Chao, and Sobs indices indicated that BSP significantly increased gut microbiota’s diversity and abundance compared to the HFD group. At the phylum level, *Firmicutes* and *Bacteroidota* were the predominant microorganisms in the cecum contents, which contributed to dietary fiber and polysaccharide digestion [44]. Firmicutes could promote calorie absorption and weight gain, while *Bacteroidota* is crucial in carbohydrate metabolism. *Bacteroidota* also enhances the production of short-chain fatty acids (SCFAs), which exhibit anti-inflammatory effects through host metabolism. At the genus level, BSP and/or composite polysaccharide administration elevated the abundance of *Dubosiella*, *Bifidobacterium*, *Faecalibaculum*, *norank_f__Desulfovibrionaceae*, *Anaerotruncus*, *norank_f__Eubacterium_coprostanoligenes_group*, Enterobacter, and *Gemella* in the cecum contents of HFD-induced obese mice. In contrast, the HFD significantly reduced the abundance of *norank_f__Lachnospiraceae*. These microorganisms may be considered potential probiotic candidates supplemented with anti-obesity and anti-inflammatory properties.

Increased relative abundance of *Jeotgalicoccus*, despite being less extensively documented in the literature, seems to have a positive impact in animal models fed HFD [45]. *Turicibacter*, a member of the order *Bifidobacteria*, is a common intestinal probiotic. The abundance of *Turicibacter* is significantly reduced in obese individuals and their offspring. Prolonged HFD feeding in mice results in obesity and insulin resistance, accompanied by a reduced relative abundance of *Anaeroplasma* and *Turicibacter*. Consumption of spotted beans has been shown to ameliorate HFD-induced obesity and insulin resistance by increasing the abundance of *Ruminococcaceae*, *Turicibacter*, and Lactobacillus [46]. *Gemella* can produce short-chain fatty acids (SCFAs) [47]. *Lachnospiraceae_UCG-001* produces SCFAs, and alterations in the composition of the gut microbiota, including this genus, have been linked to the suppression of colonic inflammation and tumorigenesis [48]. We noted, in the present study, an increase in the abundance of *Atopostipes* following composite polysaccharide dietary intervention compared to the HFD group. Zhang et al. also reported a high abundance of *Atopostipes* in mice on a normal diet compared to hyperlipidemic mice. This suggests that *Atopostipes* may play a beneficial role in conditions associated with obesity induced by HFD [49].

There is growing evidence indicating that fluctuations in the gut microbiome significantly influence liver function and the development of metabolic disorders, including diabetes and hyperlipidemia [50]. Network analysis revealed a negative correlation between *Atopostipes* and *Jeotgalicoccus* and most triglycerides (TGs). Consistent with our results, Hu et al. also found a negative correlation between the relative abundance of *Jeotgalcoccus* and *Atopostipes* and TG levels in their investigation of the effect of supplementation with Lactobacillus plantarum FZU3013 fermented kelp on the prevention of *hyperlipidaemia* in rats fed an HFD [51]. The anti-obesity effects of BSP and/or composite polysaccharide intervention may be linked to the promotion of *Atopostipes* and *Jeotgalicoccus* abundance, which in turn reduces liver triglyceride content. In the study, the findings elucidate the anti-obesity mechanisms of polysaccharides through gut microbiota remodeling and cross-tissue metabolic interactions by means of a combination of lipidomics, metabolomics, and microbiomics. Other omics approaches (i.e., transcriptomics and proteomics) could be involved to further investigate the anti-obesity mechanisms of BSP.

## 5. Conclusions

The mechanisms that regulate gut microbiota and liver lipid metabolism are key to alleviating obesity and related diseases. In this study, BSP and/or composite polysaccharides were shown to ameliorate obesity in HFD-fed mice by modulating liver lipid metabolism and gut microbiota composition. These interventions not only reduced liver CEs, FAs, and CARs but also restored microbial diversity in cecum contents, enriched beneficial microbiota, and decreased levels of anti-obesity metabolites sarcosine. These findings shed light on the multi-target mechanism of BSP through the “gut–liver axis” and underscore its potential as a natural therapeutic agent for obesity.

## Figures and Tables

**Table 1:** The elution procedure of liquid phase.

Time (min)	Aqueous Phase %	Organic Phase %
0.0	40	60
2	43	57
2.1	50	50
12	60	40
12.1	75	25
18	99	1
19	99	1
20	40	60
25	40	60

**Table 2:** Molecule concentrations (mmol/L) in cecum contents characterized by 1H-NMR as significantly different among the groups.

Compound Name	Molecular Concentration			
	HFD	HAD	HBD	HGD
Mannose	9.78×10^−5^ ± 2.24 × 10^−5 ab^	5.00×10^−5^ ± 3.80 × 10^−5 b^	1.10×10^−4^ ± 3.12 × 10^−5 ab^	1.69×10^−4^ ± 8.41 × 10^−5 a^
Sarcosine	1.09×10^−4^ ± 3.47 × 10^−5 b^	4.40×10^−4^ ± 1.88 × 10^−4 a^	2.32×10^−4^ ± 1.27 × 10^−4 ab^	3.92×10^−4^ ± 2.68 × 10^−4 a^

Lower case letters represent significant differences (*p* < 0.05).

## Data Availability

The original contributions presented in this study are included in the article/Supplementary Materials. Further inquiries can be directed to the corresponding author.

## Supplementary Materials

The following supporting information can be downloaded at https://www.mdpi.com/article/10.3390/foods14152653/s1. Table S1: The relative content of lipids in CON and HFD groups (Mean ± SD). Table S2: Results of volcano maps for CON and HFD groups. Table S3: The relative content of lipids in HFD, HAD, HBD, and HGD groups (Mean ± SD). Table S4: VIP score plots of PLS-DA showed a total of 113 lipids for VIP > 1. Table S5: ANOVA analysis lipids were found to be significantly different between HFD, HAD, HBD, and HGD groups. Table S6: Concentrations of metabolites in mice cecum content from CON, HFD, HAD, HBD, and HGD groups.
