# Supplementary material for: Dietary Interventions with Bletilla striata Polysaccharides and/or Composite Polysaccharides Remodel Liver Lipid Profiles and Ameliorate Gut Metabolic Disturbances in High-Fat Diet-Induced Obese Mice
Source: Foods. 2025 Jul 29;14(15):2653. doi: 10.3390/foods14152653 (PMC12345948; doi:10.3390/foods14152653)
Supplement: Supplementary file 1 [file foods-14-02653-s001.zip › foods-3744931-supplementary.pdf]

Table S1. The relative content of lipids in CON and HFD groups (Mean ± SD)

Name	CON	HFD
TG 72:2|TG 36:0_18:1_18:1	$1.97\times10^{-6}\pm1.69\times10^{-6}$	$3.82\times10^{-6}\pm2.43\times10^{-6}$
TG 70:3|TG 18:1_18:1_34:1	$1.10\times10^{-5}\pm3.85\times10^{-6}$	$2.76\times10^{-5}\pm1.86\times10^{-5}$
TG 70:2|TG 18:0_18:1_34:1	$4.76\times10^{-6}\pm3.21\times10^{-6}$	$1.05\times10^{-5}\pm7.34\times10^{-6}$
TG 70:2|TG 16:0_18:1_36:1	$4.67\times10^{-6}\pm2.78\times10^{-6}$	$9.63\times10^{-6}\pm6.30\times10^{-6}$
TG 64:3|TG 18:1_18:1_28:1	$2.98\times10^{-6}\pm3.27\times10^{-6}$	-
TG 62:2|TG 26:0_18:1_18:1	$1.01\times10^{-5}\pm7.76\times10^{-6}$	$1.36\times10^{-7}\pm2.72\times10^{-7}$
TG 62:2|TG 16:0_18:1_28:1	$7.89\times10^{-6}\pm7.28\times10^{-6}$	-
TG 60:3|TG 20:1_20:1_20:1	$2.23\times10^{-4}\pm8.44\times10^{-5}$	$3.70\times10^{-5}\pm6.20\times10^{-5}$
TG 60:3|TG 18:1_18:1_24:1	$2.23\times10^{-4}\pm8.08\times10^{-5}$	$6.29\times10^{-5}\pm7.51\times10^{-5}$
TG 58:5|TG 22:1_18:2_18:2	$6.28\times10^{-4}\pm2.17\times10^{-4}$	$2.27\times10^{-4}\pm1.20\times10^{-4}$
TG 58:3|TG 18:1_18:1_22:1	$6.09\times10^{-4}\pm1.20\times10^{-4}$	$2.72\times10^{-4}\pm2.02\times10^{-4}$
TG 58:2|TG 18:0_18:1_22:1	$7.70\times10^{-4}\pm3.30\times10^{-4}$	$1.23\times10^{-4}\pm1.10\times10^{-4}$
TG 58:2|TG 16:0_20:1_22:1	$9.35\times10^{-4}\pm3.77\times10^{-4}$	$1.56\times10^{-4}\pm1.16\times10^{-4}$
TG 58:2|TG 16:0_18:1_24:1	$7.74\times10^{-4}\pm1.26\times10^{-4}$	$3.08\times10^{-5}\pm6.17\times10^{-5}$
TG 57:2|TG 18:0_21:0_18:2	$1.58\times10^{-4}\pm4.44\times10^{-5}$	$2.07\times10^{-5}\pm1.51\times10^{-5}$
TG 56:6|TG 16:0_18:1_22:5	$1.14\times10^{-2}\pm2.83\times10^{-3}$	$2.43\times10^{-3}\pm1.55\times10^{-3}$
TG 56:2|TG 20:0_18:1_18:1	$3.06\times10^{-3}\pm6.56\times10^{-4}$	$8.89\times10^{-4}\pm2.77\times10^{-4}$
TG 56:2|TG 16:0_18:1_22:1	$2.64\times10^{-3}\pm5.86\times10^{-4}$	$9.61\times10^{-4}\pm4.28\times10^{-4}$
TG 56:1|TG 18:0_20:0_18:1	$7.27\times10^{-4}\pm2.14\times10^{-4}$	$2.71\times10^{-4}\pm1.10\times10^{-4}$
TG 56:1|TG 16:0_22:0_18:1	$1.03\times10^{-3}\pm3.28\times10^{-4}$	$1.06\times10^{-4}\pm8.87\times10^{-5}$
TG 55:5|TG 19:1_18:2_18:2	$2.72\times10^{-4}\pm1.87\times10^{-4}$	$6.18\times10^{-5}\pm3.47\times10^{-5}$
TG 55:2|TG 19:0_18:1_18:1	$5.80\times10^{-4}\pm2.43\times10^{-4}$	$1.52\times10^{-4}\pm1.05\times10^{-4}$
TG 55:2|TG 17:0_18:1_20:1	$6.74\times10^{-4}\pm3.04\times10^{-4}$	$2.29\times10^{-4}\pm1.35\times10^{-4}$

TG 55:1|TG 18:0_19:0_18:1	$1.24\times10^{-4}\pm7.71\times10^{-5}$	$4.11\times10^{-6}\pm5.26\times10^{-6}$
TG 54:7|TG 18:2_18:2_18:3	$5.35\times10^{-3}\pm1.61\times10^{-3}$	$3.03\times10^{-4}\pm2.02\times10^{-4}$
TG 54:6|TG 18:2_18:2_18:2	$9.06\times10^{-3}\pm5.16\times10^{-3}$	$1.43\times10^{-3}\pm9.67\times10^{-4}$
TG 54:4|TG 18:1_18:1_18:2	$9.12\times10^{-3}\pm5.91\times10^{-3}$	$4.84\times10^{-3}\pm2.52\times10^{-3}$
TG 54:3|TG 18:0_18:1_18:2	$9.39\times10^{-3}\pm4.89\times10^{-3}$	$6.47\times10^{-3}\pm3.06\times10^{-3}$
TG 54:2|TG 18:0_18:1_18:1	$7.66\times10^{-3}\pm1.48\times10^{-3}$	$7.45\times10^{-3}\pm3.31\times10^{-3}$
TG 54:1|TG 18:0_18:0_18:1	$1.96\times10^{-3}\pm5.95\times10^{-4}$	$1.87\times10^{-3}\pm3.44\times10^{-4}$
TG 54:1|TG 16:0_20:0_18:1	$2.22\times10^{-3}\pm5.88\times10^{-4}$	$1.64\times10^{-3}\pm2.96\times10^{-4}$
TG 53:5|TG 17:1_18:2_18:2	$4.09\times10^{-4}\pm4.50\times10^{-4}$	$3.19\times10^{-5}\pm2.32\times10^{-5}$
TG 53:4|TG 17:0_18:2_18:2	$1.21\times10^{-3}\pm9.51\times10^{-4}$	$2.88\times10^{-4}\pm1.06\times10^{-4}$
TG 53:2|TG 17:0_18:1_18:1	$3.67\times10^{-3}\pm1.35\times10^{-3}$	$1.78\times10^{-3}\pm1.35\times10^{-3}$
TG 53:2|TG 16:0_18:1_19:1	$2.89\times10^{-3}\pm1.03\times10^{-3}$	$1.60\times10^{-3}\pm7.23\times10^{-4}$
TG 53:1|TG 16:0_19:0_18:1	$3.09\times10^{-4}\pm1.90\times10^{-4}$	$2.30\times10^{-4}\pm2.17\times10^{-4}$
TG 52:6|TG 16:1_18:2_18:3	$1.94\times10^{-3}\pm1.09\times10^{-3}$	$1.31\times10^{-4}\pm1.03\times10^{-4}$
TG 52:5|TG 16:0_16:0_20:5	$5.50\times10^{-5}\pm1.85\times10^{-5}$	$2.39\times10^{-5}\pm2.00\times10^{-5}$
TG 52:4|TG 16:0_18:1_18:3	$7.97\times10^{-2}\pm3.08\times10^{-2}$	$1.68\times10^{-2}\pm8.78\times10^{-3}$
TG 52:3|TG 16:0_18:1_18:2	$5.82\times10^{-2}\pm1.06\times10^{-2}$	$2.53\times10^{-2}\pm1.30\times10^{-2}$
TG 52:2|TG 16:0_18:1_18:1	$3.74\times10^{-2}\pm3.57\times10^{-3}$	$2.44\times10^{-2}\pm9.46\times10^{-3}$
TG 52:1|TG 16:0_18:0_18:1	$6.32\times10^{-3}\pm2.32\times10^{-3}$	$4.15\times10^{-3}\pm1.87\times10^{-3}$
TG 51:4|TG 16:1_17:1_18:2	$7.84\times10^{-4}\pm7.24\times10^{-4}$	$4.73\times10^{-5}\pm4.51\times10^{-5}$
TG 51:4|TG 15:0_18:2_18:2	$7.41\times10^{-4}\pm6.11\times10^{-4}$	$6.72\times10^{-5}\pm4.71\times10^{-5}$
TG 51:2|TG 15:0_18:1_18:1	$3.45\times10^{-3}\pm1.39\times10^{-3}$	$8.95\times10^{-4}\pm4.76\times10^{-4}$
TG 51:1|TG 16:0_17:0_18:1	$1.09\times10^{-3}\pm6.46\times10^{-4}$	$2.42\times10^{-4}\pm1.01\times10^{-4}$
TG 51:0|TG 17:0_17:0_17:0	$6.11\times10^{-5}\pm1.00\times10^{-4}$	$1.31\times10^{-5}\pm1.36\times10^{-5}$
TG 51:0|TG 16:0_17:0_18:0	$1.22\times10^{-4}\pm1.28\times10^{-4}$	$4.08\times10^{-7}\pm8.17\times10^{-7}$
TG 51:0|TG 16:0_16:0_19:0	$5.12\times10^{-5}\pm7.73\times10^{-5}$	$2.43\times10^{-6}\pm4.84\times10^{-6}$

TG 50:5|TG 16:1_16:1_18:3	$1.11\times10^{-3}\pm1.58\times10^{-3}$	$2.83\times10^{-5}\pm2.36\times10^{-5}$
TG 50:4|TG 16:0_16:1_18:3	$3.71\times10^{-3}\pm3.44\times10^{-3}$	$2.67\times10^{-4}\pm2.32\times10^{-4}$
TG 50:4|TG 14:0_18:2_18:2	$5.43\times10^{-3}\pm5.83\times10^{-3}$	$3.93\times10^{-4}\pm2.35\times10^{-4}$
TG 50:3|TG 16:0_16:0_18:3	$1.28\times10^{-2}\pm5.91\times10^{-3}$	$2.44\times10^{-3}\pm2.15\times10^{-3}$
TG 50:1|TG 16:0_16:0_18:1	$1.02\times10^{-2}\pm5.59\times10^{-3}$	$2.33\times10^{-3}\pm5.85\times10^{-4}$
TG 50:0|TG 15:0_17:0_18:0	$1.09\times10^{-3}\pm7.72\times10^{-4}$	$3.74\times10^{-4}\pm5.34\times10^{-5}$
TG 49:3|TG 15:0_16:1_18:2	$2.94\times10^{-4}\pm4.57\times10^{-4}$	-
TG 49:1|TG 16:0_16:0_17:1	$6.36\times10^{-4}\pm8.66\times10^{-4}$	-
TG 49:0|TG 16:0_16:0_17:0	$2.09\times10^{-4}\pm3.41\times10^{-4}$	-
TG 48:3|TG 16:1_16:1_16:1	$1.85\times10^{-3}\pm2.43\times10^{-3}$	$5.60\times10^{-5}\pm4.78\times10^{-5}$
TG 48:3|TG 14:0_16:1_18:2	$1.91\times10^{-3}\pm2.79\times10^{-3}$	$6.67\times10^{-5}\pm4.03\times10^{-5}$
TG 48:0|TG 16:0_16:0_16:0	$1.69\times10^{-3}\pm3.13\times10^{-3}$	-
TG 46:3|TG 14:1_16:1_16:1	$2.07\times10^{-4}\pm3.57\times10^{-4}$	$1.27\times10^{-7}\pm2.54\times10^{-7}$
TG 46:3|TG 14:0_14:0_18:3	$3.36\times10^{-4}\pm5.83\times10^{-4}$	-
TG 46:2|TG 14:0_14:0_18:2	$7.93\times10^{-4}\pm1.35\times10^{-3}$	-
TG 24:0|TG 8:0_8:0_8:0	$4.01\times10^{-6}\pm3.45\times10^{-6}$	$6.49\times10^{-6}\pm1.78\times10^{-6}$
SPB 18:1;2O	$9.64\times10^{-5}\pm1.12\times10^{-5}$	$1.82\times10^{-4}\pm4.41\times10^{-5}$
SM 60:4;2O	-	$9.22\times10^{-6}\pm3.57\times10^{-6}$
SM 60:3;2O	$7.67\times10^{-6}\pm3.23\times10^{-6}$	$2.18\times10^{-5}\pm7.99\times10^{-6}$
SM 60:2;2O	$2.27\times10^{-5}\pm4.83\times10^{-6}$	$1.74\times10^{-5}\pm2.92\times10^{-6}$
SM 59:3;2O	$6.34\times10^{-6}\pm2.14\times10^{-6}$	$4.30\times10^{-6}\pm9.30\times10^{-7}$
SM 59:2;2O	$1.60\times10^{-5}\pm3.94\times10^{-6}$	$7.17\times10^{-6}\pm1.23\times10^{-6}$
SM 58:3;2O	$9.14\times10^{-6}\pm6.30\times10^{-6}$	$2.27\times10^{-5}\pm7.26\times10^{-6}$
SM 58:2;2O	$3.97\times10^{-5}\pm1.44\times10^{-5}$	$2.23\times10^{-5}\pm7.65\times10^{-6}$
SM 57:2;2O	$1.98\times10^{-5}\pm8.01\times10^{-6}$	$8.06\times10^{-6}\pm2.01\times10^{-6}$
SM 56:2;2O	$2.68\times10^{-5}\pm1.05\times10^{-5}$	$1.99\times10^{-5}\pm8.06\times10^{-6}$

SM 54:2;2O	$1.66\times10^{-8}\pm3.33\times10^{-8}$	$8.35\times10^{-6}\pm6.35\times10^{-6}$
SM 52:9;2O	$6.90\times10^{-6}\pm5.86\times10^{-6}$	$3.93\times10^{-6}\pm5.09\times10^{-6}$
SM 52:2;2O	$5.72\times10^{-6}\pm3.41\times10^{-6}$	$1.25\times10^{-5}\pm3.36\times10^{-6}$
SM 50:11;2O	$1.35\times10^{-5}\pm5.25\times10^{-6}$	$1.19\times10^{-5}\pm3.62\times10^{-6}$
SM 48:9;2O	$2.38\times10^{-5}\pm9.78\times10^{-6}$	$1.74\times10^{-5}\pm7.50\times10^{-6}$
SM 44:6;2O	$1.02\times10^{-4}\pm1.91\times10^{-5}$	$4.24\times10^{-5}\pm1.35\times10^{-5}$
SM 44:2;2O|SM 18:1;2O/26:1	$3.17\times10^{-5}\pm4.86\times10^{-6}$	$7.79\times10^{-5}\pm1.38\times10^{-5}$
SM 44:1;2O|SM 18:1;2O/26:0	$7.67\times10^{-5}\pm2.37\times10^{-5}$	$7.86\times10^{-5}\pm3.70\times10^{-5}$
SM 43:4;2O	$1.33\times10^{-4}\pm3.52\times10^{-5}$	$1.04\times10^{-4}\pm4.04\times10^{-5}$
SM 43:2;2O|SM 19:1;2O/24:1	$1.37\times10^{-4}\pm2.11\times10^{-5}$	$2.17\times10^{-4}\pm2.96\times10^{-5}$
SM 43:1;2O|SM 18:1;2O/25:0	$3.75\times10^{-4}\pm7.43\times10^{-5}$	$4.18\times10^{-4}\pm7.01\times10^{-5}$
SM 42:3;2O|SM 18:1;2O/24:2	$9.99\times10^{-4}\pm2.65\times10^{-4}$	$1.86\times10^{-3}\pm2.63\times10^{-4}$
SM 42:2;2O|SM 18:1;2O/24:1	$1.63\times10^{-2}\pm3.64\times10^{-3}$	$1.56\times10^{-2}\pm4.31\times10^{-3}$
SM 42:1;2O|SM 18:1;2O/24:0	$1.07\times10^{-2}\pm1.59\times10^{-3}$	$8.97\times10^{-3}\pm1.01\times10^{-3}$
SM 42:1;2O	$1.13\times10^{-4}\pm2.36\times10^{-5}$	$1.78\times10^{-4}\pm3.82\times10^{-5}$
SM 42:0;2O	$1.38\times10^{-4}\pm3.39\times10^{-5}$	$1.05\times10^{-4}\pm2.29\times10^{-5}$
SM 41:2;2O|SM 17:1;2O/24:1	$1.14\times10^{-3}\pm1.55\times10^{-4}$	$9.61\times10^{-4}\pm1.07\times10^{-4}$
SM 41:2;2O	$1.45\times10^{-3}\pm3.65\times10^{-4}$	$9.12\times10^{-4}\pm1.33\times10^{-4}$
SM 41:1;2O|SM 18:1;2O/23:0	$7.74\times10^{-3}\pm8.30\times10^{-4}$	$6.60\times10^{-3}\pm1.61\times10^{-3}$
SM 41:0;2O	$7.50\times10^{-5}\pm1.60\times10^{-5}$	$2.73\times10^{-5}\pm6.65\times10^{-6}$
SM 40:2;2O|SM 18:2;2O/22:0	$1.18\times10^{-3}\pm2.53\times10^{-4}$	$1.01\times10^{-3}\pm2.45\times10^{-4}$
SM 40:2;2O|SM 18:1;2O/22:1	$1.27\times10^{-3}\pm2.45\times10^{-4}$	$9.35\times10^{-4}\pm2.50\times10^{-4}$
SM 40:1;2O|SM 18:1;2O/22:0	$1.27\times10^{-2}\pm4.94\times10^{-3}$	$1.72\times10^{-2}\pm3.72\times10^{-3}$
SM 40:0;2O	$1.35\times10^{-4}\pm3.35\times10^{-5}$	$9.36\times10^{-5}\pm2.50\times10^{-5}$
SM 39:2;2O|SM 17:1;2O/22:1	$5.33\times10^{-5}\pm1.77\times10^{-5}$	-
SM 39:1;2O|SM 17:1;2O/22:0	$1.55\times10^{-3}\pm4.45\times10^{-4}$	$8.68\times10^{-4}\pm1.86\times10^{-4}$

SM 39:1;2O	$1.58\times10^{-3}\pm2.24\times10^{-4}$	$1.12\times10^{-3}\pm1.75\times10^{-4}$
SM 39:0;2O	$2.42\times10^{-4}\pm4.36\times10^{-5}$	$9.28\times10^{-5}\pm2.58\times10^{-5}$
SM 38:2;2O	$1.10\times10^{-5}\pm5.81\times10^{-6}$	$4.54\times10^{-5}\pm1.04\times10^{-5}$
SM 38:1;2O|SM 18:1;2O/20:0	$2.16\times10^{-3}\pm2.74\times10^{-4}$	$2.28\times10^{-3}\pm5.79\times10^{-4}$
SM 38:0;2O	$8.93\times10^{-5}\pm1.70\times10^{-5}$	$3.08\times10^{-4}\pm8.93\times10^{-5}$
SM 36:2;2O|SM 18:2;2O/18:0	$3.40\times10^{-5}\pm1.35\times10^{-5}$	$1.17\times10^{-4}\pm3.08\times10^{-5}$
SM 36:1;2O|SM 18:1;2O/18:0	$4.65\times10^{-4}\pm1.09\times10^{-4}$	$4.02\times10^{-3}\pm9.93\times10^{-4}$
SM 36:1;2O|SM 16:1;2O/20:0	$1.20\times10^{-3}\pm2.32\times10^{-4}$	$4.02\times10^{-3}\pm1.19\times10^{-3}$
SM 36:0;2O	$3.16\times10^{-5}\pm1.68\times10^{-5}$	$1.89\times10^{-4}\pm8.19\times10^{-5}$
SM 35:5;2O	$6.35\times10^{-5}\pm1.20\times10^{-5}$	$2.35\times10^{-5}\pm1.17\times10^{-5}$
SM 34:2;2O|SM 18:2;2O/16:0	$1.60\times10^{-4}\pm2.72\times10^{-5}$	$1.96\times10^{-4}\pm2.86\times10^{-5}$
SM 34:2;2O	$2.73\times10^{-4}\pm2.36\times10^{-5}$	$1.23\times10^{-4}\pm2.93\times10^{-5}$
SM 34:1;2O|SM 18:1;2O/16:0	$9.79\times10^{-3}\pm1.69\times10^{-3}$	$1.27\times10^{-2}\pm1.22\times10^{-3}$
SM 34:1;2O	$1.07\times10^{-4}\pm1.43\times10^{-5}$	$1.56\times10^{-4}\pm2.10\times10^{-5}$
SM 34:0;2O	$2.69\times10^{-4}\pm7.07\times10^{-5}$	$1.01\times10^{-3}\pm1.72\times10^{-4}$
SM 33:1;2O|SM 17:1;2O/16:0	$3.14\times10^{-4}\pm5.19\times10^{-5}$	$2.78\times10^{-4}\pm5.26\times10^{-5}$
SM 33:1;2O	$8.78\times10^{-6}\pm1.98\times10^{-6}$	$1.05\times10^{-5}\pm3.53\times10^{-6}$
SM 32:1;2O|SM 18:1;2O/14:0	$3.49\times10^{-5}\pm6.26\times10^{-6}$	$6.94\times10^{-5}\pm1.23\times10^{-5}$
SM 32:1;2O|SM 16:1;2O/16:0	$5.17\times10^{-5}\pm6.97\times10^{-6}$	$7.04\times10^{-5}\pm1.07\times10^{-5}$
PS 42:9	$9.69\times10^{-6}\pm4.94\times10^{-6}$	$2.86\times10^{-5}\pm1.49\times10^{-5}$
PS 40:6	$1.94\times10^{-4}\pm1.37\times10^{-4}$	$3.86\times10^{-4}\pm1.23\times10^{-4}$
PS 40:1	$1.10\times10^{-5}\pm6.54\times10^{-6}$	$6.61\times10^{-6}\pm1.59\times10^{-6}$
PS 36:2	$4.76\times10^{-5}\pm2.55\times10^{-5}$	$6.52\times10^{-5}\pm1.75\times10^{-5}$
PS 36:1	$1.82\times10^{-5}\pm8.00\times10^{-6}$	$2.40\times10^{-4}\pm7.06\times10^{-5}$
PMeOH 38:6|PMeOH 16:0_22:6	$1.47\times10^{-6}\pm7.13\times10^{-7}$	$2.41\times10^{-6}\pm1.49\times10^{-6}$
PMeOH 36:4|PMeOH 16:0_20:4	$1.61\times10^{-8}\pm1.44\times10^{-8}$	$2.35\times10^{-6}\pm1.02\times10^{-6}$

PMeOH 36:2|PMeOH 18:0_18:2	$1.31\times10^{-7}\pm4.02\times10^{-8}$	$1.88\times10^{-6}\pm1.45\times10^{-7}$
PMeOH 34:2|PMeOH 16:0_18:2	$3.11\times10^{-6}\pm2.56\times10^{-6}$	-
PI 42:9|PI 20:3_22:6	$3.63\times10^{-10}\pm7.27\times10^{-10}$	$1.07\times10^{-6}\pm6.27\times10^{-7}$
PI 42:10|PI 20:4_22:6	$2.09\times10^{-7}\pm2.10\times10^{-7}$	$4.92\times10^{-6}\pm1.96\times10^{-6}$
PI 40:8|PI 20:4_20:4	$4.31\times10^{-7}\pm1.36\times10^{-7}$	$2.98\times10^{-5}\pm1.34\times10^{-5}$
PI 40:7|PI 18:1_22:6	$3.25\times10^{-6}\pm1.53\times10^{-6}$	$1.36\times10^{-5}\pm2.21\times10^{-6}$
PI 40:6|PI 20:2_20:4	$2.00\times10^{-6}\pm5.65\times10^{-7}$	$2.89\times10^{-6}\pm7.32\times10^{-7}$
PI 40:6|PI 18:1_22:5	$1.19\times10^{-5}\pm7.40\times10^{-6}$	$7.56\times10^{-6}\pm2.25\times10^{-6}$
PI 40:6|PI 18:0_22:6	$2.80\times10^{-5}\pm2.11\times10^{-5}$	$1.45\times10^{-4}\pm4.03\times10^{-5}$
PI 40:5|PI 18:0_22:5	$9.05\times10^{-6}\pm5.81\times10^{-6}$	$4.62\times10^{-5}\pm7.33\times10^{-6}$
PI 40:5	$2.61\times10^{-4}\pm6.57\times10^{-5}$	$2.79\times10^{-4}\pm6.26\times10^{-5}$
PI 40:4|PI 20:1_20:3	$7.61\times10^{-6}\pm4.54\times10^{-6}$	$1.75\times10^{-5}\pm2.21\times10^{-6}$
PI 40:4|PI 20:0_20:4	$8.83\times10^{-5}\pm5.27\times10^{-5}$	$5.02\times10^{-6}\pm1.09\times10^{-6}$
PI 40:4|PI 18:0_22:4	$1.23\times10^{-6}\pm3.51\times10^{-7}$	$3.88\times10^{-5}\pm4.56\times10^{-6}$
PI 40:3|PI 20:0_20:3	$5.96\times10^{-6}\pm1.85\times10^{-6}$	$1.80\times10^{-7}\pm1.91\times10^{-7}$
PI 40:3|PI 18:0_22:3	$7.05\times10^{-8}\pm4.58\times10^{-8}$	$1.70\times10^{-5}\pm4.38\times10^{-6}$
PI 40:2|PI 18:0_22:2	$8.90\times10^{-7}\pm3.10\times10^{-7}$	$1.45\times10^{-6}\pm2.19\times10^{-7}$
PI 39:6	$1.65\times10^{-5}\pm7.15\times10^{-6}$	$4.70\times10^{-7}\pm6.35\times10^{-7}$
PI 39:4|PI 19:0_20:4	$6.50\times10^{-6}\pm2.03\times10^{-6}$	$1.81\times10^{-5}\pm3.31\times10^{-6}$
PI 39:4	$1.49\times10^{-4}\pm5.60\times10^{-5}$	$5.44\times10^{-6}\pm1.32\times10^{-6}$
PI 39:3|PI 19:0_20:3	$1.22\times10^{-5}\pm5.33\times10^{-6}$	$1.57\times10^{-6}\pm7.35\times10^{-8}$
PI 38:6|PI 18:2_20:4	$7.63\times10^{-6}\pm2.04\times10^{-6}$	$1.61\times10^{-5}\pm3.88\times10^{-6}$
PI 38:6|PI 16:0_22:6	$1.55\times10^{-4}\pm6.25\times10^{-5}$	$2.74\times10^{-5}\pm5.11\times10^{-6}$
PI 38:5|PI 18:2_20:3	$1.57\times10^{-5}\pm4.89\times10^{-6}$	$4.62\times10^{-6}\pm1.25\times10^{-6}$
PI 38:5|PI 18:1_20:4	$3.42\times10^{-4}\pm2.67\times10^{-4}$	$1.45\times10^{-6}\pm7.97\times10^{-7}$
PI 38:5|PI 18:0_20:5	$1.23\times10^{-4}\pm3.92\times10^{-5}$	$2.10\times10^{-5}\pm5.68\times10^{-6}$

PI 38:5|PI 16:0_22:5	$1.55×10^{-5}±8.69×10^{-6}$	$4.83×10^{-5}±1.05×10^{-5}$
PI 38:5;O|PI 18:1_20:4;O	$4.15×10^{-7}±4.03×10^{-7}$	$1.44×10^{-6}±9.76×10^{-7}$
PI 38:5;O|PI 18:0_20:5;O	$2.83×10^{-7}±1.44×10^{-7}$	$1.39×10^{-6}±9.83×10^{-7}$
PI 38:5;3O|PI 18:0_20:5;3O	$5.38×10^{-7}±4.16×10^{-7}$	$1.09×10^{-6}±2.13×10^{-7}$
PI 38:5	$1.79×10^{-4}±5.39×10^{-5}$	$2.85×10^{-4}±9.32×10^{-5}$
PI 38:4|PI 18:1_20:3	$8.08×10^{-6}±1.87×10^{-6}$	$4.13×10^{-5}±2.08×10^{-5}$
PI 38:4|PI 18:0_20:4	$1.26×10^{-4}±5.68×10^{-5}$	$3.25×10^{-3}±5.26×10^{-4}$
PI 38:4;O|PI 18:0_20:4;O	$2.73×10^{-6}±1.83×10^{-6}$	$1.28×10^{-5}±2.20×10^{-6}$
PI 38:4;3O|PI 18:0_20:4;3O	$2.41×10^{-6}±2.06×10^{-6}$	$3.59×10^{-6}±1.55×10^{-6}$
PI 38:3|PI 18:0_20:3	$8.49×10^{-7}±2.30×10^{-7}$	$2.49×10^{-4}±9.41×10^{-5}$
PI 38:3;2O|PI 18:0_20:3;2O	$5.20×10^{-7}±3.48×10^{-7}$	$4.60×10^{-7}±6.74×10^{-8}$
PI 38:3	$3.89×10^{-4}±1.84×10^{-4}$	$7.03×10^{-4}±1.22×10^{-4}$
PI 38:2|PI 18:0_20:2	$4.47×10^{-6}±2.26×10^{-6}$	$1.41×10^{-5}±4.89×10^{-6}$
PI 38:1|PI 18:0_20:1	$1.34×10^{-6}±1.91×10^{-7}$	$3.92×10^{-6}±7.38×10^{-7}$
PI 37:5|PI 17:1_20:4	$5.02×10^{-6}±1.46×10^{-6}$	$1.28×10^{-7}±8.15×10^{-8}$
PI 37:4|PI 17:0_20:4	$9.80×10^{-5}±3.53×10^{-5}$	$1.68×10^{-6}±6.12×10^{-7}$
PI 37:4	$8.68×10^{-5}±4.03×10^{-5}$	-
PI 37:3|PI 17:0_20:3	$4.43×10^{-7}±2.06×10^{-7}$	$8.50×10^{-6}±1.98×10^{-6}$
PI 37:2|PI 19:0_18:2	$5.74×10^{-6}±1.69×10^{-6}$	$7.84×10^{-7}±1.81×10^{-7}$
PI 36:5|PI 16:1_20:4	$2.14×10^{-6}±6.36×10^{-7}$	$1.71×10^{-6}±4.67×10^{-7}$
PI 36:5|PI 16:0_20:5	$1.87×10^{-6}±8.59×10^{-7}$	$3.58×10^{-6}±8.05×10^{-7}$
PI 36:4|PI 18:2_18:2	$3.25×10^{-6}±2.06×10^{-6}$	$3.89×10^{-6}±1.08×10^{-6}$
PI 36:4|PI 18:1_18:3	$6.78×10^{-6}±1.52×10^{-6}$	$3.19×10^{-6}±1.58×10^{-6}$
PI 36:4|PI 16:0_20:4	$5.06×10^{-4}±1.78×10^{-4}$	$1.93×10^{-4}±3.68×10^{-5}$
PI 36:4; O|PI 16:0_20:4; O	$1.64×10^{-6}±1.20×10^{-6}$	$1.19×10^{-6}±3.77×10^{-7}$
PI 36:4	$1.75×10^{-3}±7.36×10^{-4}$	$1.58×10^{-4}±5.32×10^{-5}$

PI 36:3|PI 18:1_18:2	$1.78\times10^{-4}\pm4.51\times10^{-5}$	$4.41\times10^{-5}\pm1.06\times10^{-5}$
PI 36:3|PI 16:0_20:3	$1.57\times10^{-5}\pm8.22\times10^{-6}$	$7.04\times10^{-5}\pm8.18\times10^{-6}$
PI 36:3; O|PI 18:1_18:2; O	$1.12\times10^{-6}\pm9.41\times10^{-7}$	$6.17\times10^{-7}\pm4.59\times10^{-7}$
PI 36:2|PI 18:1_18:1	$4.96\times10^{-5}\pm2.45\times10^{-5}$	$2.42\times10^{-4}\pm3.85\times10^{-5}$
PI 36:2|PI 18:0_18:2	$6.19\times10^{-6}\pm1.16\times10^{-6}$	$4.58\times10^{-4}\pm8.69\times10^{-5}$
PI 36:2	$5.90\times10^{-4}\pm2.37\times10^{-4}$	$4.36\times10^{-4}\pm5.43\times10^{-5}$
PI 36:1|PI 18:0_18:1	$9.90\times10^{-10}\pm1.98\times10^{-9}$	$5.52\times10^{-5}\pm2.53\times10^{-5}$
PI 36:1	$1.29\times10^{-5}\pm5.49\times10^{-6}$	$9.13\times10^{-5}\pm1.82\times10^{-5}$
PI 36:0|PI 18:0_18:0	$1.33\times10^{-5}\pm3.12\times10^{-6}$	$4.76\times10^{-5}\pm1.11\times10^{-5}$
PI 36:0	$2.17\times10^{-5}\pm8.63\times10^{-6}$	$5.91\times10^{-5}\pm2.12\times10^{-5}$
PI 35:2|PI 17:0_18:2	$2.62\times10^{-7}\pm1.13\times10^{-7}$	$6.40\times10^{-6}\pm3.00\times10^{-6}$
PI 34:3|PI 16:1_18:2	$2.18\times10^{-6}\pm6.52\times10^{-7}$	$1.69\times10^{-6}\pm3.60\times10^{-7}$
PI 34:3|PI 16:0_18:3	$4.95\times10^{-7}\pm1.93\times10^{-7}$	$4.18\times10^{-6}\pm1.37\times10^{-6}$
PI 34:2|PI 16:0_18:2	$6.74\times10^{-5}\pm3.56\times10^{-5}$	$1.34\times10^{-4}\pm3.75\times10^{-5}$
PI 34:2; O|PI 16:0_18:2; O	$6.37\times10^{-7}\pm5.20\times10^{-7}$	$1.05\times10^{-6}\pm5.06\times10^{-7}$
PI 34:1|PI 16:0_18:1	$3.62\times10^{-5}\pm1.26\times10^{-5}$	$2.62\times10^{-4}\pm3.48\times10^{-4}$
PI 34:1	$5.28\times10^{-5}\pm2.20\times10^{-5}$	$5.08\times10^{-5}\pm8.05\times10^{-6}$
PI 34:0|PI 16:0_18:0	$1.61\times10^{-6}\pm1.57\times10^{-6}$	$3.30\times10^{-6}\pm1.24\times10^{-6}$
PI 32:1|PI 16:0_16:1	$1.35\times10^{-5}\pm5.17\times10^{-6}$	$9.50\times10^{-6}\pm3.87\times10^{-6}$
PG O-36:3|PG O-18:2_18:1	$1.98\times10^{-6}\pm8.71\times10^{-7}$	$1.43\times10^{-6}\pm4.39\times10^{-7}$
PG O-34:2|PG O-16:1_18:1	$7.06\times10^{-6}\pm4.38\times10^{-6}$	$5.43\times10^{-6}\pm7.99\times10^{-7}$
PG O-34:1|PG O-16:1_18:0	$1.31\times10^{-4}\pm1.76\times10^{-5}$	$1.14\times10^{-4}\pm4.31\times10^{-5}$
PG 44:12|PG 22:6_22:6	$9.52\times10^{-5}\pm3.80\times10^{-5}$	$1.24\times10^{-5}\pm4.31\times10^{-6}$
PG 44:11|PG 22:5_22:6	$1.07\times10^{-6}\pm4.89\times10^{-7}$	$1.68\times10^{-5}\pm3.39\times10^{-6}$
PG 44:10|PG 22:5_22:5	$3.74\times10^{-6}\pm6.03\times10^{-7}$	$2.77\times10^{-6}\pm1.29\times10^{-6}$
PG 42:8|PG 20:3_22:5	$4.70\times10^{-6}\pm1.20\times10^{-6}$	$4.78\times10^{-6}\pm7.04\times10^{-7}$

PG 42:11|PG 20:5_22:6	$4.27\times10^{-6}\pm1.53\times10^{-6}$	$1.32\times10^{-7}\pm1.21\times10^{-7}$
PG 42:10|PG 20:4_22:6	$8.93\times10^{-7}\pm3.04\times10^{-7}$	$1.19\times10^{-5}\pm2.81\times10^{-6}$
PG 40:8|PG 18:2_22:6	$1.86\times10^{-5}\pm4.86\times10^{-6}$	$9.95\times10^{-6}\pm3.08\times10^{-6}$
PG 40:7|PG 18:1_22:6	$1.72\times10^{-5}\pm4.01\times10^{-6}$	$2.49\times10^{-5}\pm3.81\times10^{-6}$
PG 40:6|PG 18:2_22:4	$1.18\times10^{-5}\pm2.17\times10^{-6}$	$5.75\times10^{-6}\pm8.28\times10^{-7}$
PG 40:6|PG 18:1_22:5	$4.44\times10^{-6}\pm2.01\times10^{-6}$	$1.26\times10^{-5}\pm2.07\times10^{-6}$
PG 40:5|PG 18:1_22:4	$1.76\times10^{-6}\pm3.62\times10^{-7}$	$9.26\times10^{-6}\pm1.63\times10^{-6}$
PG 38:7|PG 16:1_22:6	$3.42\times10^{-6}\pm9.85\times10^{-7}$	$1.27\times10^{-6}\pm3.87\times10^{-7}$
PG 38:6|PG 18:2_20:4	$4.58\times10^{-6}\pm1.94\times10^{-6}$	$1.78\times10^{-5}\pm4.82\times10^{-6}$
PG 38:6|PG 16:0_22:6	$3.05\times10^{-5}\pm1.88\times10^{-5}$	$8.65\times10^{-6}\pm2.95\times10^{-6}$
PG 38:3|PG 20:1_18:2	$3.55\times10^{-7}\pm1.85\times10^{-7}$	$2.95\times10^{-6}\pm7.32\times10^{-7}$
PG 38:3|PG 18:1_20:2	$6.48\times10^{-7}\pm5.76\times10^{-8}$	$2.25\times10^{-6}\pm4.63\times10^{-7}$
PG 38:2|PG 18:0_20:2	$5.34\times10^{-9}\pm6.96\times10^{-9}$	$1.20\times10^{-6}\pm5.36\times10^{-7}$
PG 36:4|PG 18:2_18:2	$1.04\times10^{-3}\pm1.88\times10^{-4}$	$2.68\times10^{-4}\pm9.51\times10^{-5}$
PG 36:4|PG 16:0_20:4	$2.76\times10^{-5}\pm6.87\times10^{-6}$	$4.75\times10^{-6}\pm2.31\times10^{-6}$
PG 36:3|PG 18:1_18:2	$9.34\times10^{-6}\pm2.80\times10^{-6}$	$3.15\times10^{-5}\pm8.62\times10^{-6}$
PG 36:2|PG 18:1_18:1	$7.68\times10^{-6}\pm1.25\times10^{-6}$	$3.54\times10^{-5}\pm5.34\times10^{-6}$
PG 36:2|PG 18:0_18:2	$9.50\times10^{-7}\pm3.56\times10^{-7}$	$1.33\times10^{-4}\pm1.56\times10^{-5}$
PG 36:1|PG 18:0_18:1	$9.79\times10^{-8}\pm1.07\times10^{-7}$	$1.19\times10^{-5}\pm1.47\times10^{-6}$
PG 35:2|PG 17:0_18:2	$2.19\times10^{-6}\pm8.58\times10^{-7}$	$2.66\times10^{-7}\pm2.28\times10^{-7}$
PG 35:1|PG 17:0_18:1	$7.20\times10^{-6}\pm1.59\times10^{-6}$	-
PG 35:1|PG 16:0_19:1	$1.41\times10^{-8}\pm2.83\times10^{-8}$	$8.50\times10^{-6}\pm2.01\times10^{-6}$
PG 34:2|PG 16:0_18:2	$1.10\times10^{-4}\pm5.09\times10^{-5}$	$4.73\times10^{-6}\pm1.99\times10^{-6}$
PG 34:1|PG 16:0_18:1	$2.80\times10^{-5}\pm1.68\times10^{-5}$	$1.58\times10^{-4}\pm1.37\times10^{-5}$
PG 32:1|PG 16:0_16:1	$4.64\times10^{-6}\pm2.82\times10^{-6}$	$1.55\times10^{-7}\pm7.24\times10^{-8}$
PG 32:0|PG 16:0_16:0	$6.68\times10^{-6}\pm4.33\times10^{-6}$	$7.93\times10^{-7}\pm2.84\times10^{-7}$

PE O-42:7|PE O-20:1_22:6	$6.41\times10^{-6}\pm3.15\times10^{-6}$	$8.59\times10^{-6}\pm1.38\times10^{-6}$
PE O-42:6|PE O-20:0_22:6	$9.08\times10^{-6}\pm3.33\times10^{-6}$	$9.81\times10^{-6}\pm3.50\times10^{-6}$
PE O-40:6|PE O-18:0_22:6	$1.82\times10^{-5}\pm5.31\times10^{-6}$	$2.25\times10^{-5}\pm3.14\times10^{-6}$
PE O-40:5|PE O-20:1_20:4	$2.24\times10^{-5}\pm4.86\times10^{-6}$	$3.82\times10^{-5}\pm8.45\times10^{-6}$
PE O-40:4|PE O-18:0_22:4	$5.81\times10^{-6}\pm1.51\times10^{-6}$	$9.64\times10^{-6}\pm1.82\times10^{-6}$
PE O-38:7|PE O-16:1_22:6	$1.20\times10^{-6}\pm3.84\times10^{-7}$	$2.24\times10^{-4}\pm4.56\times10^{-5}$
PE O-38:6|PE O-16:1_22:5	$4.23\times10^{-5}\pm4.05\times10^{-6}$	$1.46\times10^{-4}\pm3.05\times10^{-5}$
PE O-38:2|PE O-18:1_20:1	$6.75\times10^{-7}\pm3.71\times10^{-7}$	$3.66\times10^{-6}\pm5.13\times10^{-7}$
PE O-36:5|PE O-16:1_20:4	$1.58\times10^{-6}\pm6.87\times10^{-7}$	$1.11\times10^{-4}\pm7.10\times10^{-5}$
PE O-36:4|PE O-16:1_20:3	$5.10\times10^{-6}\pm2.58\times10^{-6}$	$1.91\times10^{-6}\pm6.29\times10^{-7}$
PE O-36:3|PE O-18:2_18:1	$9.45\times10^{-6}\pm1.20\times10^{-6}$	$3.94\times10^{-6}\pm1.18\times10^{-6}$
PE O-34:3|PE O-16:1_18:2	$3.02\times10^{-5}\pm1.12\times10^{-5}$	$2.23\times10^{-7}\pm6.88\times10^{-8}$
PE O-20:5	$7.26\times10^{-5}\pm1.41\times10^{-5}$	$2.10\times10^{-5}\pm3.87\times10^{-6}$
PE O-20:4	$1.93\times10^{-3}\pm3.73\times10^{-4}$	$1.48\times10^{-3}\pm2.72\times10^{-4}$
PE O-18:0	$5.38\times10^{-5}\pm1.33\times10^{-5}$	$3.79\times10^{-5}\pm8.80\times10^{-6}$
PE 44:12|PE 22:6_22:6	$1.45\times10^{-6}\pm9.69\times10^{-7}$	$1.17\times10^{-4}\pm4.20\times10^{-5}$
PE 42:9	$4.30\times10^{-5}\pm9.30\times10^{-6}$	$9.17\times10^{-5}\pm9.24\times10^{-6}$
PE 42:8|PE 20:3_22:5	$2.22\times10^{-6}\pm9.39\times10^{-7}$	$3.53\times10^{-6}\pm7.13\times10^{-7}$
PE 42:8|PE 20:2_22:6	$2.45\times10^{-7}\pm9.67\times10^{-8}$	$6.12\times10^{-6}\pm2.74\times10^{-7}$
PE 42:8	$2.28\times10^{-5}\pm9.08\times10^{-6}$	$5.76\times10^{-5}\pm2.09\times10^{-5}$
PE 42:6|PE 20:0_22:6	$7.30\times10^{-7}\pm5.98\times10^{-7}$	$6.30\times10^{-6}\pm3.54\times10^{-6}$
PE 42:6	$2.92\times10^{-4}\pm1.34\times10^{-4}$	$5.36\times10^{-6}\pm4.52\times10^{-6}$
PE 41:6|PE 19:0_22:6	$2.27\times10^{-5}\pm1.47\times10^{-5}$	-
PE 40:8|PE 18:2_22:6	$1.38\times10^{-4}\pm3.60\times10^{-5}$	$2.12\times10^{-4}\pm3.99\times10^{-5}$
PE 40:7|PE 18:1_22:6	$4.31\times10^{-6}\pm9.63\times10^{-7}$	$3.81\times10^{-4}\pm1.72\times10^{-4}$
PE 40:6|PE 18:1_22:5	$8.18\times10^{-6}\pm2.67\times10^{-6}$	$3.25\times10^{-6}\pm6.54\times10^{-7}$

PE 40:6|PE 18:0_22:6	$2.40\times10^{-5}\pm8.87\times10^{-6}$	$2.41\times10^{-3}\pm4.92\times10^{-4}$
PE 40:5|PE 18:0_22:5	$2.30\times10^{-6}\pm1.90\times10^{-6}$	$5.36\times10^{-5}\pm7.47\times10^{-6}$
PE 39:6|PE 17:0_22:6	$1.18\times10^{-6}\pm5.22\times10^{-7}$	$3.52\times10^{-5}\pm6.57\times10^{-6}$
PE 39:4|PE 19:0_20:4	$1.81\times10^{-6}\pm1.03\times10^{-6}$	$6.96\times10^{-6}\pm1.15\times10^{-6}$
PE 38:7|PE 18:3_20:4	$4.00\times10^{-6}\pm1.53\times10^{-6}$	$3.10\times10^{-6}\pm6.15\times10^{-7}$
PE 38:7|PE 18:2_20:5	$5.89\times10^{-6}\pm1.93\times10^{-6}$	$1.15\times10^{-5}\pm9.47\times10^{-6}$
PE 38:7|PE 16:1_22:6	$1.26\times10^{-5}\pm2.37\times10^{-6}$	$3.88\times10^{-5}\pm8.89\times10^{-6}$
PE 38:6|PE 18:2_20:4	$3.29\times10^{-5}\pm9.22\times10^{-6}$	$1.32\times10^{-4}\pm1.87\times10^{-5}$
PE 38:6|PE 16:0_22:6	$3.29\times10^{-3}\pm1.16\times10^{-3}$	$1.75\times10^{-4}\pm3.75\times10^{-5}$
PE 38:6; O|PE 16:0_22:6; O	$1.71\times10^{-6}\pm1.09\times10^{-6}$	$3.84\times10^{-6}\pm1.02\times10^{-6}$
PE 38:6	$3.73\times10^{-5}\pm1.75\times10^{-5}$	$3.07\times10^{-4}\pm1.76\times10^{-4}$
PE 38:5|PE 18:1_20:4	$7.12\times10^{-5}\pm2.03\times10^{-5}$	$7.27\times10^{-4}\pm1.45\times10^{-4}$
PE 38:5|PE 16:0_22:5	$1.16\times10^{-5}\pm5.23\times10^{-6}$	$1.33\times10^{-4}\pm2.40\times10^{-5}$
PE 38:5; O|PE 18:1_20:4; O	$3.77\times10^{-7}\pm1.92\times10^{-7}$	$1.62\times10^{-6}\pm6.48\times10^{-7}$
PE 38:5;2O|PE 18:1_20:4;2O	$1.03\times10^{-6}\pm1.06\times10^{-6}$	$1.43\times10^{-6}\pm6.17\times10^{-7}$
PE 38:4|PE 18:1_20:3	$5.45\times10^{-6}\pm1.90\times10^{-6}$	$3.22\times10^{-5}\pm4.39\times10^{-6}$
PE 38:4|PE 18:0_20:4	$1.89\times10^{-5}\pm5.36\times10^{-6}$	$2.50\times10^{-3}\pm5.62\times10^{-4}$
PE 38:4	$3.14\times10^{-4}\pm6.13\times10^{-5}$	$1.35\times10^{-4}\pm3.18\times10^{-5}$
PE 38:3|PE 18:0_20:3	$2.03\times10^{-4}\pm6.28\times10^{-5}$	$1.12\times10^{-3}\pm2.56\times10^{-4}$
PE 38:2|PE 18:1_20:1	$5.24\times10^{-5}\pm2.12\times10^{-5}$	$6.01\times10^{-5}\pm3.54\times10^{-6}$
PE 38:1|PE 18:0_20:1	$3.14\times10^{-5}\pm7.23\times10^{-6}$	$2.82\times10^{-5}\pm8.25\times10^{-6}$
PE 37:3|PE 17:0_20:3	$1.28\times10^{-6}\pm4.92\times10^{-7}$	$2.31\times10^{-6}\pm3.25\times10^{-7}$
PE 37:2|PE 19:0_18:2	$2.17\times10^{-5}\pm5.56\times10^{-6}$	$1.84\times10^{-6}\pm4.69\times10^{-7}$
PE 36:6|PE 14:0_22:6	$1.52\times10^{-7}\pm9.26\times10^{-8}$	$3.30\times10^{-6}\pm1.09\times10^{-6}$
PE 36:5|PE 18:2_18:3	$5.26\times10^{-6}\pm4.59\times10^{-6}$	$1.04\times10^{-6}\pm1.63\times10^{-6}$
PE 36:5|PE 16:1_20:4	$3.73\times10^{-5}\pm1.27\times10^{-5}$	$7.59\times10^{-6}\pm1.24\times10^{-6}$

PE 36:5|PE 16:0_20:5	$1.21\times10^{-4}\pm3.75\times10^{-5}$	$6.43\times10^{-6}\pm9.21\times10^{-7}$
PE 36:4|PE 18:2_18:2	$1.79\times10^{-4}\pm2.08\times10^{-5}$	$3.07\times10^{-4}\pm6.88\times10^{-5}$
PE 36:4|PE 16:0_20:4	$1.88\times10^{-4}\pm7.88\times10^{-5}$	$6.38\times10^{-4}\pm1.35\times10^{-4}$
PE 36:3|PE 18:1_18:2	$5.33\times10^{-4}\pm1.26\times10^{-4}$	$1.40\times10^{-3}\pm1.82\times10^{-4}$
PE 36:3|PE 16:0_20:3	$3.65\times10^{-5}\pm2.02\times10^{-5}$	$8.10\times10^{-5}\pm1.35\times10^{-5}$
PE 36:2|PE 18:1_18:1	$3.19\times10^{-6}\pm6.38\times10^{-6}$	$9.82\times10^{-5}\pm4.04\times10^{-5}$
PE 36:2|PE 18:0_18:2	$3.94\times10^{-3}\pm1.25\times10^{-3}$	$4.05\times10^{-3}\pm1.39\times10^{-3}$
PE 36:1|PE 18:0_18:1	$7.39\times10^{-4}\pm7.01\times10^{-5}$	$1.68\times10^{-3}\pm3.81\times10^{-4}$
PE 36:0|PE 18:0_18:0	$1.80\times10^{-5}\pm5.14\times10^{-6}$	$5.72\times10^{-5}\pm1.44\times10^{-5}$
PE 35:1|PE 17:0_18:1	$1.05\times10^{-6}\pm9.23\times10^{-7}$	$1.22\times10^{-5}\pm1.40\times10^{-6}$
PE 34:3|PE 16:1_18:2	$2.12\times10^{-4}\pm4.40\times10^{-5}$	$4.72\times10^{-5}\pm1.05\times10^{-5}$
PE 34:3|PE 16:0_18:3	$7.21\times10^{-5}\pm3.22\times10^{-5}$	$6.60\times10^{-6}\pm3.33\times10^{-6}$
PE 34:2|PE 16:0_18:2	$7.99\times10^{-5}\pm3.70\times10^{-5}$	$3.06\times10^{-4}\pm4.79\times10^{-5}$
PE 34:2;O|PE 16:0_18:2;O	$8.38\times10^{-7}\pm4.05\times10^{-7}$	$4.68\times10^{-7}\pm2.53\times10^{-7}$
PE 34:2;2O|PE 16:0_18:2;2O	$3.86\times10^{-7}\pm2.81\times10^{-7}$	$4.65\times10^{-7}\pm1.11\times10^{-7}$
PE 34:1|PE 16:0_18:1	$2.83\times10^{-4}\pm1.13\times10^{-4}$	$1.24\times10^{-3}\pm1.22\times10^{-4}$
PE 34:0|PE 16:0_18:0	$2.70\times10^{-4}\pm1.28\times10^{-4}$	$4.87\times10^{-4}\pm1.19\times10^{-4}$
PE 32:1|PE 16:0_16:1	$7.37\times10^{-6}\pm2.37\times10^{-6}$	$6.13\times10^{-6}\pm1.63\times10^{-6}$
PE 32:0|PE 16:0_16:0	$1.72\times10^{-4}\pm8.78\times10^{-5}$	$2.38\times10^{-5}\pm2.38\times10^{-5}$
PC O-47:6	$2.25\times10^{-7}\pm4.51\times10^{-7}$	$6.30\times10^{-7}\pm7.40\times10^{-7}$
PC O-44:7	$1.09\times10^{-5}\pm6.29\times10^{-6}$	$1.97\times10^{-5}\pm2.55\times10^{-6}$
PC O-44:6	$1.03\times10^{-5}\pm3.03\times10^{-6}$	$1.61\times10^{-5}\pm4.61\times10^{-6}$
PC O-44:5	$6.91\times10^{-6}\pm2.25\times10^{-6}$	$1.20\times10^{-5}\pm1.04\times10^{-6}$
PC O-44:12	$1.21\times10^{-5}\pm6.95\times10^{-6}$	$3.29\times10^{-5}\pm6.33\times10^{-6}$
PC O-42:9	$5.08\times10^{-6}\pm2.19\times10^{-6}$	$3.74\times10^{-5}\pm1.63\times10^{-5}$
PC O-42:7	$2.08\times10^{-5}\pm5.38\times10^{-6}$	$5.07\times10^{-5}\pm8.51\times10^{-6}$

PC O-42:6	$8.35\times10^{-5}\pm2.14\times10^{-5}$	$2.14\times10^{-4}\pm2.07\times10^{-5}$
PC O-42:5	$3.24\times10^{-5}\pm1.38\times10^{-5}$	$4.84\times10^{-5}\pm7.49\times10^{-6}$
PC O-42:4	$1.50\times10^{-5}\pm1.85\times10^{-6}$	$1.64\times10^{-5}\pm3.48\times10^{-6}$
PC O-40:7	$9.84\times10^{-5}\pm2.13\times10^{-5}$	$5.08\times10^{-4}\pm1.42\times10^{-4}$
PC O-40:6	$6.28\times10^{-5}\pm1.82\times10^{-5}$	$3.93\times10^{-4}\pm8.06\times10^{-5}$
PC O-40:5	$2.13\times10^{-5}\pm5.22\times10^{-6}$	$3.67\times10^{-5}\pm7.98\times10^{-6}$
PC O-40:4	$7.78\times10^{-5}\pm2.25\times10^{-5}$	$1.43\times10^{-4}\pm3.96\times10^{-5}$
PC O-40:3	$1.43\times10^{-5}\pm4.92\times10^{-6}$	$2.27\times10^{-5}\pm4.39\times10^{-6}$
PC O-39:7	$3.88\times10^{-5}\pm1.22\times10^{-5}$	$4.28\times10^{-5}\pm2.04\times10^{-5}$
PC O-39:6	$2.46\times10^{-5}\pm2.92\times10^{-6}$	$4.40\times10^{-5}\pm8.20\times10^{-6}$
PC O-38:7	$1.69\times10^{-4}\pm1.88\times10^{-5}$	$1.01\times10^{-4}\pm3.39\times10^{-5}$
PC O-38:6	$2.52\times10^{-4}\pm6.91\times10^{-5}$	$6.54\times10^{-4}\pm1.10\times10^{-4}$
PC O-38:5	$3.47\times10^{-4}\pm1.63\times10^{-4}$	$1.75\times10^{-3}\pm2.52\times10^{-4}$
PC O-38:3	$1.51\times10^{-5}\pm3.07\times10^{-6}$	$4.79\times10^{-5}\pm7.36\times10^{-6}$
PC O-37:7	$7.01\times10^{-5}\pm1.30\times10^{-5}$	$2.74\times10^{-4}\pm8.09\times10^{-5}$
PC O-36:5	$7.24\times10^{-4}\pm1.08\times10^{-4}$	$1.26\times10^{-4}\pm2.36\times10^{-5}$
PC O-36:4	$9.32\times10^{-4}\pm2.01\times10^{-4}$	$1.41\times10^{-3}\pm3.30\times10^{-4}$
PC O-36:3	$1.78\times10^{-4}\pm1.69\times10^{-5}$	$4.86\times10^{-5}\pm1.13\times10^{-5}$
PC O-36:2	$5.87\times10^{-5}\pm6.78\times10^{-6}$	$1.60\times10^{-4}\pm3.23\times10^{-5}$
PC O-36:0	$4.05\times10^{-5}\pm1.21\times10^{-5}$	$4.70\times10^{-5}\pm9.83\times10^{-6}$
PC O-35:7	$1.41\times10^{-4}\pm1.74\times10^{-5}$	$4.17\times10^{-4}\pm5.10\times10^{-5}$
PC O-34:3	$1.82\times10^{-4}\pm2.44\times10^{-5}$	$1.89\times10^{-5}\pm5.66\times10^{-6}$
PC O-34:2	$6.25\times10^{-4}\pm1.10\times10^{-4}$	$1.11\times10^{-4}\pm1.58\times10^{-5}$
PC O-34:1	$5.43\times10^{-4}\pm9.05\times10^{-5}$	$2.29\times10^{-3}\pm3.49\times10^{-4}$
PC O-34:0	$4.87\times10^{-4}\pm9.74\times10^{-5}$	$6.75\times10^{-4}\pm1.60\times10^{-4}$
PC O-33:6	$8.00\times10^{-6}\pm8.87\times10^{-6}$	$1.00\times10^{-5}\pm3.25\times10^{-6}$

PC O-33:2	$1.05\times10^{-5}\pm3.99\times10^{-6}$	$2.80\times10^{-6}\pm2.08\times10^{-6}$
PC O-32:2	$1.99\times10^{-5}\pm3.53\times10^{-6}$	$1.67\times10^{-5}\pm1.61\times10^{-6}$
PC O-32:1	$4.62\times10^{-4}\pm1.44\times10^{-4}$	$1.72\times10^{-4}\pm5.35\times10^{-5}$
PC O-32:0	$3.31\times10^{-4}\pm8.54\times10^{-5}$	$2.22\times10^{-3}\pm5.81\times10^{-4}$
PC O-31:0	$7.27\times10^{-5}\pm6.44\times10^{-6}$	$3.19\times10^{-6}\pm1.81\times10^{-6}$
PC O-30:0	$1.52\times10^{-4}\pm1.64\times10^{-5}$	$2.79\times10^{-5}\pm7.75\times10^{-6}$
PC 46:12	$4.10\times10^{-5}\pm1.87\times10^{-5}$	$4.30\times10^{-5}\pm7.70\times10^{-6}$
PC 44:7	$4.99\times10^{-6}\pm2.53\times10^{-6}$	$1.17\times10^{-5}\pm6.41\times10^{-6}$
PC 44:6	$2.05\times10^{-5}\pm1.60\times10^{-5}$	$2.13\times10^{-7}\pm4.25\times10^{-7}$
PC 44:12|PC 22:6_22:6	$1.87\times10^{-3}\pm7.98\times10^{-4}$	$2.32\times10^{-3}\pm5.66\times10^{-4}$
PC 44:11	$7.52\times10^{-5}\pm3.72\times10^{-5}$	$8.15\times10^{-4}\pm2.59\times10^{-4}$
PC 44:10	$6.19\times10^{-5}\pm2.34\times10^{-5}$	$1.32\times10^{-4}\pm2.85\times10^{-5}$
PC 42:9	$9.83\times10^{-4}\pm3.68\times10^{-4}$	$1.96\times10^{-3}\pm2.53\times10^{-4}$
PC 42:8	$1.31\times10^{-4}\pm1.75\times10^{-5}$	$5.03\times10^{-4}\pm1.05\times10^{-4}$
PC 42:7	$5.66\times10^{-5}\pm2.34\times10^{-5}$	$1.67\times10^{-4}\pm2.34\times10^{-5}$
PC 42:6	$7.67\times10^{-4}\pm3.20\times10^{-4}$	$3.37\times10^{-5}\pm1.96\times10^{-5}$
PC 42:5	$7.11\times10^{-5}\pm2.81\times10^{-5}$	$2.53\times10^{-5}\pm1.16\times10^{-5}$
PC 42:4	$4.86\times10^{-5}\pm9.74\times10^{-6}$	$2.85\times10^{-5}\pm1.80\times10^{-5}$
PC 42:3	$3.00\times10^{-5}\pm7.53\times10^{-6}$	$1.99\times10^{-5}\pm6.81\times10^{-6}$
PC 42:2	$3.98\times10^{-5}\pm7.80\times10^{-6}$	$1.27\times10^{-5}\pm5.41\times10^{-6}$
PC 42:11	$1.51\times10^{-4}\pm5.67\times10^{-5}$	$1.85\times10^{-5}\pm4.32\times10^{-6}$
PC 42:10	$2.90\times10^{-3}\pm9.04\times10^{-4}$	$8.39\times10^{-3}\pm1.10\times10^{-3}$
PC 42:1	$2.90\times10^{-5}\pm3.36\times10^{-6}$	$1.19\times10^{-5}\pm4.27\times10^{-6}$
PC 41:7	$1.85\times10^{-5}\pm4.62\times10^{-6}$	$3.29\times10^{-5}\pm1.02\times10^{-5}$
PC 41:6	$5.73\times10^{-4}\pm1.73\times10^{-4}$	$5.61\times10^{-5}\pm1.37\times10^{-5}$
PC 41:5	$3.97\times10^{-6}\pm8.75\times10^{-7}$	$1.22\times10^{-5}\pm3.95\times10^{-6}$

PC 40:9	$3.89\times10^{-4}\pm1.13\times10^{-4}$	$5.69\times10^{-5}\pm9.47\times10^{-6}$
PC 40:8	$1.37\times10^{-2}\pm1.98\times10^{-3}$	$1.27\times10^{-2}\pm2.26\times10^{-3}$
PC 40:7	$4.11\times10^{-3}\pm7.31\times10^{-4}$	$1.12\times10^{-2}\pm2.88\times10^{-3}$
PC 40:6	$5.43\times10^{-3}\pm1.93\times10^{-3}$	$2.97\times10^{-2}\pm6.42\times10^{-3}$
PC 40:5	$2.62\times10^{-4}\pm7.78\times10^{-5}$	$2.39\times10^{-3}\pm8.24\times10^{-4}$
PC 40:4	$8.11\times10^{-4}\pm3.57\times10^{-4}$	$1.12\times10^{-4}\pm7.26\times10^{-5}$
PC 40:3	$3.01\times10^{-4}\pm8.22\times10^{-5}$	$1.77\times10^{-4}\pm7.07\times10^{-5}$
PC 40:2	$1.45\times10^{-4}\pm3.56\times10^{-5}$	$7.84\times10^{-5}\pm2.31\times10^{-5}$
PC 40:10	$9.83\times10^{-6}\pm3.56\times10^{-6}$	$2.26\times10^{-5}\pm5.72\times10^{-6}$
PC 40:1	$7.89\times10^{-5}\pm1.17\times10^{-5}$	$3.34\times10^{-5}\pm7.50\times10^{-6}$
PC 39:8	$9.96\times10^{-6}\pm2.52\times10^{-6}$	$2.78\times10^{-5}\pm4.15\times10^{-6}$
PC 39:7	$3.88\times10^{-5}\pm7.17\times10^{-6}$	$5.79\times10^{-5}\pm1.66\times10^{-5}$
PC 39:6	$3.37\times10^{-4}\pm1.29\times10^{-4}$	$1.46\times10^{-3}\pm3.85\times10^{-4}$
PC 39:5	$4.11\times10^{-5}\pm1.99\times10^{-5}$	$1.29\times10^{-5}\pm2.04\times10^{-5}$
PC 39:4	$2.28\times10^{-4}\pm9.08\times10^{-5}$	$2.71\times10^{-4}\pm9.10\times10^{-5}$
PC 39:3	$1.08\times10^{-4}\pm2.23\times10^{-5}$	$7.07\times10^{-5}\pm8.49\times10^{-6}$
PC 39:1	$2.55\times10^{-5}\pm5.14\times10^{-6}$	$1.76\times10^{-6}\pm2.60\times10^{-6}$
PC 38:7	$2.56\times10^{-4}\pm9.48\times10^{-5}$	$4.04\times10^{-4}\pm1.60\times10^{-4}$
PC 38:6	$2.00\times10^{-2}\pm6.14\times10^{-3}$	$6.61\times10^{-2}\pm1.56\times10^{-2}$
PC 38:5	$6.08\times10^{-3}\pm9.95\times10^{-4}$	$1.06\times10^{-2}\pm2.94\times10^{-3}$
PC 38:4	$1.16\times10^{-2}\pm1.77\times10^{-3}$	$2.98\times10^{-2}\pm1.05\times10^{-2}$
PC 38:3	$2.53\times10^{-3}\pm6.36\times10^{-4}$	$1.45\times10^{-2}\pm4.39\times10^{-3}$
PC 38:2	$3.19\times10^{-3}\pm6.53\times10^{-4}$	$3.87\times10^{-3}\pm1.23\times10^{-3}$
PC 38:1	$6.03\times10^{-4}\pm1.93\times10^{-4}$	$8.63\times10^{-4}\pm3.09\times10^{-4}$
PC 37:6	$1.75\times10^{-4}\pm7.59\times10^{-5}$	$2.16\times10^{-4}\pm8.94\times10^{-5}$
PC 37:5	$1.38\times10^{-4}\pm2.90\times10^{-5}$	$1.08\times10^{-4}\pm3.67\times10^{-5}$

PC 37:4	$5.90\times10^{-4}\pm1.16\times10^{-4}$	$1.55\times10^{-3}\pm6.11\times10^{-4}$
PC 37:3	$1.02\times10^{-4}\pm1.79\times10^{-5}$	$2.34\times10^{-4}\pm8.74\times10^{-5}$
PC 37:1	$4.03\times10^{-4}\pm1.39\times10^{-4}$	$2.39\times10^{-4}\pm7.29\times10^{-5}$
PC 37:0	$2.82\times10^{-5}\pm5.91\times10^{-6}$	$1.96\times10^{-5}\pm7.75\times10^{-6}$
PC 36:6	$3.46\times10^{-5}\pm1.08\times10^{-5}$	$6.51\times10^{-5}\pm1.53\times10^{-5}$
PC 36:5	$3.09\times10^{-3}\pm1.05\times10^{-3}$	$5.15\times10^{-4}\pm1.65\times10^{-4}$
PC 36:4	$3.30\times10^{-2}\pm8.61\times10^{-3}$	$3.15\times10^{-2}\pm1.00\times10^{-2}$
PC 36:3	$1.05\times10^{-2}\pm3.38\times10^{-3}$	$1.70\times10^{-2}\pm4.60\times10^{-3}$
PC 36:2;2O|PC 18:0_18:2;2O	$7.76\times10^{-7}\pm7.73\times10^{-7}$	$2.27\times10^{-6}\pm7.00\times10^{-7}$
PC 36:2	$1.87\times10^{-2}\pm9.38\times10^{-4}$	$5.04\times10^{-2}\pm1.20\times10^{-2}$
PC 36:1	$8.07\times10^{-3}\pm2.19\times10^{-3}$	$1.85\times10^{-2}\pm3.00\times10^{-3}$
PC 36:0|PC 18:0_18:0	$8.35\times10^{-4}\pm2.49\times10^{-4}$	$1.16\times10^{-3}\pm4.02\times10^{-4}$
PC 35:4	$2.84\times10^{-4}\pm8.03\times10^{-5}$	$2.37\times10^{-4}\pm1.09\times10^{-4}$
PC 35:3	$3.31\times10^{-4}\pm3.90\times10^{-5}$	$8.10\times10^{-5}\pm1.09\times10^{-5}$
PC 35:2	$6.43\times10^{-4}\pm1.41\times10^{-4}$	$2.07\times10^{-3}\pm6.41\times10^{-4}$
PC 35:1	$4.69\times10^{-4}\pm1.38\times10^{-4}$	$1.97\times10^{-3}\pm2.58\times10^{-4}$
PC 35:0	$3.23\times10^{-4}\pm8.06\times10^{-5}$	$2.27\times10^{-4}\pm5.22\times10^{-5}$
PC 34:4	$5.51\times10^{-5}\pm1.60\times10^{-5}$	$1.21\times10^{-4}\pm3.41\times10^{-5}$
PC 34:3	$7.00\times10^{-3}\pm1.42\times10^{-3}$	$1.04\times10^{-3}\pm2.51\times10^{-4}$
PC 34:2;2O|PC 16:0_18:2;2O	$3.51\times10^{-6}\pm3.15\times10^{-6}$	$1.45\times10^{-6}\pm3.46\times10^{-7}$
PC 34:2	$3.23\times10^{-2}\pm9.50\times10^{-3}$	$7.44\times10^{-2}\pm1.51\times10^{-2}$
PC 34:1	$3.79\times10^{-3}\pm1.18\times10^{-3}$	$5.22\times10^{-2}\pm1.28\times10^{-2}$
PC 34:0	$2.30\times10^{-3}\pm3.00\times10^{-4}$	$1.07\times10^{-2}\pm1.93\times10^{-3}$
PC 33:2	$1.03\times10^{-3}\pm3.80\times10^{-4}$	$7.03\times10^{-5}\pm5.97\times10^{-5}$
PC 33:1	$8.26\times10^{-4}\pm2.54\times10^{-4}$	$1.20\times10^{-3}\pm3.75\times10^{-4}$
PC 33:0	$3.29\times10^{-4}\pm5.25\times10^{-5}$	$6.53\times10^{-4}\pm8.68\times10^{-5}$

PC 32:2	$2.83×10^{-4}±1.16×10^{-4}$	$4.71×10^{-4}±1.96×10^{-4}$
PC 32:1	$9.66×10^{-3}±2.22×10^{-3}$	$1.36×10^{-3}±3.23×10^{-4}$
PC 32:0|PC 16:0_16:0	$1.07×10^{-2}±1.97×10^{-3}$	$3.35×10^{-2}±2.30×10^{-3}$
PC 31:1	$5.81×10^{-5}±2.00×10^{-5}$	$5.98×10^{-7}±1.20×10^{-6}$
PC 31:0	$1.20×10^{-3}±1.09×10^{-4}$	$7.44×10^{-6}±1.07×10^{-5}$
PC 30:0	$6.74×10^{-4}±1.89×10^{-4}$	$2.05×10^{-4}±6.24×10^{-5}$
PC 28:0	$1.12×10^{-6}±8.51×10^{-7}$	$3.03×10^{-6}±1.07×10^{-6}$
PA 38:6|PA 16:0_22:6	$3.07×10^{-7}±1.16×10^{-7}$	$1.12×10^{-5}±2.80×10^{-6}$
PA 36:4|PA 18:2_18:2	$2.87×10^{-7}±1.40×10^{-7}$	$4.52×10^{-6}±3.43×10^{-7}$
PA 36:4|PA 16:0_20:4	$4.77×10^{-6}±1.75×10^{-6}$	-
PA 34:2|PA 16:0_18:2	$1.43×10^{-5}±4.56×10^{-6}$	$6.70×10^{-7}±1.23×10^{-6}$
Phenylalanine	$1.31×10^{-3}±3.24×10^{-4}$	$1.85×10^{-3}±2.55×10^{-4}$
Dodecylbenzenesulfonic acid	$7.69×10^{-5}±4.16×10^{-5}$	$7.84×10^{-5}±1.32×10^{-5}$
Diisodecyl phthalate (also known as the production of plastic)	$6.58×10^{-6}±2.08×10^{-6}$	$1.83×10^{-5}±3.44×10^{-6}$
Carnitine	$1.73×10^{-3}±3.68×10^{-4}$	$2.12×10^{-3}±8.33×10^{-4}$
PA 32:0|PA 16:0_16:0	$3.10×10^{-7}±5.84×10^{-7}$	$2.39×10^{-6}±2.01×10^{-6}$
LPS 19:0	$4.40×10^{-5}±8.91×10^{-6}$	$1.26×10^{-5}±3.40×10^{-6}$
LPS 18:0	$2.83×10^{-4}±3.30×10^{-5}$	$3.26×10^{-4}±6.25×10^{-5}$
LPS 16:0	$3.01×10^{-5}±1.12×10^{-5}$	$2.06×10^{-5}±4.36×10^{-6}$
LPI 22:6	$3.93×10^{-5}±1.25×10^{-5}$	$5.27×10^{-5}±1.92×10^{-5}$
LPI 22:5	$1.17×10^{-5}±3.84×10^{-6}$	$3.76×10^{-5}±1.22×10^{-5}$
LPI 22:4	$8.88×10^{-6}±2.45×10^{-6}$	$1.05×10^{-5}±3.58×10^{-6}$
LPI 20:4	$4.56×10^{-4}±1.08×10^{-4}$	$6.88×10^{-4}±2.48×10^{-4}$
LPI 20:3	$9.84×10^{-5}±2.65×10^{-5}$	$2.14×10^{-4}±8.14×10^{-5}$
LPI 20:2	$7.55×10^{-6}±1.94×10^{-6}$	$9.29×10^{-6}±2.66×10^{-6}$
LPI 20:0	$1.85×10^{-5}±6.36×10^{-6}$	$7.65×10^{-6}±2.32×10^{-6}$

LPI 19:0	$6.45×10^{-5}±1.21×10^{-5}$	$4.45×10^{-5}±1.34×10^{-5}$
LPI 18:2	$8.69×10^{-5}±2.76×10^{-5}$	$8.42×10^{-5}±3.34×10^{-5}$
LPI 18:1	$1.30×10^{-4}±1.32×10^{-5}$	$1.75×10^{-4}±6.09×10^{-5}$
LPI 18:0	$5.65×10^{-3}±1.55×10^{-3}$	$7.28×10^{-3}±1.03×10^{-3}$
LPI 17:0	$4.34×10^{-5}±1.07×10^{-5}$	$3.27×10^{-5}±5.03×10^{-6}$
LPI 16:0	$6.08×10^{-4}±1.71×10^{-4}$	$3.14×10^{-4}±4.45×10^{-5}$
LPG 22:6	$6.82×10^{-5}±1.31×10^{-5}$	$1.16×10^{-4}±4.46×10^{-5}$
LPG 22:5	$2.71×10^{-6}±6.04×10^{-7}$	$9.51×10^{-6}±1.80×10^{-6}$
LPG 20:4	$6.37×10^{-5}±1.23×10^{-5}$	$6.18×10^{-5}±1.84×10^{-5}$
LPG 20:3	$4.97×10^{-5}±1.47×10^{-6}$	$3.80×10^{-5}±1.71×10^{-5}$
LPG 20:2	$1.79×10^{-5}±3.70×10^{-6}$	$2.92×10^{-5}±9.43×10^{-6}$
LPG 19:0	$8.91×10^{-6}±1.65×10^{-6}$	$1.07×10^{-5}±3.49×10^{-6}$
LPG 18:2	$8.37×10^{-4}±1.29×10^{-4}$	$5.98×10^{-4}±2.52×10^{-4}$
LPG 18:1	$2.02×10^{-4}±2.98×10^{-5}$	$1.52×10^{-4}±4.42×10^{-5}$
LPG 18:0	$1.75×10^{-4}±5.27×10^{-5}$	$1.97×10^{-4}±3.15×10^{-5}$
LPG 16:1	$3.99×10^{-5}±1.31×10^{-5}$	$1.40×10^{-5}±8.89×10^{-6}$
LPG 16:0	$2.80×10^{-4}±7.02×10^{-5}$	$1.88×10^{-4}±2.31×10^{-5}$
LPE O-20:1	$3.45×10^{-6}±4.72×10^{-7}$	$6.75×10^{-6}±1.60×10^{-6}$
LPE O-18:2	$7.10×10^{-5}±2.23×10^{-5}$	$7.93×10^{-5}±2.07×10^{-5}$
LPE O-18:1	$3.31×10^{-5}±6.48×10^{-6}$	$1.04×10^{-4}±3.00×10^{-5}$
LPE O-16:1	$1.98×10^{-4}±4.64×10^{-5}$	$1.44×10^{-4}±3.31×10^{-5}$
LPE 22:6	$1.98×10^{-3}±4.78×10^{-4}$	$2.07×10^{-3}±3.41×10^{-4}$
LPE 22:5	$6.33×10^{-5}±1.34×10^{-5}$	$1.31×10^{-4}±1.54×10^{-5}$
LPE 22:4	$5.04×10^{-5}±7.54×10^{-6}$	$3.42×10^{-5}±4.61×10^{-6}$
LPE 22:1	$1.58×10^{-5}±4.52×10^{-6}$	$3.14×10^{-6}±6.58×10^{-7}$
LPE 22:0	$9.54×10^{-6}±3.28×10^{-6}$	$1.30×10^{-6}±3.32×10^{-7}$

LPE 20:4	$1.22\times10^{-3}\pm2.09\times10^{-4}$	$1.03\times10^{-3}\pm1.89\times10^{-4}$
LPE 20:3	$1.10\times10^{-4}\pm2.60\times10^{-5}$	$1.49\times10^{-4}\pm3.67\times10^{-5}$
LPE 20:2	$7.43\times10^{-5}\pm2.47\times10^{-5}$	$4.17\times10^{-5}\pm1.29\times10^{-5}$
LPE 20:1	$2.40\times10^{-4}\pm5.57\times10^{-5}$	$7.74\times10^{-5}\pm1.79\times10^{-5}$
LPE 20:0	$1.88\times10^{-4}\pm6.72\times10^{-5}$	$2.40\times10^{-5}\pm9.21\times10^{-6}$
LPE 19:0	$5.47\times10^{-5}\pm2.02\times10^{-5}$	$2.47\times10^{-5}\pm3.70\times10^{-6}$
LPE 18:3	$2.25\times10^{-5}\pm7.96\times10^{-6}$	$9.28\times10^{-6}\pm1.82\times10^{-6}$
LPE 18:2	$1.47\times10^{-3}\pm3.36\times10^{-4}$	$9.79\times10^{-4}\pm3.26\times10^{-4}$
LPE 18:1	$1.24\times10^{-3}\pm2.42\times10^{-4}$	$6.85\times10^{-3}\pm6.40\times10^{-3}$
LPE 18:0	$1.20\times10^{-2}\pm2.19\times10^{-3}$	$7.15\times10^{-3}\pm1.84\times10^{-3}$
LPE 17:0	$3.62\times10^{-4}\pm1.01\times10^{-4}$	$1.57\times10^{-4}\pm2.28\times10^{-5}$
LPE 16:1	$2.08\times10^{-4}\pm6.27\times10^{-5}$	$4.54\times10^{-5}\pm1.78\times10^{-5}$
LPE 16:0	$5.01\times10^{-3}\pm1.15\times10^{-3}$	$2.76\times10^{-3}\pm3.53\times10^{-4}$
LPC O-24:1	$4.51\times10^{-6}\pm6.77\times10^{-7}$	$8.01\times10^{-6}\pm8.50\times10^{-7}$
LPC O-24:0	$8.18\times10^{-6}\pm3.58\times10^{-6}$	$1.44\times10^{-5}\pm1.27\times10^{-6}$
LPC O-22:1	$5.90\times10^{-6}\pm1.63\times10^{-6}$	$1.24\times10^{-5}\pm2.51\times10^{-6}$
LPC O-22:0	$7.40\times10^{-6}\pm1.46\times10^{-6}$	$1.61\times10^{-5}\pm1.52\times10^{-6}$
LPC O-20:2	$4.40\times10^{-5}\pm7.13\times10^{-6}$	$7.13\times10^{-5}\pm2.07\times10^{-5}$
LPC O-20:0	$1.94\times10^{-5}\pm3.09\times10^{-6}$	$6.13\times10^{-5}\pm1.26\times10^{-5}$
LPC O-18:2	$4.89\times10^{-5}\pm7.52\times10^{-6}$	$8.50\times10^{-5}\pm2.81\times10^{-5}$
LPC O-18:1	$1.16\times10^{-3}\pm1.82\times10^{-4}$	$2.59\times10^{-3}\pm7.46\times10^{-4}$
LPC O-18:0	$1.56\times10^{-4}\pm1.60\times10^{-5}$	$2.87\times10^{-4}\pm6.86\times10^{-5}$
LPC O-16:0	$1.59\times10^{-3}\pm1.02\times10^{-4}$	$1.75\times10^{-3}\pm4.35\times10^{-4}$
LPC 24:6	$1.08\times10^{-4}\pm2.37\times10^{-5}$	$9.26\times10^{-5}\pm2.09\times10^{-5}$
LPC 24:5/0:0	$6.63\times10^{-5}\pm9.03\times10^{-6}$	$2.03\times10^{-5}\pm3.14\times10^{-6}$
LPC 24:2/0:0	$5.34\times10^{-6}\pm8.70\times10^{-7}$	$2.44\times10^{-6}\pm5.07\times10^{-7}$

LPC 24:1/0:0	$3.41\times10^{-5}\pm6.19\times10^{-6}$	$1.00\times10^{-5}\pm3.37\times10^{-6}$
LPC 24:0/0:0	$8.14\times10^{-5}\pm1.19\times10^{-5}$	$2.67\times10^{-5}\pm3.80\times10^{-6}$
LPC 23:0/0:0	$1.03\times10^{-5}\pm1.24\times10^{-6}$	$3.41\times10^{-6}\pm1.29\times10^{-7}$
LPC 22:6/0:0	$3.87\times10^{-2}\pm7.31\times10^{-3}$	$3.90\times10^{-2}\pm8.97\times10^{-3}$
LPC 22:5/0:0	$1.86\times10^{-3}\pm3.82\times10^{-4}$	$3.77\times10^{-3}\pm7.74\times10^{-4}$
LPC 22:4/0:0	$4.69\times10^{-4}\pm5.11\times10^{-5}$	$3.04\times10^{-4}\pm9.34\times10^{-5}$
LPC 22:3/0:0	$5.50\times10^{-5}\pm6.39\times10^{-6}$	$4.86\times10^{-5}\pm1.08\times10^{-5}$
LPC 22:2/0:0	$3.16\times10^{-5}\pm4.21\times10^{-6}$	$8.81\times10^{-6}\pm1.02\times10^{-6}$
LPC 22:1/0:0	$6.62\times10^{-5}\pm1.54\times10^{-5}$	$1.95\times10^{-5}\pm4.47\times10^{-6}$
LPC 22:0/0:0	$1.00\times10^{-4}\pm2.35\times10^{-5}$	$2.68\times10^{-5}\pm4.35\times10^{-6}$
LPC 21:0/0:0	$1.21\times10^{-5}\pm2.59\times10^{-6}$	$2.33\times10^{-6}\pm3.32\times10^{-7}$
LPC 20:5	$1.84\times10^{-3}\pm3.62\times10^{-4}$	$4.44\times10^{-4}\pm1.06\times10^{-4}$
LPC 20:4/0:0	$3.67\times10^{-2}\pm5.90\times10^{-3}$	$2.24\times10^{-2}\pm4.45\times10^{-3}$
LPC 20:3/0:0	$1.03\times10^{-2}\pm2.31\times10^{-3}$	$1.02\times10^{-2}\pm3.00\times10^{-3}$
LPC 20:2/0:0	$1.62\times10^{-3}\pm4.17\times10^{-4}$	$1.50\times10^{-3}\pm3.62\times10^{-4}$
LPC 20:1	$3.66\times10^{-4}\pm5.17\times10^{-5}$	$1.10\times10^{-4}\pm2.59\times10^{-5}$
LPC 20:0/0:0	$1.28\times10^{-3}\pm3.82\times10^{-4}$	$1.65\times10^{-4}\pm8.15\times10^{-5}$
LPC 19:1	$2.83\times10^{-4}\pm7.04\times10^{-5}$	$1.12\times10^{-4}\pm2.72\times10^{-5}$
LPC 19:0	$1.09\times10^{-4}\pm2.12\times10^{-5}$	$1.76\times10^{-5}\pm2.57\times10^{-6}$
LPC 18:3/0:0	$6.53\times10^{-4}\pm1.44\times10^{-4}$	$3.62\times10^{-4}\pm7.54\times10^{-5}$
LPC 18:2	$6.64\times10^{-2}\pm1.39\times10^{-2}$	$3.02\times10^{-2}\pm9.22\times10^{-3}$
LPC 18:1/0:0	$2.86\times10^{-2}\pm5.91\times10^{-3}$	$2.29\times10^{-2}\pm4.20\times10^{-3}$
LPC 18:0	$2.45\times10^{-3}\pm1.72\times10^{-4}$	$3.75\times10^{-3}\pm4.73\times10^{-4}$
LPC 17:1/0:0	$3.35\times10^{-4}\pm9.61\times10^{-5}$	$1.68\times10^{-4}\pm3.93\times10^{-5}$
LPC 17:0	$5.56\times10^{-4}\pm1.52\times10^{-4}$	$2.74\times10^{-4}\pm7.94\times10^{-5}$
LPC 16:1/0:0	$2.15\times10^{-3}\pm6.04\times10^{-4}$	$9.35\times10^{-4}\pm2.73\times10^{-4}$

LPC 16:0/0:0	$9.67\times10^{-2}\pm1.33\times10^{-2}$	$6.78\times10^{-2}\pm7.03\times10^{-3}$
LPC 15:0/0:0	$4.32\times10^{-4}\pm1.04\times10^{-4}$	$2.32\times10^{-4}\pm2.20\times10^{-5}$
LPC 14:0/0:0	$1.18\times10^{-4}\pm3.92\times10^{-5}$	$1.46\times10^{-4}\pm3.46\times10^{-5}$
FAHFA 25:6|FAHFA 22:6/3:0	$9.99\times10^{-5}\pm2.52\times10^{-5}$	$6.89\times10^{-5}\pm1.90\times10^{-5}$
FAHFA 25:5|FAHFA 22:5/3:0	$2.06\times10^{-5}\pm4.51\times10^{-6}$	$3.46\times10^{-6}\pm1.16\times10^{-6}$
FAHFA 23:4|FAHFA 20:4/3:0	$1.47\times10^{-4}\pm1.94\times10^{-5}$	$7.11\times10^{-5}\pm2.33\times10^{-5}$
FAHFA 23:3|FAHFA 20:3/3:0	$3.12\times10^{-5}\pm7.75\times10^{-6}$	$3.29\times10^{-5}\pm1.25\times10^{-5}$
FAHFA 21:2|FAHFA 18:2/3:0	$3.96\times10^{-4}\pm9.33\times10^{-5}$	$3.06\times10^{-4}\pm8.06\times10^{-5}$
FAHFA 21:1|FAHFA 18:1/3:0	$4.48\times10^{-5}\pm1.65\times10^{-5}$	$2.08\times10^{-4}\pm8.23\times10^{-5}$
FAHFA 20:1|FAHFA 18:1/2:0	$9.83\times10^{-6}\pm1.97\times10^{-6}$	$1.08\times10^{-5}\pm1.01\times10^{-5}$
FAHFA 20:0|FAHFA 18:0/2:0	$1.92\times10^{-5}\pm1.33\times10^{-5}$	$4.90\times10^{-5}\pm3.57\times10^{-5}$
FAHFA 18:0|FAHFA 16:0/2:0	$6.83\times10^{-5}\pm5.35\times10^{-5}$	$9.77\times10^{-5}\pm8.09\times10^{-5}$
FA 28:6	$7.77\times10^{-6}\pm1.45\times10^{-6}$	$1.07\times10^{-5}\pm1.55\times10^{-6}$
FA 24:5	$2.30\times10^{-5}\pm4.42\times10^{-6}$	$1.62\times10^{-6}\pm4.05\times10^{-7}$
FA 22:6	$2.96\times10^{-3}\pm5.73\times10^{-4}$	$1.71\times10^{-3}\pm4.67\times10^{-4}$
FA 22:5	$1.34\times10^{-4}\pm3.73\times10^{-5}$	$1.85\times10^{-5}\pm3.27\times10^{-6}$
FA 22:4	$9.76\times10^{-6}\pm3.57\times10^{-6}$	$2.96\times10^{-5}\pm8.44\times10^{-6}$
FA 20:5	$2.36\times10^{-4}\pm7.72\times10^{-5}$	$2.39\times10^{-5}\pm7.89\times10^{-6}$
FA 20:4	$1.41\times10^{-3}\pm1.11\times10^{-4}$	$1.15\times10^{-3}\pm2.48\times10^{-4}$
FA 20:3	$3.11\times10^{-5}\pm9.76\times10^{-6}$	$9.25\times10^{-5}\pm2.79\times10^{-5}$
FA 20:2	$1.83\times10^{-5}\pm1.42\times10^{-6}$	$4.16\times10^{-6}\pm1.30\times10^{-6}$
FA 20:0	$1.66\times10^{-5}\pm5.10\times10^{-6}$	$6.96\times10^{-8}\pm3.26\times10^{-8}$
FA 18:2	$5.02\times10^{-4}\pm8.63\times10^{-5}$	$3.66\times10^{-4}\pm8.38\times10^{-5}$
FA 18:1	$1.93\times10^{-4}\pm3.66\times10^{-5}$	$4.06\times10^{-4}\pm1.14\times10^{-4}$
FA 18:0	$4.08\times10^{-5}\pm1.13\times10^{-5}$	$1.28\times10^{-4}\pm2.43\times10^{-5}$
FA 16:0	$9.66\times10^{-5}\pm1.55\times10^{-5}$	$1.39\times10^{-4}\pm3.13\times10^{-5}$

DG 52:3|DG 34:1_18:2	$6.93\times10^{-5}\pm1.81\times10^{-5}$	$3.84\times10^{-5}\pm1.78\times10^{-5}$
DG 44:6|DG 18:0_26:6	$5.27\times10^{-4}\pm1.35\times10^{-4}$	$7.74\times10^{-4}\pm4.34\times10^{-4}$
DG 42:7|DG 18:1_24:6	$6.50\times10^{-5}\pm1.10\times10^{-5}$	$3.83\times10^{-5}\pm1.47\times10^{-5}$
DG 40:7|DG 18:2_22:5	$3.85\times10^{-4}\pm1.40\times10^{-4}$	$3.74\times10^{-6}\pm2.66\times10^{-6}$
DG 40:7|DG 18:1_22:6	$1.53\times10^{-3}\pm4.49\times10^{-4}$	$4.83\times10^{-4}\pm1.06\times10^{-4}$
DG 40:6|DG 18:2_22:4	$3.09\times10^{-4}\pm9.04\times10^{-5}$	$1.32\times10^{-5}\pm9.18\times10^{-6}$
DG 40:6|DG 16:0_24:6	$1.68\times10^{-4}\pm3.50\times10^{-5}$	$9.87\times10^{-5}\pm5.33\times10^{-5}$
DG 40:4|DG 18:0_22:4	$1.36\times10^{-4}\pm4.23\times10^{-5}$	$8.67\times10^{-5}\pm3.90\times10^{-5}$
DG 40:3|DG 22:1_18:2	$1.83\times10^{-4}\pm4.66\times10^{-5}$	$4.18\times10^{-5}\pm2.85\times10^{-5}$
DG 40:2|DG 22:0_18:2	$9.61\times10^{-5}\pm3.44\times10^{-5}$	$2.23\times10^{-5}\pm1.18\times10^{-5}$
DG 38:5|DG 18:1_20:4	$5.72\times10^{-4}\pm1.28\times10^{-4}$	$2.82\times10^{-4}\pm8.84\times10^{-5}$
DG 38:3|DG 18:0_20:3	$6.83\times10^{-4}\pm1.98\times10^{-4}$	$7.25\times10^{-4}\pm2.01\times10^{-4}$
DG 38:2|DG 20:0_18:2	$4.22\times10^{-4}\pm7.51\times10^{-5}$	$3.14\times10^{-4}\pm9.00\times10^{-5}$
DG 38:1|DG 20:0_18:1	$6.93\times10^{-5}\pm1.93\times10^{-5}$	$4.16\times10^{-5}\pm9.31\times10^{-6}$
DG 37:3|DG 19:1_18:2	$6.90\times10^{-5}\pm2.77\times10^{-5}$	$2.46\times10^{-5}\pm7.86\times10^{-6}$
DG 36:6|DG 18:2_18:4	$2.10\times10^{-5}\pm5.98\times10^{-6}$	$9.40\times10^{-7}\pm9.43\times10^{-7}$
DG 36:5|DG 18:2_18:3	$4.45\times10^{-4}\pm2.22\times10^{-4}$	$2.25\times10^{-6}\pm1.79\times10^{-6}$
DG 36:4|DG 18:2_18:2	$3.34\times10^{-3}\pm1.74\times10^{-3}$	$1.05\times10^{-4}\pm5.69\times10^{-5}$
DG 36:4|DG 16:0_20:4	$3.55\times10^{-4}\pm3.39\times10^{-5}$	$4.53\times10^{-5}\pm1.63\times10^{-5}$
DG 36:3|DG 18:1_18:2	$5.00\times10^{-3}\pm2.00\times10^{-3}$	$2.19\times10^{-4}\pm7.86\times10^{-5}$
DG 36:2|DG 18:0_18:2	$2.31\times10^{-3}\pm2.72\times10^{-4}$	$2.30\times10^{-3}\pm5.73\times10^{-4}$
DG 36:1|DG 18:0_18:1	$2.47\times10^{-4}\pm3.55\times10^{-5}$	$3.98\times10^{-4}\pm3.99\times10^{-5}$
DG 36:0|DG 18:0_18:0	$1.36\times10^{-4}\pm2.58\times10^{-5}$	$1.94\times10^{-4}\pm6.24\times10^{-5}$
DG 35:3|DG 17:1_18:2	$4.94\times10^{-5}\pm1.82\times10^{-5}$	$1.24\times10^{-6}\pm9.71\times10^{-7}$
DG 35:2|DG 17:0_18:2	$7.78\times10^{-5}\pm1.57\times10^{-5}$	$2.87\times10^{-5}\pm7.03\times10^{-6}$
DG 34:4|DG 16:1_18:3	$4.39\times10^{-5}\pm2.54\times10^{-5}$	$1.56\times10^{-7}\pm1.92\times10^{-7}$

DG 34:3|DG 16:1_18:2	$4.91\times10^{-4}\pm2.95\times10^{-4}$	$1.32\times10^{-5}\pm1.13\times10^{-5}$
DG 34:3|DG 16:0_18:3	$1.38\times10^{-4}\pm3.19\times10^{-5}$	$5.21\times10^{-6}\pm4.44\times10^{-6}$
DG 34:2|DG 16:0_18:2	$3.28\times10^{-3}\pm6.72\times10^{-4}$	$2.17\times10^{-4}\pm8.83\times10^{-5}$
DG 34:1|DG 16:0_18:1	$9.72\times10^{-4}\pm2.60\times10^{-4}$	$6.58\times10^{-4}\pm9.01\times10^{-5}$
DG 34:0|DG 16:0_18:0	$1.24\times10^{-4}\pm1.95\times10^{-5}$	$1.19\times10^{-4}\pm4.28\times10^{-5}$
DG 32:3|DG 14:1_18:2	$1.15\times10^{-5}\pm1.97\times10^{-5}$	-
DG 32:2|DG 14:0_18:2	$5.32\times10^{-5}\pm3.98\times10^{-5}$	$1.44\times10^{-6}\pm7.70\times10^{-7}$
DG 32:1|DG 16:0_16:1	$6.26\times10^{-5}\pm1.42\times10^{-5}$	-
DG 32:0|DG 16:0_16:0	$3.54\times10^{-5}\pm7.15\times10^{-6}$	$2.53\times10^{-5}\pm6.53\times10^{-6}$
CE 22:6	$1.51\times10^{-5}\pm8.01\times10^{-6}$	$8.74\times10^{-5}\pm1.42\times10^{-5}$
CE 18:1	$2.81\times10^{-5}\pm2.13\times10^{-5}$	$5.33\times10^{-5}\pm6.05\times10^{-5}$
CAR 24:2	$5.03\times10^{-6}\pm1.19\times10^{-6}$	$8.71\times10^{-6}\pm3.31\times10^{-6}$
CAR 24:1	$1.57\times10^{-5}\pm3.25\times10^{-6}$	$3.11\times10^{-5}\pm1.71\times10^{-5}$
CAR 24:0	$1.73\times10^{-5}\pm3.03\times10^{-6}$	$1.41\times10^{-5}\pm8.48\times10^{-6}$
CAR 23:4	$6.23\times10^{-8}\pm8.15\times10^{-8}$	$5.73\times10^{-5}\pm7.28\times10^{-6}$
CAR 23:3	$8.21\times10^{-6}\pm2.16\times10^{-6}$	$3.21\times10^{-5}\pm3.25\times10^{-6}$
CAR 23:2	$7.23\times10^{-6}\pm2.34\times10^{-6}$	$1.40\times10^{-5}\pm7.84\times10^{-6}$
CAR 23:1	$1.31\times10^{-5}\pm5.84\times10^{-6}$	$2.81\times10^{-5}\pm1.52\times10^{-5}$
CAR 22:6	$1.52\times10^{-5}\pm7.44\times10^{-6}$	$5.26\times10^{-5}\pm1.17\times10^{-5}$
CAR 22:1	$2.94\times10^{-5}\pm1.00\times10^{-5}$	$5.66\times10^{-5}\pm3.10\times10^{-5}$
CAR 22:0	$4.06\times10^{-5}\pm1.83\times10^{-5}$	$6.40\times10^{-5}\pm2.92\times10^{-5}$
CAR 21:1	$8.99\times10^{-5}\pm2.42\times10^{-5}$	$1.62\times10^{-4}\pm5.00\times10^{-5}$
CAR 21:0	$6.27\times10^{-5}\pm1.64\times10^{-5}$	$1.30\times10^{-4}\pm4.84\times10^{-5}$
CAR 20:4	$4.19\times10^{-5}\pm7.04\times10^{-6}$	$1.30\times10^{-4}\pm2.05\times10^{-5}$
CAR 20:0	$2.21\times10^{-5}\pm8.33\times10^{-6}$	$4.43\times10^{-5}\pm1.97\times10^{-5}$
CAR 19:0	$4.84\times10^{-5}\pm1.07\times10^{-5}$	$8.48\times10^{-5}\pm1.22\times10^{-5}$

CAR 18:2	$1.07×10^{-4}±3.48×10^{-5}$	$1.20×10^{-4}±1.40×10^{-5}$
CAR 18:1	$2.17×10^{-4}±1.27×10^{-4}$	$1.92×10^{-4}±5.52×10^{-5}$
CAR 18:0	$1.38×10^{-4}±3.65×10^{-5}$	$2.71×10^{-4}±3.78×10^{-5}$
BMP 40:8|BMP 18:2_22:6	$1.13×10^{-5}±1.82×10^{-6}$	$8.09×10^{-6}±1.77×10^{-6}$

"-" indicates not detected.

Table S2. Results of volcano maps for CON and HFD groups.

Name	FC	log2(FC)	raw.pval
CAR 20:4	3.2341	1.6934	7.01E-05
CAR 21:0	2.2319	1.1583	2.97E-02
CAR 22:6	3.7927	1.9232	3.15E-04
CAR 23:3	4.1589	2.0562	4.06E-07
CAR 23:4	864.12	9.7551	5.47E-08
CE 22:6	6.2515	2.6442	4.24E-05
DG 16:0_16:1	0.087676	-3.5117	6.18E-05

DG 16:0_18:2	0.06867	-3.8642	7.28E-06
DG 16:0_18:3	0.038223	-4.7094	2.20E-05
DG 16:1_18:2	0.024858	-5.3301	4.01E-02
DG 16:1_18:3	0.0041884	-7.8994	3.22E-02
DG 17:0_18:2	0.37606	-1.411	1.64E-03
DG 17:1_18:2	0.024493	-5.3515	5.82E-03
DG 18:1_18:2	0.042575	-4.5539	1.11E-02
DG 16:0_20:4	0.13219	-2.9193	4.99E-06
DG 18:2_18:2	0.029818	-5.0677	2.61E-02
DG 18:2_18:3	0.0047651	-7.7133	2.03E-02
DG 18:2_18:4	0.045833	-4.4475	9.18E-05
DG 22:0_18:2	0.25317	-1.9818	2.54E-03
DG 22:1_18:2	0.24305	-2.0407	2.21E-04
DG 18:2_22:4	0.044876	-4.4779	4.40E-05
DG 18:1_22:6	0.33782	-1.5657	3.50E-04
DG 18:2_22:5	0.01021	-6.6138	1.59E-04
Diisodecyl phthalate (also known as the production of plastic)	2.9952	1.5827	2.63E-04
FA 18:0	3.2033	1.6796	1.19E-04
FA 18:1	2.1537	1.1068	3.52E-03
FA 20:0	0.0044246	-7.8202	1.44E-04
FA 20:2	0.23187	-2.1086	7.32E-05
FA 20:3	3.0077	1.5886	2.19E-03
FA 20:5	0.10654	-3.2305	1.63E-04
FA 22:4	3.1238	1.6433	1.50E-03
FA 22:5	0.1461	-2.7749	7.20E-05
FA 24:5	0.072935	-3.7772	4.65E-06

FAHFA 18:1/3:0	4.7379	2.2443	3.52E-03
FAHFA 20:4/3:0	0.49578	-1.0122	6.26E-03
FAHFA 22:5/3:0	0.17533	-2.5118	7.30E-05
LPC 16:1/0:0	0.45937	-1.1223	2.62E-03
LPC 18:2	0.47194	-1.0833	1.91E-03
LPC 19:0	0.16902	-2.5647	5.79E-06
LPC 19:1	0.41531	-1.2677	8.35E-04
LPC 20:0/0:0	0.13966	-2.84	8.05E-05
LPC 20:1	0.31214	-1.6797	3.01E-05
LPC 20:5	0.25257	-1.9853	6.79E-05
LPC 21:0/0:0	0.20505	-2.286	3.76E-06
LPC 22:0/0:0	0.28578	-1.807	4.32E-05
LPC 22:1/0:0	0.31562	-1.6638	2.57E-05
LPC 22:2/0:0	0.29095	-1.7811	1.46E-05
LPC 22:5/0:0	2.1428	1.0995	7.56E-04
LPC 23:0/0:0	0.3478	-1.5237	1.65E-08
LPC 24:0/0:0	0.34706	-1.5267	2.11E-07
LPC 24:1/0:0	0.31114	-1.6844	8.82E-06
LPC 24:2/0:0	0.4612	-1.1165	1.38E-02
LPC 24:5/0:0	0.31881	-1.6492	2.03E-06
LPC O-18:1	2.3207	1.2145	5.42E-03
LPC O-20:0	3.2526	1.7016	2.62E-04
LPC O-22:0	2.2492	1.1694	6.49E-05
LPC O-22:1	2.1493	1.1039	3.80E-03
LPE 16:1	0.23179	-2.1091	3.06E-04
LPE 17:0	0.46272	-1.1118	1.20E-03

LPE 18:3	0.42894	-1.2212	1.21E-02
LPE 19:0	0.4859	-1.0413	9.88E-03
LPE 20:0	0.13834	-2.8537	5.77E-04
LPE 20:1	0.34376	-1.5405	4.04E-05
LPE 22:0	0.14778	-2.7585	4.20E-04
LPE 22:1	0.21304	-2.2308	5.50E-05
LPE 22:5	2.1653	1.1145	5.15E-05
LPE O-18:1	3.2155	1.6851	2.36E-03
LPE O-20:1	2.0466	1.0332	1.88E-03
LPG 16:1	0.36405	-1.4578	6.73E-03
LPG 22:5	3.6661	1.8742	2.45E-05
LPI 20:0	0.44971	-1.1529	7.78E-03
LPI 20:3	2.2951	1.1986	1.34E-02
LPI 22:5	3.4108	1.7701	2.05E-03
LPS 19:0	0.30055	-1.7343	3.03E-05
PA 16:0_18:2	0.050831	-4.2981	2.15E-04
PA 16:0_20:4	0.080093	-3.6422	2.86E-04
PA 18:2_18:2	17.259	4.1093	4.83E-08
PA 16:0_22:6	35.824	5.1629	3.39E-05
PC 28:0	2.7733	1.4716	1.94E-02
PC 30:0	0.32661	-1.6143	2.40E-04
PC 31:0	0.0079477	-6.9753	4.31E-07
PC 31:1	0.022237	-5.4909	1.83E-04
PC 16:0_16:0	3.3088	1.7263	1.07E-07
PC 32:1	0.14863	-2.7502	2.88E-06
PC 33:0	2.0947	1.0668	1.91E-04

PC 33:2	0.085204	-3.5529	5.49E-04
PC 34:0	4.8787	2.2865	4.68E-05
PC 34:1	14.631	3.871	3.92E-05
PC 34:2	2.488	1.315	9.90E-04
PC 34:3	0.15641	-2.6766	7.81E-07
PC 34:4	2.3464	1.2305	8.51E-03
PC 35:1	4.5094	2.1729	1.18E-05
PC 35:2	3.4422	1.7833	2.39E-03
PC 35:3	0.25479	-1.9726	1.33E-06
PC 36:1	2.4617	1.2996	4.60E-04
PC 36:2	2.8237	1.4976	1.46E-03
PC 18:0_18:2;2O	3.2151	1.6849	1.16E-02
PC 36:5	0.17899	-2.4821	4.94E-04
PC 36:6	2.0061	1.0044	9.76E-03
PC 37:3	2.4495	1.2925	2.11E-02
PC 37:4	2.7846	1.4775	1.68E-02
PC 38:3	6.1062	2.6103	1.25E-03
PC 38:4	2.7426	1.4555	1.05E-02
PC 38:6	3.5497	1.8277	7.83E-04
PC 39:1	0.086562	-3.5301	9.28E-05
PC 39:6	4.6279	2.2103	8.20E-04
PC 39:8	2.9607	1.5659	1.38E-04
PC 40:1	0.44414	-1.1709	5.07E-04
PC 40:10	2.4739	1.3068	5.78E-03
PC 40:4	0.15238	-2.7142	3.56E-03
PC 40:5	9.8755	3.3039	1.37E-03

PC 40:6	5.9092	2.563	1.16E-04
PC 40:7	2.8177	1.4945	2.09E-03
PC 40:9	0.15422	-2.6969	1.02E-04
PC 41:5	3.267	1.7079	4.99E-03
PC 41:6	0.10506	-3.2507	7.54E-05
PC 42:1	0.42905	-1.2208	2.05E-03
PC 42:10	3.0425	1.6053	2.05E-05
PC 42:11	0.13136	-2.9284	5.86E-04
PC 42:2	0.33554	-1.5754	6.00E-04
PC 42:5	0.38829	-1.3648	1.55E-02
PC 42:6	0.048348	-4.3704	1.04E-03
PC 42:7	3.2255	1.6895	5.85E-05
PC 42:8	3.9822	1.9936	3.48E-05
PC 42:9	2.0777	1.055	1.48E-03
PC 44:10	2.3	1.2016	3.46E-03
PC 44:11	11.716	3.5504	5.60E-04
PC 44:6	0.023114	-5.4351	3.51E-02
PC O-30:0	0.19165	-2.3835	3.10E-05
PC O-31:0	0.045744	-4.4503	3.80E-06
PC O-32:0	7.1972	2.8474	4.35E-04
PC O-32:1	0.37515	-1.4145	3.18E-02
PC O-33:2	0.28962	-1.7877	6.99E-03
PC O-34:1	4.4751	2.1619	2.70E-05
PC O-34:2	0.18507	-2.4339	3.38E-05
PC O-34:3	0.10688	-3.2259	8.07E-05
PC O-35:7	3.115	1.6392	2.46E-05

PC O-36:2	2.8477	1.5098	7.22E-04
PC O-36:3	0.28401	-1.816	5.54E-05
PC O-36:5	0.17614	-2.5052	7.09E-04
PC O-37:7	4.1265	2.0449	1.41E-03
PC O-38:3	3.2665	1.7077	1.05E-04
PC O-38:5	5.3709	2.4252	1.30E-05
PC O-38:6	2.7558	1.4625	2.67E-04
PC O-40:6	6.6336	2.7298	4.50E-05
PC O-40:7	5.4919	2.4573	7.08E-04
PC O-42:6	2.649	1.4055	5.44E-05
PC O-42:7	2.5807	1.3678	8.29E-05
PC O-42:9	7.7857	2.9608	4.16E-03
PC O-44:12	2.6068	1.3823	1.04E-02
PE 16:0_16:0	0.15266	-2.7116	7.08E-03
PE 16:0_18:1	4.7293	2.2416	3.27E-06
PE 16:0_18:2	4.2056	2.0723	9.92E-05
PE 16:0_18:3	0.099415	-3.3304	1.97E-03
PE 16:1_18:2	0.23675	-2.0786	2.97E-06
PE 17:0_18:1	12.75	3.6724	4.19E-07
PE 18:0_18:0	3.3596	1.7483	7.42E-04
PE 18:0_18:1	2.361	1.2394	2.04E-03
PE 18:1_18:1	20.503	4.3577	1.42E-03
PE 16:0_20:3	2.4218	1.2761	2.74E-03
PE 18:1_18:2	2.7544	1.4617	1.36E-05
PE 16:0_20:4	3.7484	1.9063	4.94E-04
PE 16:0_20:5	0.057023	-4.1323	3.23E-05

PE 16:1_20:4	0.21861	-2.1936	3.76E-04
PE 14:0_22:6	23.773	4.5713	2.00E-04
PE 19:0_18:2	0.089934	-3.475	2.08E-05
PE 18:0_20:3	5.93	2.568	2.43E-04
PE 38:4	0.4507	-1.1498	6.88E-04
PE 18:0_20:4	141.73	7.147	2.65E-05
PE 18:1_20:3	6.1748	2.6264	3.84E-06
PE 18:1_20:4;O	4.5664	2.1911	5.78E-03
PE 16:0_22:5	12.49	3.6427	2.15E-05
PE 18:1_20:4	10.787	3.4312	3.46E-05
PE 38:6	8.3851	3.0678	1.17E-02
PE 16:0_22:6;O	2.4558	1.2962	1.21E-02
PE 16:0_22:6	0.057855	-4.1114	1.22E-04
PE 18:2_20:4	4.2191	2.0769	4.68E-06
PE 16:1_22:6	3.2387	1.6954	1.56E-04
PE 19:0_20:4	4.2234	2.0784	1.40E-04
PE 17:0_22:6	32.464	5.0208	4.82E-06
PE 18:0_22:5	23.288	4.5415	3.01E-06
PE 18:0_22:6	107.91	6.7537	5.05E-06
PE 18:1_22:5	0.42868	-1.222	4.71E-03
PE 18:1_22:6	92.765	6.5355	1.47E-03
PE 19:0_22:6	0.056776	-4.1386	1.57E-02
PE 42:6	0.020464	-5.6108	1.94E-03
PE 20:0_22:6	9.8233	3.2962	1.43E-02
PE 42:8	2.7864	1.4784	1.44E-02
PE 20:2_22:6	26.766	4.7423	9.96E-09

PE 42:9	2.2671	1.1809	1.68E-05
PE 22:6_22:6	87.274	6.4475	6.13E-04
PE O-20:5	0.30372	-1.7192	7.45E-06
PE O-16:1_18:2	0.0073901	-7.0802	3.16E-03
PE O-18:2_18:1	0.4308	-1.2149	1.58E-03
PE O-16:1_20:3	0.40561	-1.3018	2.95E-02
PE O-16:1_20:4	68.584	6.0998	1.27E-02
PE O-18:1_20:1	5.4666	2.4506	3.33E-05
PE O-16:1_22:5	3.535	1.8217	7.38E-05
PE O-16:1_22:6	186.32	7.5417	1.03E-05
PG 16:0_16:0	0.12978	-2.9459	1.90E-02
PG 16:0_16:1	0.03629	-4.7843	9.35E-03
PG 16:0_18:1	6.1232	2.6143	1.01E-06
PG 16:0_18:2	0.047393	-4.3992	1.48E-03
PG 16:0_19:1	401.16	8.648	2.02E-05
PG 17:0_18:1	0.14092	-2.827	1.08E-06
PG 17:0_18:2	0.13685	-2.8693	1.76E-03
PG 18:0_18:1	117.65	6.8784	2.88E-07
PG 18:0_18:2	148.41	7.2134	2.26E-08
PG 18:1_18:1	4.8444	2.2763	1.42E-06
PG 18:1_18:2	3.5728	1.8371	5.01E-04
PG 16:0_20:4	0.18665	-2.4216	2.88E-05
PG 18:2_18:2	0.27322	-1.8719	9.92E-06
PG 18:0_20:2	199.73	7.6419	2.94E-03
PG 18:1_20:2	3.5991	1.8476	5.21E-05
PG 20:1_18:2	9.0054	3.1708	7.02E-05

PG 16:0_22:6	0.31172	-1.6817	4.40E-02
PG 18:2_20:4	4.2502	2.0875	1.08E-03
PG 16:1_22:6	0.39704	-1.3326	1.02E-03
PG 18:1_22:4	5.5609	2.4753	4.97E-06
PG 18:1_22:5	2.9552	1.5632	4.57E-04
PG 20:4_22:6	14.315	3.8394	3.19E-05
PG 20:5_22:6	0.031922	-4.9693	2.05E-04
PG 22:5_22:6	17.02	4.0892	2.07E-05
PG 22:6_22:6	0.14278	-2.8081	1.04E-03
PI 16:0_18:2	2.1785	1.1233	1.81E-02
PI 16:0_18:3	9.0883	3.184	4.81E-04
PI 17:0_18:2	26.277	4.7157	2.62E-03
PI 36:0	2.8708	1.5214	8.78E-03
PI 18:0_18:0	3.7907	1.9225	1.79E-04
PI 36:1	7.8015	2.9638	5.63E-05
PI 18:0_18:1	25031	14.611	2.16E-03
PI 18:0_18:2	78.294	6.2908	7.15E-06
PI 18:1_18:1	5.3154	2.4102	1.15E-05
PI 16:0_20:3	4.8533	2.279	1.98E-06
PI 18:1_18:2	0.2614	-1.9357	2.52E-04
PI 36:4	0.098155	-3.3488	7.11E-04
PI 16:0_20:4	0.40784	-1.2939	4.21E-03
PI 18:1_18:3	0.49298	-1.0204	8.21E-03
PI 16:0_20:5	2.0874	1.0617	7.39E-03
PI 19:0_18:2	0.14424	-2.7935	1.04E-04
PI 17:0_20:3	20.969	4.3902	1.97E-05

PI 37:4	0.093376	-3.4208	2.24E-03
PI 17:0_20:4	0.018301	-5.7719	1.56E-04
PI 17:1_20:4	0.027886	-5.1643	1.52E-05
PI 18:0_20:1	3.0398	1.604	1.02E-04
PI 18:0_20:2	3.3886	1.7607	5.79E-03
PI 18:0_20:3	310.76	8.2796	6.77E-04
PI 18:0_20:4;O	5.0083	2.3243	3.52E-05
PI 18:0_20:4	27.32	4.7719	9.54E-07
PI 18:1_20:3	5.3043	2.4072	9.49E-03
PI 18:0_20:5;3O	2.2457	1.1672	2.43E-02
PI 16:0_22:5	3.3821	1.7579	7.51E-04
PI 18:0_20:5	0.17985	-2.4752	2.69E-04
PI 18:1_20:4	0.0046024	-7.7634	2.13E-02
PI 18:2_20:3	0.31568	-1.6635	4.76E-04
PI 16:0_22:6	0.1922	-2.3793	1.48E-03
PI 18:2_20:4	2.2288	1.1563	2.14E-03
PI 19:0_20:3	0.13869	-2.8501	1.94E-03
PI 39:4	0.038956	-4.682	2.44E-04
PI 19:0_20:4	2.8749	1.5235	2.71E-04
PI 39:6	0.031392	-4.9935	8.37E-04
PI 18:0_22:3	251	7.9715	3.12E-05
PI 20:0_20:3	0.032855	-4.9277	3.51E-05
PI 18:0_22:4	33.582	5.0696	3.64E-07
PI 20:0_20:4	0.059209	-4.078	1.24E-02
PI 20:1_20:3	2.5164	1.3313	1.09E-03
PI 18:0_22:5	5.5698	2.4776	1.96E-05

PI 18:0_22:6	5.725	2.5173	6.96E-04
PI 18:1_22:6	4.5467	2.1848	7.54E-06
PI 20:4_20:4	73.525	6.2002	1.44E-03
PI 20:4_22:6	26.418	4.7234	8.02E-04
PI 20:3_22:6	1705.8	10.736	8.19E-03
PMeOH 16:0_18:2	0.28876	-1.7921	3.08E-02
PMeOH 18:0_18:2	14.945	3.9016	2.33E-07
PMeOH 16:0_20:4	147.78	7.2073	2.11E-03
PS 36:1	13.455	3.7501	4.94E-04
PS 40:6	2.1602	1.1112	4.02E-02
PS 42:9	3.1877	1.6725	3.80E-02
SM 18:1;2O/14:0	2.0541	1.0385	5.64E-04
SM 34:0;2O	3.8915	1.9603	1.16E-04
SM 34:2;2O	0.46539	-1.1035	2.10E-03
SM 35:5;2O	0.37014	-1.4339	6.49E-03
SM 36:0;2O	5.9341	2.569	7.46E-03
SM 16:1;2O/20:0	3.4178	1.7731	1.42E-03
SM 18:1;2O/18:0	8.7129	3.1232	6.53E-05
SM 18:2;2O/18:0	3.3796	1.7569	1.42E-03
SM 38:0;2O	3.5582	1.8311	2.60E-03
SM 38:2;2O	4.0186	2.0067	7.15E-04
SM 39:0;2O	0.40405	-1.3074	3.10E-04
SM 17:1;2O/22:1	0.11402	-3.1326	9.98E-05
SM 41:0;2O	0.38318	-1.3839	2.71E-04
SM 18:1;2O/26:1	2.5706	1.3621	2.40E-04
SM 44:6;2O	0.42031	-1.2505	1.26E-02

SM 52:2;2O	2.3496	1.2324	1.38E-02
SM 54:2;2O	299.25	8.2252	3.53E-02
SM 57:2;2O	0.44128	-1.1802	1.70E-02
SM 58:3;2O	2.7419	1.4552	1.63E-02
SM 59:2;2O	0.47521	-1.0734	6.63E-04
SM 60:3;2O	3.0714	1.6189	9.59E-03
SM 60:4;2O	8.4958	3.0867	2.09E-03
TG 16:0_16:0_18:3	0.17975	-2.476	4.02E-02
TG 15:0_18:1_18:1	0.24984	-2.0009	3.72E-02
TG 16:0_18:1_18:2	0.43438	-1.203	1.25E-02
TG 16:0_18:1_18:3	0.21001	-2.2515	1.01E-02
TG 16:1_18:2_18:3	0.062826	-3.9925	3.80E-02
TG 18:2_18:2_18:3	0.059144	-4.0796	3.47E-05
TG 18:0_19:0_18:1	0.038019	-4.7171	3.45E-02
TG 19:0_18:1_18:1	0.24983	-2.001	4.50E-02
TG 16:0_22:0_18:1	0.10528	-3.2477	2.42E-04
TG 18:0_20:0_18:1	0.39123	-1.3539	2.67E-03
TG 16:0_18:1_22:1	0.3798	-1.3967	5.56E-04
TG 20:0_18:1_18:1	0.30033	-1.7354	1.98E-04
TG 16:0_18:1_22:5	0.22287	-2.1657	9.35E-05
TG 18:0_21:0_18:2	0.13142	-2.9278	1.01E-03
TG 16:0_18:1_24:1	0.081075	-3.6246	1.62E-06
TG 16:0_20:1_22:1	0.17296	-2.5315	1.96E-03
TG 18:0_18:1_22:1	0.16733	-2.5792	2.45E-03
TG 18:1_18:1_22:1	0.45089	-1.1492	3.20E-02
TG 22:1_18:2_18:2	0.38612	-1.3729	9.54E-03

TG 18:1_18:1_24:1	0.27726	-1.8507	3.80E-02
TG 20:1_20:1_20:1	0.16052	-2.6392	2.59E-02
TG 26:0_18:1_18:1	0.026734	-5.2252	2.98E-02

Table S3. The relative content of lipids in HFD, HAD, HBD, and HGD groups (Mean ± SD).

Name	HFD	HAD	HBD	HGD
TG 72:2|TG 36:0_18:1_18:1	$3.82\times10^{-6}\pm2.43\times10^{-6}$	$8.38\times10^{-6}\pm6.84\times10^{-6}$	$4.76\times10^{-6}\pm2.56\times10^{-6}$	$2.18\times10^{-6}\pm1.05\times10^{-6}$
TG 70:3|TG 18:1_18:1_34:1	$2.76\times10^{-5}\pm1.86\times10^{-5}$	$5.12\times10^{-5}\pm4.24\times10^{-5}$	$3.49\times10^{-5}\pm1.86\times10^{-5}$	$1.16\times10^{-5}\pm7.17\times10^{-6}$
TG 70:2|TG 18:0_18:1_34:1	$1.05\times10^{-5}\pm7.34\times10^{-6}$	$2.14\times10^{-5}\pm1.63\times10^{-5}$	$1.34\times10^{-5}\pm8.06\times10^{-6}$	$5.60\times10^{-6}\pm3.18\times10^{-6}$
TG 70:2|TG 16:0_18:1_36:1	$9.63\times10^{-6}\pm6.30\times10^{-6}$	$2.07\times10^{-5}\pm1.56\times10^{-5}$	$1.29\times10^{-5}\pm7.80\times10^{-6}$	$5.76\times10^{-6}\pm3.10\times10^{-6}$
TG 64:3|TG 18:1_18:1_28:1	-	$3.33\times10^{-6}\pm3.45\times10^{-6}$	$3.92\times10^{-7}\pm7.84\times10^{-7}$	-
TG 64:2|TG 28:0_18:1_18:1	-	$2.29\times10^{-6}\pm3.35\times10^{-6}$	-	-
TG 60:3|TG 20:1_20:1_20:1	$3.70\times10^{-5}\pm6.20\times10^{-5}$	$5.10\times10^{-5}\pm3.82\times10^{-5}$	$2.88\times10^{-5}\pm2.63\times10^{-5}$	$4.01\times10^{-6}\pm6.08\times10^{-6}$
TG 60:3|TG 18:1_18:1_24:1	$6.29\times10^{-5}\pm7.51\times10^{-5}$	$7.78\times10^{-5}\pm5.11\times10^{-5}$	$4.88\times10^{-5}\pm3.23\times10^{-5}$	$1.59\times10^{-5}\pm1.61\times10^{-5}$
TG 58:5|TG 22:1_18:2_18:2	$2.27\times10^{-4}\pm1.20\times10^{-4}$	$1.69\times10^{-4}\pm4.13\times10^{-5}$	$2.18\times10^{-4}\pm5.19\times10^{-5}$	$8.59\times10^{-5}\pm5.73\times10^{-5}$
TG 58:3|TG 18:1_18:1_22:1	$2.72\times10^{-4}\pm2.02\times10^{-4}$	$3.28\times10^{-4}\pm2.40\times10^{-4}$	$2.82\times10^{-4}\pm8.83\times10^{-5}$	$1.63\times10^{-4}\pm1.04\times10^{-4}$
TG 58:2|TG 18:0_18:1_22:1	$1.23\times10^{-4}\pm1.10\times10^{-4}$	$9.63\times10^{-5}\pm7.72\times10^{-5}$	$1.09\times10^{-4}\pm5.82\times10^{-5}$	$5.69\times10^{-5}\pm6.12\times10^{-5}$
TG 58:2|TG 16:0_20:1_22:1	$1.56\times10^{-4}\pm1.16\times10^{-4}$	$1.68\times10^{-4}\pm9.42\times10^{-5}$	$1.80\times10^{-4}\pm6.75\times10^{-5}$	$6.34\times10^{-5}\pm5.41\times10^{-5}$
TG 58:2|TG 16:0_18:1_24:1	$3.08\times10^{-5}\pm6.17\times10^{-5}$	$7.58\times10^{-5}\pm5.90\times10^{-5}$	$2.44\times10^{-5}\pm4.14\times10^{-5}$	$1.09\times10^{-6}\pm2.18\times10^{-6}$
TG 57:2|TG 18:0_21:0_18:2	$2.07\times10^{-5}\pm1.51\times10^{-5}$	$5.46\times10^{-5}\pm3.74\times10^{-5}$	$3.61\times10^{-5}\pm2.74\times10^{-5}$	$7.29\times10^{-6}\pm1.30\times10^{-5}$
TG 56:6|TG 16:0_18:1_22:5	$2.43\times10^{-3}\pm1.55\times10^{-3}$	$1.51\times10^{-3}\pm4.85\times10^{-4}$	$1.90\times10^{-3}\pm3.25\times10^{-4}$	$6.00\times10^{-4}\pm2.10\times10^{-4}$
TG 56:2|TG 20:0_18:1_18:1	$8.89\times10^{-4}\pm2.77\times10^{-4}$	$1.16\times10^{-3}\pm3.94\times10^{-4}$	$1.06\times10^{-3}\pm3.65\times10^{-4}$	$4.21\times10^{-4}\pm2.78\times10^{-4}$
TG 56:2|TG 16:0_18:1_22:1	$9.61\times10^{-4}\pm4.28\times10^{-4}$	$1.25\times10^{-3}\pm8.27\times10^{-4}$	$1.02\times10^{-3}\pm3.28\times10^{-4}$	$6.72\times10^{-4}\pm5.10\times10^{-4}$
TG 56:1|TG 18:0_20:0_18:1	$2.71\times10^{-4}\pm1.10\times10^{-4}$	$2.64\times10^{-4}\pm2.03\times10^{-4}$	$3.01\times10^{-4}\pm1.81\times10^{-4}$	$1.68\times10^{-4}\pm1.46\times10^{-4}$

TG 56:1|TG 16:0_22:0_18:1	$1.06×10^{-4}±8.87×10^{-5}$	$2.22×10^{-4}±1.51×10^{-4}$	$9.69×10^{-5}±6.32×10^{-5}$	$6.08×10^{-5}±6.02×10^{-5}$
TG 55:5|TG 19:1_18:2_18:2	$6.18×10^{-5}±3.47×10^{-5}$	$1.26×10^{-4}±1.13×10^{-4}$	$5.76×10^{-5}±1.63×10^{-5}$	$1.92×10^{-5}±1.11×10^{-5}$
TG 55:2|TG 19:0_18:1_18:1	$1.52×10^{-4}±1.05×10^{-4}$	$2.70×10^{-4}±1.21×10^{-4}$	$2.41×10^{-4}±1.04×10^{-4}$	$4.51×10^{-5}±3.46×10^{-5}$
TG 55:2|TG 17:0_18:1_20:1	$2.29×10^{-4}±1.35×10^{-4}$	$5.90×10^{-4}±3.55×10^{-4}$	$2.75×10^{-4}±9.83×10^{-5}$	$6.66×10^{-5}±5.31×10^{-5}$
TG 55:1|TG 18:0_19:0_18:1	$4.11×10^{-6}±5.26×10^{-6}$	$9.74×10^{-5}±7.35×10^{-5}$	$2.38×10^{-5}±4.75×10^{-5}$	-
TG 54:7|TG 18:2_18:2_18:3	$3.03×10^{-4}±2.02×10^{-4}$	$3.47×10^{-4}±2.60×10^{-4}$	$2.49×10^{-4}±1.21×10^{-4}$	$5.38×10^{-5}±3.25×10^{-5}$
TG 54:6|TG 18:2_18:2_18:2	$1.43×10^{-3}±9.67×10^{-4}$	$4.12×10^{-3}±3.85×10^{-3}$	$1.14×10^{-3}±4.76×10^{-4}$	$4.16×10^{-4}±2.12×10^{-4}$
TG 54:4|TG 18:1_18:1_18:2	$4.84×10^{-3}±2.52×10^{-3}$	$1.22×10^{-2}±8.08×10^{-3}$	$5.61×10^{-3}±1.94×10^{-3}$	$2.63×10^{-3}±1.63×10^{-3}$
TG 54:3|TG 18:0_18:1_18:2	$6.47×10^{-3}±3.06×10^{-3}$	$1.51×10^{-2}±6.41×10^{-3}$	$7.74×10^{-3}±2.83×10^{-3}$	$3.96×10^{-3}±2.91×10^{-3}$
TG 54:2|TG 18:0_18:1_18:1	$7.45×10^{-3}±3.31×10^{-3}$	$1.17×10^{-2}±3.58×10^{-3}$	$8.20×10^{-3}±1.93×10^{-3}$	$3.00×10^{-3}±1.43×10^{-3}$
TG 54:1|TG 18:0_18:0_18:1	$1.87×10^{-3}±3.44×10^{-4}$	$1.94×10^{-3}±9.87×10^{-4}$	$2.07×10^{-3}±1.05×10^{-3}$	$1.17×10^{-3}±7.18×10^{-4}$
TG 54:1|TG 16:0_20:0_18:1	$1.64×10^{-3}±2.96×10^{-4}$	$1.80×10^{-3}±9.28×10^{-4}$	$1.79×10^{-3}±9.19×10^{-4}$	$9.73×10^{-4}±5.85×10^{-4}$
TG 53:5|TG 17:1_18:2_18:2	$3.19×10^{-5}±2.32×10^{-5}$	$1.80×10^{-4}±1.73×10^{-4}$	$3.24×10^{-5}±1.45×10^{-5}$	$1.12×10^{-5}±8.47×10^{-6}$
TG 53:4|TG 17:0_18:2_18:2	$2.88×10^{-4}±1.06×10^{-4}$	$8.80×10^{-4}±7.21×10^{-4}$	$2.57×10^{-4}±8.78×10^{-5}$	$7.45×10^{-5}±5.87×10^{-5}$
TG 53:2|TG 17:0_18:1_18:1	$1.78×10^{-3}±1.35×10^{-3}$	$3.04×10^{-3}±1.18×10^{-3}$	$2.74×10^{-3}±1.15×10^{-3}$	$3.89×10^{-4}±2.55×10^{-4}$
TG 53:2|TG 16:0_18:1_19:1	$1.60×10^{-3}±7.23×10^{-4}$	$2.09×10^{-3}±8.17×10^{-4}$	$1.47×10^{-3}±7.78×10^{-4}$	$5.42×10^{-4}±3.72×10^{-4}$
TG 53:1|TG 16:0_19:0_18:1	$2.30×10^{-4}±2.17×10^{-4}$	$6.45×10^{-4}±3.44×10^{-4}$	$3.37×10^{-4}±2.06×10^{-4}$	$9.56×10^{-5}±6.56×10^{-5}$
TG 52:6|TG 16:1_18:2_18:3	$1.31×10^{-4}±1.03×10^{-4}$	$2.61×10^{-4}±2.22×10^{-4}$	$1.00×10^{-4}±6.92×10^{-5}$	$2.56×10^{-5}±1.87×10^{-5}$
TG 52:5|TG 16:0_16:0_20:5	$2.39×10^{-5}±2.00×10^{-5}$	$3.07×10^{-5}±1.89×10^{-5}$	$1.58×10^{-5}±7.56×10^{-6}$	$2.97×10^{-6}±2.68×10^{-6}$
TG 52:4|TG 16:0_18:1_18:3	$1.68×10^{-2}±8.78×10^{-3}$	$1.59×10^{-2}±8.05×10^{-3}$	$9.93×10^{-3}±1.75×10^{-3}$	$3.26×10^{-3}±2.11×10^{-3}$
TG 52:3|TG 16:0_18:1_18:2	$2.53×10^{-2}±1.30×10^{-2}$	$2.53×10^{-2}±1.08×10^{-2}$	$2.39×10^{-2}±5.54×10^{-3}$	$8.98×10^{-3}±4.59×10^{-3}$
TG 52:2|TG 16:0_18:1_18:1	$2.44×10^{-2}±9.46×10^{-3}$	$2.82×10^{-2}±7.83×10^{-3}$	$2.62×10^{-2}±6.15×10^{-3}$	$1.07×10^{-2}±7.30×10^{-3}$
TG 52:1|TG 16:0_18:0_18:1	$4.15×10^{-3}±1.87×10^{-3}$	$7.51×10^{-3}±2.44×10^{-3}$	$4.97×10^{-3}±2.41×10^{-3}$	$2.06×10^{-3}±1.22×10^{-3}$
TG 51:4|TG 16:1_17:1_18:2	$4.73×10^{-5}±4.51×10^{-5}$	$2.42×10^{-4}±1.94×10^{-4}$	$4.13×10^{-5}±2.56×10^{-5}$	$3.38×10^{-6}±5.26×10^{-6}$
TG 51:4|TG 15:0_18:2_18:2	$6.72×10^{-5}±4.71×10^{-5}$	$2.43×10^{-4}±2.14×10^{-4}$	$5.31×10^{-5}±2.29×10^{-5}$	$1.28×10^{-5}±1.15×10^{-5}$
TG 51:2|TG 15:0_18:1_18:1	$8.95×10^{-4}±4.76×10^{-4}$	$1.64×10^{-3}±7.70×10^{-4}$	$1.02×10^{-3}±4.14×10^{-4}$	$2.35×10^{-4}±1.78×10^{-4}$

TG 51:1|TG 16:0_17:0_18:1	$2.42\times10^{-4}\pm1.01\times10^{-4}$	$1.05\times10^{-3}\pm6.38\times10^{-4}$	$3.78\times10^{-4}\pm2.57\times10^{-4}$	$9.49\times10^{-5}\pm8.63\times10^{-5}$
TG 51:0|TG 17:0_17:0_17:0	$1.31\times10^{-5}\pm1.36\times10^{-5}$	$1.21\times10^{-4}\pm8.44\times10^{-5}$	$2.69\times10^{-5}\pm2.54\times10^{-5}$	$1.84\times10^{-5}\pm2.23\times10^{-5}$
TG 51:0|TG 16:0_17:0_18:0	$4.08\times10^{-7}\pm8.17\times10^{-7}$	$1.64\times10^{-4}\pm1.68\times10^{-4}$	$1.26\times10^{-5}\pm2.23\times10^{-5}$	$9.59\times10^{-6}\pm1.92\times10^{-5}$
TG 51:0|TG 16:0_16:0_19:0	$2.43\times10^{-6}\pm4.84\times10^{-6}$	$1.03\times10^{-4}\pm1.06\times10^{-4}$	$1.36\times10^{-5}\pm2.72\times10^{-5}$	$2.19\times10^{-5}\pm3.14\times10^{-5}$
TG 50:5|TG 16:1_16:1_18:3	$2.83\times10^{-5}\pm2.36\times10^{-5}$	$2.47\times10^{-4}\pm2.74\times10^{-4}$	$1.52\times10^{-5}\pm1.02\times10^{-5}$	$7.41\times10^{-6}\pm1.07\times10^{-5}$
TG 50:4|TG 16:0_16:1_18:3	$2.67\times10^{-4}\pm2.32\times10^{-4}$	$1.18\times10^{-3}\pm1.17\times10^{-3}$	$1.36\times10^{-4}\pm1.05\times10^{-4}$	$7.66\times10^{-5}\pm1.00\times10^{-4}$
TG 50:4|TG 14:0_18:2_18:2	$3.93\times10^{-4}\pm2.35\times10^{-4}$	$1.70\times10^{-3}\pm1.78\times10^{-3}$	$2.31\times10^{-4}\pm9.75\times10^{-5}$	$9.80\times10^{-5}\pm9.62\times10^{-5}$
TG 50:3|TG 16:0_16:0_18:3	$2.44\times10^{-3}\pm2.15\times10^{-3}$	$5.21\times10^{-3}\pm3.79\times10^{-3}$	$1.06\times10^{-3}\pm3.63\times10^{-4}$	$5.47\times10^{-4}\pm3.66\times10^{-4}$
TG 50:1|TG 16:0_16:0_18:1	$2.33\times10^{-3}\pm5.85\times10^{-4}$	$7.65\times10^{-3}\pm4.88\times10^{-3}$	$2.46\times10^{-3}\pm7.12\times10^{-4}$	$1.45\times10^{-3}\pm1.09\times10^{-3}$
TG 50:0|TG 15:0_17:0_18:0	$3.74\times10^{-4}\pm5.34\times10^{-5}$	$1.29\times10^{-3}\pm8.18\times10^{-4}$	$5.10\times10^{-4}\pm2.05\times10^{-4}$	$4.52\times10^{-4}\pm3.91\times10^{-4}$
TG 49:3|TG 15:0_16:1_18:2	-	$1.24\times10^{-4}\pm1.05\times10^{-4}$	$1.04\times10^{-5}\pm1.28\times10^{-5}$	-
TG 49:1|TG 16:0_16:0_17:1	-	$2.21\times10^{-4}\pm2.36\times10^{-4}$	-	-
TG 49:0|TG 16:0_16:0_17:0	-	$9.51\times10^{-5}\pm1.41\times10^{-4}$	$1.01\times10^{-5}\pm2.02\times10^{-5}$	$1.22\times10^{-5}\pm2.44\times10^{-5}$
TG 48:3|TG 16:1_16:1_16:1	$5.60\times10^{-5}\pm4.78\times10^{-5}$	$6.31\times10^{-4}\pm6.16\times10^{-4}$	$7.89\times10^{-6}\pm1.04\times10^{-5}$	$2.16\times10^{-5}\pm3.95\times10^{-5}$
TG 48:3|TG 14:0_16:1_18:2	$6.67\times10^{-5}\pm4.03\times10^{-5}$	$7.28\times10^{-4}\pm6.36\times10^{-4}$	$5.08\times10^{-5}\pm6.41\times10^{-5}$	$3.27\times10^{-5}\pm3.58\times10^{-5}$
TG 48:0|TG 16:0_16:0_16:0	-	$1.37\times10^{-3}\pm1.64\times10^{-3}$	-	$7.00\times10^{-5}\pm1.40\times10^{-4}$
TG 47:2|TG 14:0_15:0_18:2	$1.64\times10^{-6}\pm3.28\times10^{-6}$	$8.49\times10^{-5}\pm5.85\times10^{-5}$	$4.80\times10^{-5}\pm4.18\times10^{-5}$	$9.06\times10^{-9}\pm1.81\times10^{-8}$
TG 46:3|TG 14:1_16:1_16:1	$1.27\times10^{-7}\pm2.54\times10^{-7}$	$1.01\times10^{-4}\pm1.12\times10^{-4}$	$8.88\times10^{-7}\pm1.78\times10^{-6}$	-
TG 46:3|TG 14:0_14:0_18:3	-	$1.09\times10^{-4}\pm1.26\times10^{-4}$	$1.08\times10^{-6}\pm2.16\times10^{-6}$	-
TG 46:2|TG 14:0_14:0_18:2	-	$3.41\times10^{-4}\pm3.27\times10^{-4}$	$1.62\times10^{-5}\pm1.69\times10^{-5}$	$7.13\times10^{-6}\pm1.43\times10^{-5}$
TG 44:1|TG 14:0_14:0_16:1	-	$1.07\times10^{-4}\pm1.25\times10^{-4}$	-	-
TG 40:0|TG 8:0_16:0_16:0	-	$6.54\times10^{-6}\pm8.21\times10^{-6}$	$2.03\times10^{-6}\pm3.99\times10^{-6}$	-
TG 36:0|TG 12:0_12:0_12:0	-	$1.94\times10^{-5}\pm1.63\times10^{-5}$	$1.01\times10^{-5}\pm8.36\times10^{-6}$	-
TG 24:0|TG 8:0_8:0_8:0	$6.49\times10^{-6}\pm1.78\times10^{-6}$	$7.41\times10^{-6}\pm2.35\times10^{-6}$	$6.63\times10^{-6}\pm2.36\times10^{-6}$	$5.69\times10^{-6}\pm1.44\times10^{-6}$
SPB 18:1;2O	$1.82\times10^{-4}\pm4.41\times10^{-5}$	$1.26\times10^{-4}\pm2.18\times10^{-5}$	$1.36\times10^{-4}\pm5.24\times10^{-5}$	$1.46\times10^{-4}\pm2.07\times10^{-5}$
SM 60:4;2O	$9.22\times10^{-6}\pm3.57\times10^{-6}$	$9.51\times10^{-6}\pm7.94\times10^{-6}$	$1.28\times10^{-5}\pm6.04\times10^{-6}$	$9.54\times10^{-6}\pm3.54\times10^{-6}$

SM 60:3;2O	$2.18\times10^{-5}\pm7.99\times10^{-6}$	$2.15\times10^{-5}\pm1.65\times10^{-5}$	$2.67\times10^{-5}\pm9.70\times10^{-6}$	$3.34\times10^{-5}\pm1.13\times10^{-5}$
SM 60:2;2O	$1.74\times10^{-5}\pm2.92\times10^{-6}$	$1.53\times10^{-5}\pm8.39\times10^{-6}$	$1.68\times10^{-5}\pm4.97\times10^{-6}$	$2.17\times10^{-5}\pm5.97\times10^{-6}$
SM 59:3;2O	$4.30\times10^{-6}\pm9.30\times10^{-7}$	$4.80\times10^{-6}\pm2.64\times10^{-6}$	$4.86\times10^{-6}\pm9.58\times10^{-7}$	$4.73\times10^{-6}\pm1.63\times10^{-6}$
SM 59:2;2O	$7.17\times10^{-6}\pm1.23\times10^{-6}$	$1.14\times10^{-5}\pm5.34\times10^{-6}$	$1.18\times10^{-5}\pm4.09\times10^{-6}$	$9.31\times10^{-6}\pm2.24\times10^{-6}$
SM 58:3;2O	$2.27\times10^{-5}\pm7.26\times10^{-6}$	$1.22\times10^{-5}\pm9.80\times10^{-6}$	$1.84\times10^{-5}\pm6.59\times10^{-6}$	$5.22\times10^{-5}\pm1.15\times10^{-5}$
SM 58:2;2O	$2.23\times10^{-5}\pm7.65\times10^{-6}$	$2.06\times10^{-5}\pm1.53\times10^{-5}$	$2.30\times10^{-5}\pm7.47\times10^{-6}$	$3.83\times10^{-5}\pm7.28\times10^{-6}$
SM 57:2;2O	$8.06\times10^{-6}\pm2.01\times10^{-6}$	$9.77\times10^{-6}\pm5.62\times10^{-6}$	$1.06\times10^{-5}\pm2.71\times10^{-6}$	$1.07\times10^{-5}\pm1.41\times10^{-6}$
SM 56:2;2O	$1.99\times10^{-5}\pm8.06\times10^{-6}$	$1.32\times10^{-5}\pm8.43\times10^{-6}$	$1.83\times10^{-5}\pm1.01\times10^{-5}$	$2.70\times10^{-5}\pm4.53\times10^{-6}$
SM 54:2;2O	$8.35\times10^{-6}\pm6.35\times10^{-6}$	$7.74\times10^{-6}\pm3.52\times10^{-6}$	$1.20\times10^{-5}\pm6.44\times10^{-6}$	$8.24\times10^{-6}\pm3.37\times10^{-6}$
SM 52:9;2O	$3.93\times10^{-6}\pm5.09\times10^{-6}$	$1.76\times10^{-6}\pm6.11\times10^{-7}$	$1.26\times10^{-6}\pm1.43\times10^{-6}$	$6.92\times10^{-7}\pm1.03\times10^{-6}$
SM 52:2;2O	$1.25\times10^{-5}\pm3.36\times10^{-6}$	$1.30\times10^{-5}\pm6.44\times10^{-6}$	$1.17\times10^{-5}\pm2.67\times10^{-6}$	$1.42\times10^{-5}\pm2.38\times10^{-6}$
SM 50:11;2O	$1.19\times10^{-5}\pm3.62\times10^{-6}$	$5.28\times10^{-6}\pm2.55\times10^{-6}$	$9.13\times10^{-6}\pm3.53\times10^{-6}$	$4.91\times10^{-6}\pm3.19\times10^{-6}$
SM 48:9;2O	$1.74\times10^{-5}\pm7.50\times10^{-6}$	$1.30\times10^{-5}\pm2.29\times10^{-6}$	$1.28\times10^{-5}\pm8.73\times10^{-6}$	$7.31\times10^{-6}\pm3.34\times10^{-6}$
SM 44:6;2O	$4.24\times10^{-5}\pm1.35\times10^{-5}$	$2.89\times10^{-5}\pm7.08\times10^{-6}$	$3.68\times10^{-5}\pm1.84\times10^{-5}$	$3.07\times10^{-5}\pm1.23\times10^{-5}$
SM 44:2;2O|SM 18:1;2O/26:1	$7.79\times10^{-5}\pm1.38\times10^{-5}$	$1.11\times10^{-4}\pm1.33\times10^{-5}$	$1.21\times10^{-4}\pm7.97\times10^{-5}$	$7.24\times10^{-5}\pm2.89\times10^{-5}$
SM 44:1;2O|SM 18:1;2O/26:0	$7.86\times10^{-5}\pm3.70\times10^{-5}$	$1.28\times10^{-4}\pm3.13\times10^{-5}$	$9.74\times10^{-5}\pm3.87\times10^{-5}$	$1.04\times10^{-4}\pm3.96\times10^{-5}$
SM 43:4;2O	$1.04\times10^{-4}\pm4.04\times10^{-5}$	$9.51\times10^{-5}\pm3.48\times10^{-5}$	$1.45\times10^{-4}\pm3.64\times10^{-5}$	$1.00\times10^{-4}\pm2.69\times10^{-5}$
SM 43:2;2O|SM 19:1;2O/24:1	$2.17\times10^{-4}\pm2.96\times10^{-5}$	$2.70\times10^{-4}\pm5.41\times10^{-5}$	$2.48\times10^{-4}\pm7.23\times10^{-5}$	$1.97\times10^{-4}\pm5.15\times10^{-5}$
SM 43:1;2O|SM 18:1;2O/25:0	$4.18\times10^{-4}\pm7.01\times10^{-5}$	$3.94\times10^{-4}\pm4.97\times10^{-5}$	$4.31\times10^{-4}\pm9.74\times10^{-5}$	$4.14\times10^{-4}\pm7.56\times10^{-5}$
SM 42:3;2O|SM 18:1;2O/24:2	$1.86\times10^{-3}\pm2.63\times10^{-4}$	$1.31\times10^{-3}\pm2.73\times10^{-4}$	$1.39\times10^{-3}\pm4.12\times10^{-4}$	$1.13\times10^{-3}\pm3.22\times10^{-4}$
SM 42:2;2O|SM 18:1;2O/24:1	$1.56\times10^{-2}\pm4.31\times10^{-3}$	$1.29\times10^{-2}\pm2.92\times10^{-3}$	$1.38\times10^{-2}\pm2.01\times10^{-3}$	$9.63\times10^{-3}\pm1.75\times10^{-3}$
SM 42:1;2O|SM 18:1;2O/24:0	$8.97\times10^{-3}\pm1.01\times10^{-3}$	$1.04\times10^{-2}\pm5.65\times10^{-3}$	$1.03\times10^{-2}\pm2.16\times10^{-3}$	$7.60\times10^{-3}\pm6.13\times10^{-4}$
SM 42:1;2O	$1.78\times10^{-4}\pm3.82\times10^{-5}$	$1.41\times10^{-4}\pm3.68\times10^{-5}$	$1.63\times10^{-4}\pm5.22\times10^{-5}$	$1.76\times10^{-4}\pm2.28\times10^{-5}$
SM 42:0;2O	$1.05\times10^{-4}\pm2.29\times10^{-5}$	$1.24\times10^{-4}\pm3.87\times10^{-5}$	$1.11\times10^{-4}\pm2.37\times10^{-5}$	$9.57\times10^{-5}\pm1.96\times10^{-5}$
SM 41:2;2O|SM 17:1;2O/24:1	$9.61\times10^{-4}\pm1.07\times10^{-4}$	$9.94\times10^{-4}\pm2.29\times10^{-4}$	$1.22\times10^{-3}\pm4.28\times10^{-4}$	$8.44\times10^{-4}\pm1.80\times10^{-4}$
SM 41:2;2O	$9.12\times10^{-4}\pm1.33\times10^{-4}$	$6.49\times10^{-4}\pm1.44\times10^{-4}$	$8.23\times10^{-4}\pm2.04\times10^{-4}$	$8.69\times10^{-4}\pm1.25\times10^{-4}$

SM 41:1;2O|SM 18:1;2O/23:0	$6.60\times10^{-3}\pm1.61\times10^{-3}$	$8.23\times10^{-3}\pm2.84\times10^{-3}$	$7.28\times10^{-3}\pm1.24\times10^{-3}$	$5.99\times10^{-3}\pm1.68\times10^{-3}$
SM 41:0;2O	$2.73\times10^{-5}\pm6.65\times10^{-6}$	$3.30\times10^{-5}\pm1.26\times10^{-5}$	$2.96\times10^{-5}\pm6.36\times10^{-6}$	$2.75\times10^{-5}\pm7.94\times10^{-6}$
SM 40:2;2O|SM 18:2;2O/22:0	$1.01\times10^{-3}\pm2.45\times10^{-4}$	$8.59\times10^{-4}\pm1.95\times10^{-4}$	$1.04\times10^{-3}\pm3.29\times10^{-4}$	$9.15\times10^{-4}\pm9.14\times10^{-5}$
SM 40:2;2O|SM 18:1;2O/22:1	$9.35\times10^{-4}\pm2.50\times10^{-4}$	$8.31\times10^{-4}\pm1.89\times10^{-4}$	$8.61\times10^{-4}\pm2.75\times10^{-4}$	$7.64\times10^{-4}\pm9.28\times10^{-5}$
SM 40:1;2O|SM 18:1;2O/22:0	$1.72\times10^{-2}\pm3.72\times10^{-3}$	$1.06\times10^{-2}\pm2.25\times10^{-3}$	$1.46\times10^{-2}\pm4.44\times10^{-3}$	$1.87\times10^{-2}\pm1.68\times10^{-3}$
SM 40:0;2O	$9.36\times10^{-5}\pm2.50\times10^{-5}$	$1.33\times10^{-4}\pm4.07\times10^{-5}$	$1.03\times10^{-4}\pm2.34\times10^{-5}$	$1.24\times10^{-4}\pm2.90\times10^{-5}$
SM 39:1;2O|SM 17:1;2O/22:0	$8.68\times10^{-4}\pm1.86\times10^{-4}$	$1.00\times10^{-3}\pm2.62\times10^{-4}$	$9.62\times10^{-4}\pm1.49\times10^{-4}$	$1.02\times10^{-3}\pm1.36\times10^{-4}$
SM 39:1;2O	$1.12\times10^{-3}\pm1.75\times10^{-4}$	$1.08\times10^{-3}\pm3.50\times10^{-4}$	$1.14\times10^{-3}\pm1.88\times10^{-4}$	$1.15\times10^{-3}\pm2.31\times10^{-4}$
SM 39:0;2O	$9.28\times10^{-5}\pm2.58\times10^{-5}$	$1.18\times10^{-4}\pm3.62\times10^{-5}$	$1.15\times10^{-4}\pm3.42\times10^{-5}$	$1.47\times10^{-4}\pm4.04\times10^{-5}$
SM 38:2;2O	$4.54\times10^{-5}\pm1.04\times10^{-5}$	$3.76\times10^{-5}\pm9.36\times10^{-6}$	$3.69\times10^{-5}\pm1.66\times10^{-5}$	$3.26\times10^{-5}\pm5.80\times10^{-6}$
SM 38:1;2O|SM 18:1;2O/20:0	$2.28\times10^{-3}\pm5.79\times10^{-4}$	$1.87\times10^{-3}\pm3.48\times10^{-4}$	$1.78\times10^{-3}\pm3.44\times10^{-4}$	$2.28\times10^{-3}\pm5.72\times10^{-4}$
SM 38:0;2O	$3.08\times10^{-4}\pm8.93\times10^{-5}$	$3.37\times10^{-4}\pm3.91\times10^{-5}$	$3.06\times10^{-4}\pm1.54\times10^{-4}$	$2.87\times10^{-4}\pm5.88\times10^{-5}$
SM 36:2;2O|SM 18:2;2O/18:0	$1.17\times10^{-4}\pm3.08\times10^{-5}$	$1.03\times10^{-4}\pm9.43\times10^{-6}$	$1.08\times10^{-4}\pm4.84\times10^{-5}$	$7.80\times10^{-5}\pm1.26\times10^{-5}$
SM 36:1;2O|SM 18:1;2O/18:0	$4.02\times10^{-3}\pm9.93\times10^{-4}$	$3.79\times10^{-3}\pm5.82\times10^{-4}$	$3.60\times10^{-3}\pm1.41\times10^{-3}$	$2.56\times10^{-3}\pm5.93\times10^{-4}$
SM 36:1;2O|SM 16:1;2O/20:0	$4.02\times10^{-3}\pm1.19\times10^{-3}$	$3.41\times10^{-3}\pm7.18\times10^{-4}$	$3.32\times10^{-3}\pm1.29\times10^{-3}$	$2.86\times10^{-3}\pm4.27\times10^{-4}$
SM 36:0;2O	$1.89\times10^{-4}\pm8.19\times10^{-5}$	$2.24\times10^{-4}\pm7.00\times10^{-5}$	$1.52\times10^{-4}\pm6.97\times10^{-5}$	$1.77\times10^{-4}\pm9.56\times10^{-5}$
SM 35:5;2O	$2.35\times10^{-5}\pm1.17\times10^{-5}$	$1.90\times10^{-5}\pm1.13\times10^{-5}$	$2.58\times10^{-5}\pm1.04\times10^{-5}$	$2.06\times10^{-5}\pm9.33\times10^{-6}$
SM 34:2;2O|SM 18:2;2O/16:0	$1.96\times10^{-4}\pm2.86\times10^{-5}$	$1.91\times10^{-4}\pm4.80\times10^{-5}$	$1.85\times10^{-4}\pm5.76\times10^{-5}$	$1.40\times10^{-4}\pm1.40\times10^{-5}$
SM 34:2;2O	$1.23\times10^{-4}\pm2.93\times10^{-5}$	$1.38\times10^{-4}\pm3.21\times10^{-5}$	$1.01\times10^{-4}\pm1.60\times10^{-5}$	$7.65\times10^{-5}\pm1.13\times10^{-5}$
SM 34:1;2O|SM 18:1;2O/16:0	$1.27\times10^{-2}\pm1.22\times10^{-3}$	$1.07\times10^{-2}\pm1.45\times10^{-3}$	$1.16\times10^{-2}\pm3.11\times10^{-3}$	$9.90\times10^{-3}\pm1.67\times10^{-3}$
SM 34:1;2O	$1.56\times10^{-4}\pm2.10\times10^{-5}$	$1.58\times10^{-4}\pm4.06\times10^{-5}$	$1.52\times10^{-4}\pm2.34\times10^{-5}$	$1.16\times10^{-4}\pm2.97\times10^{-5}$
SM 34:0;2O	$1.01\times10^{-3}\pm1.72\times10^{-4}$	$8.83\times10^{-4}\pm6.44\times10^{-5}$	$1.05\times10^{-3}\pm3.57\times10^{-4}$	$8.23\times10^{-4}\pm2.43\times10^{-4}$
SM 33:1;2O|SM 17:1;2O/16:0	$2.78\times10^{-4}\pm5.26\times10^{-5}$	$3.27\times10^{-4}\pm5.36\times10^{-5}$	$2.88\times10^{-4}\pm4.10\times10^{-5}$	$2.49\times10^{-4}\pm8.97\times10^{-5}$
SM 33:1;2O	$1.05\times10^{-5}\pm3.53\times10^{-6}$	$8.08\times10^{-6}\pm2.38\times10^{-6}$	$1.16\times10^{-5}\pm3.75\times10^{-6}$	$1.05\times10^{-5}\pm3.89\times10^{-6}$
SM 32:1;2O|SM 18:1;2O/14:0	$6.94\times10^{-5}\pm1.23\times10^{-5}$	$7.26\times10^{-5}\pm1.27\times10^{-5}$	$6.16\times10^{-5}\pm2.01\times10^{-5}$	$5.28\times10^{-5}\pm1.23\times10^{-5}$
SM 32:1;2O|SM 16:1;2O/16:0	$7.04\times10^{-5}\pm1.07\times10^{-5}$	$6.49\times10^{-5}\pm1.06\times10^{-5}$	$5.92\times10^{-5}\pm1.29\times10^{-5}$	$5.48\times10^{-5}\pm1.09\times10^{-5}$

PS 42:9	$2.86\times10^{-5}\pm1.49\times10^{-5}$	$1.53\times10^{-5}\pm4.38\times10^{-6}$	$2.82\times10^{-5}\pm8.44\times10^{-6}$	$2.24\times10^{-5}\pm8.70\times10^{-6}$
PS 40:6	$3.86\times10^{-4}\pm1.23\times10^{-4}$	$3.60\times10^{-4}\pm1.53\times10^{-4}$	$5.58\times10^{-4}\pm2.29\times10^{-4}$	$4.69\times10^{-4}\pm3.34\times10^{-4}$
PS 40:1	$6.61\times10^{-6}\pm1.59\times10^{-6}$	$1.15\times10^{-5}\pm3.69\times10^{-6}$	$8.28\times10^{-6}\pm3.17\times10^{-6}$	$5.50\times10^{-6}\pm1.82\times10^{-6}$
PS 36:2	$6.52\times10^{-5}\pm1.75\times10^{-5}$	$4.64\times10^{-5}\pm1.14\times10^{-5}$	$6.99\times10^{-5}\pm1.90\times10^{-5}$	$6.68\times10^{-5}\pm3.21\times10^{-5}$
PS 36:1	$2.40\times10^{-4}\pm7.06\times10^{-5}$	$2.71\times10^{-4}\pm1.05\times10^{-4}$	$2.29\times10^{-4}\pm8.61\times10^{-5}$	$2.65\times10^{-4}\pm5.35\times10^{-5}$
PMeOH 38:6|PMeOH 16:0_22:6	$2.41\times10^{-6}\pm1.49\times10^{-6}$	$5.84\times10^{-7}\pm1.84\times10^{-7}$	$1.10\times10^{-6}\pm8.64\times10^{-7}$	$3.24\times10^{-6}\pm1.40\times10^{-6}$
PMeOH 36:4|PMeOH 16:0_20:4	$2.35\times10^{-6}\pm1.02\times10^{-6}$	$3.31\times10^{-6}\pm1.09\times10^{-6}$	$2.41\times10^{-6}\pm9.60\times10^{-7}$	$4.49\times10^{-6}\pm1.19\times10^{-6}$
PMeOH 36:2|PMeOH 18:0_18:2	$1.88\times10^{-6}\pm1.45\times10^{-7}$	$3.40\times10^{-6}\pm4.73\times10^{-7}$	$2.76\times10^{-6}\pm1.27\times10^{-6}$	$2.89\times10^{-6}\pm7.06\times10^{-7}$
PI 42:9|PI 20:3_22:6	$1.07\times10^{-6}\pm6.27\times10^{-7}$	$2.66\times10^{-7}\pm2.32\times10^{-7}$	$4.37\times10^{-7}\pm3.26\times10^{-7}$	$1.58\times10^{-7}\pm7.44\times10^{-8}$
PI 42:10|PI 20:4_22:6	$4.92\times10^{-6}\pm1.96\times10^{-6}$	$1.92\times10^{-6}\pm1.22\times10^{-6}$	$2.80\times10^{-6}\pm1.22\times10^{-6}$	$1.03\times10^{-6}\pm3.30\times10^{-7}$
PI 40:8|PI 20:4_20:4	$2.98\times10^{-5}\pm1.34\times10^{-5}$	$1.87\times10^{-5}\pm5.04\times10^{-6}$	$2.64\times10^{-5}\pm5.25\times10^{-6}$	$1.69\times10^{-5}\pm7.32\times10^{-6}$
PI 40:7|PI 18:1_22:6	$1.36\times10^{-5}\pm2.21\times10^{-6}$	$9.22\times10^{-6}\pm3.16\times10^{-6}$	$1.21\times10^{-5}\pm3.73\times10^{-6}$	$1.99\times10^{-5}\pm7.31\times10^{-6}$
PI 40:6|PI 20:2_20:4	$2.89\times10^{-6}\pm7.32\times10^{-7}$	$1.60\times10^{-6}\pm2.23\times10^{-7}$	$2.17\times10^{-6}\pm9.18\times10^{-7}$	$3.48\times10^{-6}\pm5.60\times10^{-7}$
PI 40:6|PI 18:1_22:5	$7.56\times10^{-6}\pm2.25\times10^{-6}$	$6.72\times10^{-6}\pm4.51\times10^{-6}$	$1.41\times10^{-5}\pm5.82\times10^{-6}$	$4.51\times10^{-6}\pm1.15\times10^{-6}$
PI 40:6|PI 18:0_22:6	$1.45\times10^{-4}\pm4.03\times10^{-5}$	$6.98\times10^{-5}\pm2.97\times10^{-5}$	$1.26\times10^{-4}\pm1.40\times10^{-5}$	$1.92\times10^{-4}\pm4.78\times10^{-5}$
PI 40:5|PI 18:0_22:5	$4.62\times10^{-5}\pm7.33\times10^{-6}$	$1.76\times10^{-5}\pm8.85\times10^{-6}$	$4.33\times10^{-5}\pm1.33\times10^{-5}$	$4.47\times10^{-5}\pm1.24\times10^{-5}$
PI 40:5	$2.79\times10^{-4}\pm6.26\times10^{-5}$	$2.91\times10^{-4}\pm9.95\times10^{-5}$	$2.35\times10^{-4}\pm4.36\times10^{-5}$	$2.90\times10^{-4}\pm6.71\times10^{-5}$
PI 40:4|PI 20:1_20:3	$1.75\times10^{-5}\pm2.21\times10^{-6}$	$3.33\times10^{-6}\pm8.63\times10^{-7}$	$1.14\times10^{-5}\pm3.93\times10^{-6}$	$1.57\times10^{-5}\pm3.28\times10^{-6}$
PI 40:4|PI 20:0_20:4	$5.02\times10^{-6}\pm1.09\times10^{-6}$	$3.77\times10^{-5}\pm1.34\times10^{-5}$	$1.39\times10^{-5}\pm4.25\times10^{-6}$	$4.67\times10^{-6}\pm2.30\times10^{-6}$
PI 40:4|PI 18:0_22:4	$3.88\times10^{-5}\pm4.56\times10^{-6}$	$1.64\times10^{-5}\pm6.32\times10^{-6}$	$3.67\times10^{-5}\pm6.45\times10^{-6}$	$2.35\times10^{-5}\pm6.83\times10^{-6}$
PI 40:3|PI 20:0_20:3	$1.80\times10^{-7}\pm1.91\times10^{-7}$	$2.29\times10^{-6}\pm1.17\times10^{-6}$	$1.69\times10^{-6}\pm6.70\times10^{-7}$	$2.81\times10^{-7}\pm2.28\times10^{-7}$
PI 40:3|PI 18:0_22:3	$1.70\times10^{-5}\pm4.38\times10^{-6}$	$4.13\times10^{-6}\pm2.78\times10^{-6}$	$1.14\times10^{-5}\pm3.79\times10^{-6}$	$2.55\times10^{-5}\pm2.58\times10^{-5}$
PI 40:2|PI 18:0_22:2	$1.45\times10^{-6}\pm2.19\times10^{-7}$	$1.23\times10^{-6}\pm6.55\times10^{-7}$	$1.87\times10^{-6}\pm7.99\times10^{-7}$	$1.50\times10^{-6}\pm5.23\times10^{-7}$
PI 39:6	$4.70\times10^{-7}\pm6.35\times10^{-7}$	$6.31\times10^{-7}\pm7.68\times10^{-7}$	$1.88\times10^{-6}\pm1.20\times10^{-6}$	$1.40\times10^{-6}\pm7.71\times10^{-7}$
PI 39:4|PI 19:0_20:4	$1.81\times10^{-5}\pm3.31\times10^{-6}$	$2.69\times10^{-5}\pm5.71\times10^{-6}$	$2.48\times10^{-5}\pm6.62\times10^{-6}$	$1.87\times10^{-5}\pm2.20\times10^{-6}$
PI 39:4	$5.44\times10^{-6}\pm1.32\times10^{-6}$	$6.81\times10^{-6}\pm4.60\times10^{-6}$	$5.16\times10^{-6}\pm3.14\times10^{-6}$	$5.51\times10^{-6}\pm1.27\times10^{-6}$

PI 39:3|PI 19:0_20:3	$1.57\times10^{-6}\pm7.35\times10^{-8}$	$9.54\times10^{-7}\pm5.11\times10^{-7}$	$1.41\times10^{-6}\pm2.26\times10^{-7}$	$1.96\times10^{-6}\pm2.88\times10^{-7}$
PI 38:6|PI 18:2_20:4	$1.61\times10^{-5}\pm3.88\times10^{-6}$	$7.93\times10^{-6}\pm2.59\times10^{-6}$	$1.30\times10^{-5}\pm2.88\times10^{-6}$	$1.43\times10^{-5}\pm1.87\times10^{-6}$
PI 38:6|PI 16:0_22:6	$2.74\times10^{-5}\pm5.11\times10^{-6}$	$2.17\times10^{-5}\pm3.55\times10^{-6}$	$1.97\times10^{-5}\pm2.76\times10^{-6}$	$3.67\times10^{-5}\pm1.06\times10^{-5}$
PI 38:5|PI 18:2_20:3	$4.62\times10^{-6}\pm1.25\times10^{-6}$	$2.54\times10^{-6}\pm7.42\times10^{-7}$	$2.80\times10^{-6}\pm6.02\times10^{-7}$	$4.13\times10^{-6}\pm1.21\times10^{-6}$
PI 38:5|PI 18:1_20:4	$1.45\times10^{-6}\pm7.97\times10^{-7}$	$8.58\times10^{-7}\pm5.66\times10^{-7}$	$4.65\times10^{-7}\pm4.71\times10^{-7}$	$1.78\times10^{-6}\pm3.61\times10^{-7}$
PI 38:5|PI 18:0_20:5	$2.10\times10^{-5}\pm5.68\times10^{-6}$	$4.01\times10^{-5}\pm1.86\times10^{-5}$	$2.93\times10^{-5}\pm7.91\times10^{-6}$	$2.36\times10^{-5}\pm5.08\times10^{-6}$
PI 38:5|PI 16:0_22:5	$4.83\times10^{-5}\pm1.05\times10^{-5}$	$3.15\times10^{-5}\pm5.09\times10^{-6}$	$4.60\times10^{-5}\pm9.32\times10^{-6}$	$6.19\times10^{-5}\pm9.85\times10^{-6}$
PI 38:5;O|PI 18:1_20:4;O	$1.44\times10^{-6}\pm9.76\times10^{-7}$	$3.25\times10^{-6}\pm4.25\times10^{-6}$	$3.67\times10^{-6}\pm4.35\times10^{-6}$	$6.84\times10^{-7}\pm4.73\times10^{-7}$
PI 38:5;O|PI 18:0_20:5;O	$1.39\times10^{-6}\pm9.83\times10^{-7}$	$8.90\times10^{-7}\pm4.92\times10^{-7}$	$1.63\times10^{-6}\pm1.13\times10^{-6}$	$5.04\times10^{-7}\pm1.73\times10^{-7}$
PI 38:5;3O|PI 18:0_20:5;3O	$1.09\times10^{-6}\pm2.13\times10^{-7}$	$2.93\times10^{-6}\pm1.23\times10^{-6}$	$2.75\times10^{-6}\pm2.21\times10^{-6}$	$9.91\times10^{-7}\pm3.64\times10^{-7}$
PI 38:5	$2.85\times10^{-4}\pm9.32\times10^{-5}$	$2.34\times10^{-4}\pm4.34\times10^{-5}$	$2.54\times10^{-4}\pm5.98\times10^{-5}$	$2.92\times10^{-4}\pm7.17\times10^{-5}$
PI 38:4|PI 18:1_20:3	$4.13\times10^{-5}\pm2.08\times10^{-5}$	$4.79\times10^{-5}\pm2.91\times10^{-5}$	$7.53\times10^{-5}\pm2.81\times10^{-5}$	$2.98\times10^{-5}\pm6.24\times10^{-6}$
PI 38:4|PI 18:0_20:4	$3.25\times10^{-3}\pm5.26\times10^{-4}$	$1.14\times10^{-3}\pm2.72\times10^{-4}$	$2.61\times10^{-3}\pm3.89\times10^{-4}$	$2.72\times10^{-3}\pm5.65\times10^{-4}$
PI 38:4;O|PI 18:0_20:4;O	$1.28\times10^{-5}\pm2.20\times10^{-6}$	$2.54\times10^{-5}\pm1.88\times10^{-5}$	$2.30\times10^{-5}\pm1.24\times10^{-5}$	$1.01\times10^{-5}\pm2.94\times10^{-6}$
PI 38:4;3O|PI 18:0_20:4;3O	$3.59\times10^{-6}\pm1.55\times10^{-6}$	$1.45\times10^{-5}\pm2.01\times10^{-5}$	$6.49\times10^{-6}\pm5.07\times10^{-6}$	$1.94\times10^{-6}\pm2.78\times10^{-7}$
PI 38:3|PI 18:0_20:3	$2.49\times10^{-4}\pm9.41\times10^{-5}$	$3.35\times10^{-3}\pm2.35\times10^{-3}$	$5.46\times10^{-3}\pm8.01\times10^{-3}$	$1.20\times10^{-5}\pm4.46\times10^{-6}$
PI 38:3;2O|PI 18:0_20:3;2O	$4.60\times10^{-7}\pm6.74\times10^{-8}$	$1.31\times10^{-6}\pm1.48\times10^{-6}$	$6.06\times10^{-7}\pm2.71\times10^{-7}$	$6.52\times10^{-7}\pm2.47\times10^{-7}$
PI 38:3	$7.03\times10^{-4}\pm1.22\times10^{-4}$	$4.45\times10^{-4}\pm2.51\times10^{-4}$	$5.70\times10^{-4}\pm9.69\times10^{-5}$	$6.70\times10^{-4}\pm7.06\times10^{-5}$
PI 38:2|PI 18:0_20:2	$1.41\times10^{-5}\pm4.89\times10^{-6}$	$1.19\times10^{-5}\pm6.63\times10^{-6}$	$7.64\times10^{-6}\pm3.12\times10^{-6}$	$2.88\times10^{-5}\pm1.29\times10^{-5}$
PI 38:1|PI 18:0_20:1	$3.92\times10^{-6}\pm7.38\times10^{-7}$	$3.44\times10^{-6}\pm1.85\times10^{-6}$	$6.15\times10^{-6}\pm3.34\times10^{-6}$	$4.11\times10^{-6}\pm1.18\times10^{-6}$
PI 37:5|PI 17:1_20:4	$1.28\times10^{-7}\pm8.15\times10^{-8}$	$1.27\times10^{-7}\pm6.53\times10^{-8}$	$5.99\times10^{-8}\pm3.40\times10^{-8}$	$2.24\times10^{-7}\pm1.02\times10^{-7}$
PI 37:4|PI 17:0_20:4	$1.68\times10^{-6}\pm6.12\times10^{-7}$	$1.05\times10^{-6}\pm4.03\times10^{-7}$	$8.85\times10^{-7}\pm3.73\times10^{-7}$	$2.63\times10^{-6}\pm4.84\times10^{-7}$
PI 37:3|PI 17:0_20:3	$8.50\times10^{-6}\pm1.98\times10^{-6}$	$5.18\times10^{-6}\pm2.35\times10^{-6}$	$1.11\times10^{-5}\pm3.36\times10^{-6}$	$5.76\times10^{-6}\pm1.94\times10^{-6}$
PI 37:2|PI 19:0_18:2	$7.84\times10^{-7}\pm1.81\times10^{-7}$	$6.09\times10^{-7}\pm3.64\times10^{-7}$	$1.14\times10^{-6}\pm5.24\times10^{-7}$	$7.68\times10^{-7}\pm1.67\times10^{-7}$
PI 36:5|PI 16:1_20:4	$1.71\times10^{-6}\pm4.67\times10^{-7}$	$1.16\times10^{-6}\pm2.96\times10^{-7}$	$1.62\times10^{-6}\pm4.04\times10^{-7}$	$1.86\times10^{-6}\pm3.40\times10^{-7}$
PI 36:5|PI 16:0_20:5	$3.58\times10^{-6}\pm8.05\times10^{-7}$	$2.98\times10^{-6}\pm4.44\times10^{-7}$	$4.26\times10^{-6}\pm1.23\times10^{-6}$	$4.56\times10^{-6}\pm9.07\times10^{-7}$

PI 36:4|PI 18:2_18:2	$3.89\times10^{-6}\pm1.08\times10^{-6}$	$1.81\times10^{-6}\pm1.00\times10^{-6}$	$3.70\times10^{-6}\pm1.07\times10^{-6}$	$4.12\times10^{-6}\pm1.88\times10^{-6}$
PI 36:4|PI 18:1_18:3	$3.19\times10^{-6}\pm1.58\times10^{-6}$	$5.92\times10^{-6}\pm2.97\times10^{-6}$	$3.67\times10^{-6}\pm1.02\times10^{-6}$	$4.11\times10^{-6}\pm1.94\times10^{-6}$
PI 36:4|PI 16:0_20:4	$1.93\times10^{-4}\pm3.68\times10^{-5}$	$9.99\times10^{-5}\pm2.38\times10^{-5}$	$1.84\times10^{-4}\pm6.21\times10^{-5}$	$2.19\times10^{-4}\pm1.39\times10^{-5}$
PI 36:4;O|PI 16:0_20:4;O	$1.19\times10^{-6}\pm3.77\times10^{-7}$	$1.70\times10^{-6}\pm1.43\times10^{-6}$	$1.60\times10^{-6}\pm7.19\times10^{-7}$	$9.09\times10^{-7}\pm1.52\times10^{-7}$
PI 36:4	$1.58\times10^{-4}\pm5.32\times10^{-5}$	$1.85\times10^{-4}\pm1.81\times10^{-5}$	$2.05\times10^{-4}\pm4.39\times10^{-5}$	$2.21\times10^{-4}\pm4.19\times10^{-5}$
PI 36:3|PI 18:1_18:2	$4.41\times10^{-5}\pm1.06\times10^{-5}$	$1.24\times10^{-4}\pm4.31\times10^{-5}$	$7.92\times10^{-5}\pm4.16\times10^{-5}$	$6.83\times10^{-5}\pm8.77\times10^{-6}$
PI 36:3|PI 16:0_20:3	$7.04\times10^{-5}\pm8.18\times10^{-6}$	$4.26\times10^{-5}\pm1.56\times10^{-5}$	$6.73\times10^{-5}\pm1.03\times10^{-5}$	$8.98\times10^{-5}\pm1.84\times10^{-5}$
PI 36:3;O|PI 18:1_18:2;O	$6.17\times10^{-7}\pm4.59\times10^{-7}$	$3.12\times10^{-6}\pm4.50\times10^{-6}$	$1.94\times10^{-6}\pm1.93\times10^{-6}$	$3.76\times10^{-7}\pm2.32\times10^{-7}$
PI 36:2|PI 18:1_18:1	$2.42\times10^{-4}\pm3.85\times10^{-5}$	$1.33\times10^{-4}\pm6.08\times10^{-5}$	$2.65\times10^{-4}\pm1.83\times10^{-5}$	$3.04\times10^{-4}\pm8.03\times10^{-5}$
PI 36:2|PI 18:0_18:2	$4.58\times10^{-4}\pm8.69\times10^{-5}$	$2.78\times10^{-4}\pm1.05\times10^{-4}$	$5.47\times10^{-4}\pm3.98\times10^{-5}$	$5.57\times10^{-4}\pm3.48\times10^{-4}$
PI 36:2	$4.36\times10^{-4}\pm5.43\times10^{-5}$	$4.45\times10^{-4}\pm1.43\times10^{-4}$	$7.83\times10^{-4}\pm1.64\times10^{-4}$	$6.16\times10^{-4}\pm1.93\times10^{-4}$
PI 36:1|PI 18:0_18:1	$5.52\times10^{-5}\pm2.53\times10^{-5}$	$8.36\times10^{-5}\pm2.82\times10^{-5}$	$1.12\times10^{-4}\pm4.40\times10^{-5}$	$6.23\times10^{-5}\pm4.64\times10^{-5}$
PI 36:1	$9.13\times10^{-5}\pm1.82\times10^{-5}$	$7.51\times10^{-5}\pm2.32\times10^{-5}$	$1.10\times10^{-4}\pm2.38\times10^{-5}$	$1.11\times10^{-4}\pm3.10\times10^{-5}$
PI 36:0|PI 18:0_18:0	$4.76\times10^{-5}\pm1.11\times10^{-5}$	$2.69\times10^{-5}\pm1.51\times10^{-5}$	$3.36\times10^{-5}\pm2.99\times10^{-5}$	$2.17\times10^{-5}\pm9.64\times10^{-6}$
PI 36:0	$5.91\times10^{-5}\pm2.12\times10^{-5}$	$2.11\times10^{-5}\pm6.47\times10^{-6}$	$2.89\times10^{-5}\pm2.20\times10^{-5}$	$1.92\times10^{-5}\pm4.52\times10^{-6}$
PI 35:2|PI 17:0_18:2	$6.40\times10^{-6}\pm3.00\times10^{-6}$	$8.05\times10^{-6}\pm2.95\times10^{-6}$	$1.17\times10^{-5}\pm2.26\times10^{-6}$	$1.01\times10^{-5}\pm3.70\times10^{-6}$
PI 34:3|PI 16:1_18:2	$1.69\times10^{-6}\pm3.60\times10^{-7}$	$1.72\times10^{-6}\pm4.74\times10^{-7}$	$2.03\times10^{-6}\pm6.31\times10^{-7}$	$2.68\times10^{-6}\pm1.05\times10^{-6}$
PI 34:3|PI 16:0_18:3	$4.18\times10^{-6}\pm1.37\times10^{-6}$	$6.32\times10^{-6}\pm3.11\times10^{-6}$	$4.80\times10^{-6}\pm1.19\times10^{-6}$	$8.61\times10^{-6}\pm4.43\times10^{-6}$
PI 34:2|PI 16:0_18:2	$1.34\times10^{-4}\pm3.75\times10^{-5}$	$8.41\times10^{-5}\pm2.32\times10^{-5}$	$1.43\times10^{-4}\pm1.33\times10^{-5}$	$4.43\times10^{-4}\pm8.69\times10^{-5}$
PI 34:2;O|PI 16:0_18:2;O	$1.05\times10^{-6}\pm5.06\times10^{-7}$	$1.92\times10^{-6}\pm1.87\times10^{-6}$	$1.86\times10^{-6}\pm1.58\times10^{-6}$	$4.05\times10^{-7}\pm1.52\times10^{-7}$
PI 34:1|PI 16:0_18:1	$2.62\times10^{-4}\pm3.48\times10^{-4}$	$6.96\times10^{-5}\pm2.15\times10^{-5}$	$8.26\times10^{-5}\pm2.11\times10^{-5}$	$7.65\times10^{-4}\pm8.12\times10^{-4}$
PI 34:1	$5.08\times10^{-5}\pm8.05\times10^{-6}$	$8.30\times10^{-5}\pm1.68\times10^{-5}$	$7.86\times10^{-5}\pm1.96\times10^{-5}$	$1.24\times10^{-4}\pm5.57\times10^{-5}$
PI 34:0|PI 16:0_18:0	$3.30\times10^{-6}\pm1.24\times10^{-6}$	$1.83\times10^{-7}\pm2.13\times10^{-7}$	$7.57\times10^{-7}\pm6.53\times10^{-7}$	$1.73\times10^{-6}\pm7.28\times10^{-7}$
PI 32:1|PI 16:0_16:1	$9.50\times10^{-6}\pm3.87\times10^{-6}$	$7.45\times10^{-6}\pm3.03\times10^{-6}$	$1.39\times10^{-5}\pm5.80\times10^{-6}$	$1.16\times10^{-5}\pm5.13\times10^{-6}$
PG O-36:3|PG O-18:2_18:1	$1.43\times10^{-6}\pm4.39\times10^{-7}$	$2.09\times10^{-7}\pm5.89\times10^{-8}$	$2.85\times10^{-7}\pm1.14\times10^{-7}$	$8.17\times10^{-7}\pm1.46\times10^{-7}$
PG O-34:2|PG O-16:1_18:1	$5.43\times10^{-6}\pm7.99\times10^{-7}$	$1.01\times10^{-6}\pm3.40\times10^{-7}$	$2.21\times10^{-6}\pm6.42\times10^{-7}$	$4.35\times10^{-6}\pm1.37\times10^{-6}$

PG O-34:1|PG O-16:1_18:0	$1.14\times10^{-4}\pm4.31\times10^{-5}$	$4.81\times10^{-5}\pm9.23\times10^{-6}$	$7.97\times10^{-5}\pm5.20\times10^{-5}$	$5.28\times10^{-5}\pm1.20\times10^{-5}$
PG 44:12|PG 22:6_22:6	$1.24\times10^{-5}\pm4.31\times10^{-6}$	$9.45\times10^{-6}\pm3.33\times10^{-6}$	$6.05\times10^{-6}\pm1.79\times10^{-6}$	$1.35\times10^{-5}\pm4.25\times10^{-6}$
PG 44:11|PG 22:5_22:6	$1.68\times10^{-5}\pm3.39\times10^{-6}$	$8.53\times10^{-6}\pm2.48\times10^{-6}$	$1.30\times10^{-5}\pm6.26\times10^{-6}$	$1.04\times10^{-5}\pm3.60\times10^{-6}$
PG 44:10|PG 22:5_22:5	$2.77\times10^{-6}\pm1.29\times10^{-6}$	$2.79\times10^{-6}\pm7.98\times10^{-7}$	$2.33\times10^{-6}\pm8.82\times10^{-7}$	$2.39\times10^{-6}\pm8.83\times10^{-7}$
PG 42:8|PG 20:3_22:5	$4.78\times10^{-6}\pm7.04\times10^{-7}$	$7.30\times10^{-6}\pm5.00\times10^{-6}$	$3.38\times10^{-6}\pm1.43\times10^{-6}$	$3.13\times10^{-6}\pm7.49\times10^{-7}$
PG 42:11|PG 20:5_22:6	$1.32\times10^{-7}\pm1.21\times10^{-7}$	$4.62\times10^{-7}\pm5.17\times10^{-7}$	$1.10\times10^{-7}\pm6.20\times10^{-8}$	$2.33\times10^{-7}\pm1.21\times10^{-7}$
PG 42:10|PG 20:4_22:6	$1.19\times10^{-5}\pm2.81\times10^{-6}$	$5.60\times10^{-6}\pm2.90\times10^{-6}$	$4.94\times10^{-6}\pm2.39\times10^{-6}$	$6.54\times10^{-6}\pm2.47\times10^{-6}$
PG 40:8|PG 18:2_22:6	$9.95\times10^{-6}\pm3.08\times10^{-6}$	$6.79\times10^{-6}\pm3.11\times10^{-6}$	$9.55\times10^{-6}\pm2.96\times10^{-6}$	$5.88\times10^{-6}\pm2.11\times10^{-6}$
PG 40:7|PG 18:1_22:6	$2.49\times10^{-5}\pm3.81\times10^{-6}$	$2.16\times10^{-5}\pm7.40\times10^{-6}$	$2.37\times10^{-5}\pm5.54\times10^{-6}$	$1.75\times10^{-5}\pm6.16\times10^{-6}$
PG 40:6|PG 18:2_22:4	$5.75\times10^{-6}\pm8.28\times10^{-7}$	$7.02\times10^{-6}\pm3.43\times10^{-6}$	$5.11\times10^{-6}\pm1.31\times10^{-6}$	$4.66\times10^{-6}\pm1.35\times10^{-6}$
PG 40:6|PG 18:1_22:5	$1.26\times10^{-5}\pm2.07\times10^{-6}$	$1.41\times10^{-5}\pm4.05\times10^{-6}$	$1.72\times10^{-5}\pm6.97\times10^{-6}$	$1.32\times10^{-5}\pm2.74\times10^{-6}$
PG 40:5|PG 18:1_22:4	$9.26\times10^{-6}\pm1.63\times10^{-6}$	$8.45\times10^{-6}\pm3.52\times10^{-6}$	$1.02\times10^{-5}\pm7.70\times10^{-6}$	$8.69\times10^{-6}\pm1.87\times10^{-6}$
PG 38:7|PG 16:1_22:6	$1.27\times10^{-6}\pm3.87\times10^{-7}$	$1.09\times10^{-6}\pm7.34\times10^{-7}$	$1.02\times10^{-6}\pm3.04\times10^{-7}$	$1.06\times10^{-6}\pm4.08\times10^{-7}$
PG 38:6|PG 18:2_20:4	$1.78\times10^{-5}\pm4.82\times10^{-6}$	$8.72\times10^{-6}\pm3.56\times10^{-6}$	$1.18\times10^{-5}\pm4.49\times10^{-6}$	$1.15\times10^{-5}\pm3.88\times10^{-6}$
PG 38:6|PG 16:0_22:6	$8.65\times10^{-6}\pm2.95\times10^{-6}$	$4.92\times10^{-6}\pm3.42\times10^{-6}$	$2.17\times10^{-6}\pm7.59\times10^{-7}$	$1.07\times10^{-5}\pm6.14\times10^{-6}$
PG 38:3|PG 20:1_18:2	$2.95\times10^{-6}\pm7.32\times10^{-7}$	$9.36\times10^{-7}\pm2.43\times10^{-7}$	$2.00\times10^{-6}\pm9.13\times10^{-7}$	$2.47\times10^{-6}\pm6.94\times10^{-7}$
PG 38:3|PG 18:1_20:2	$2.25\times10^{-6}\pm4.63\times10^{-7}$	$1.62\times10^{-6}\pm3.79\times10^{-7}$	$2.19\times10^{-6}\pm7.63\times10^{-7}$	$1.96\times10^{-6}\pm2.97\times10^{-7}$
PG 38:2|PG 18:0_20:2	$1.20\times10^{-6}\pm5.36\times10^{-7}$	$8.04\times10^{-8}\pm5.41\times10^{-8}$	$4.43\times10^{-7}\pm2.93\times10^{-7}$	$1.63\times10^{-6}\pm9.66\times10^{-7}$
PG 36:4|PG 18:2_18:2	$2.68\times10^{-4}\pm9.51\times10^{-5}$	$2.32\times10^{-4}\pm4.83\times10^{-5}$	$2.40\times10^{-4}\pm9.46\times10^{-5}$	$4.03\times10^{-4}\pm1.04\times10^{-4}$
PG 36:4|PG 16:0_20:4	$4.75\times10^{-6}\pm2.31\times10^{-6}$	$2.49\times10^{-6}\pm7.28\times10^{-7}$	$1.89\times10^{-6}\pm4.16\times10^{-7}$	$8.99\times10^{-6}\pm5.29\times10^{-6}$
PG 36:3|PG 18:1_18:2	$3.15\times10^{-5}\pm8.62\times10^{-6}$	$6.29\times10^{-5}\pm2.00\times10^{-5}$	$4.16\times10^{-5}\pm1.26\times10^{-5}$	$3.89\times10^{-5}\pm1.10\times10^{-5}$
PG 36:2|PG 18:1_18:1	$3.54\times10^{-5}\pm5.34\times10^{-6}$	$2.93\times10^{-5}\pm1.25\times10^{-5}$	$3.23\times10^{-5}\pm1.68\times10^{-5}$	$3.09\times10^{-5}\pm1.11\times10^{-5}$
PG 36:2|PG 18:0_18:2	$1.33\times10^{-4}\pm1.56\times10^{-5}$	$8.01\times10^{-5}\pm1.44\times10^{-5}$	$1.27\times10^{-4}\pm3.98\times10^{-5}$	$1.36\times10^{-4}\pm6.63\times10^{-5}$
PG 36:1|PG 18:0_18:1	$1.19\times10^{-5}\pm1.47\times10^{-6}$	$2.09\times10^{-5}\pm3.30\times10^{-6}$	$2.47\times10^{-5}\pm5.49\times10^{-6}$	$6.55\times10^{-6}\pm1.22\times10^{-6}$
PG 35:2|PG 17:0_18:2	$2.66\times10^{-7}\pm2.28\times10^{-7}$	$3.74\times10^{-7}\pm2.65\times10^{-7}$	$3.28\times10^{-8}\pm6.55\times10^{-8}$	$1.20\times10^{-6}\pm4.48\times10^{-7}$
PG 35:1|PG 17:0_18:1	-	$9.20\times10^{-7}\pm3.30\times10^{-7}$	$1.78\times10^{-7}\pm1.20\times10^{-7}$	$3.54\times10^{-7}\pm3.01\times10^{-7}$

PG 35:1|PG 16:0_19:1	$8.50\times10^{-6}\pm2.01\times10^{-6}$	$1.54\times10^{-5}\pm2.71\times10^{-6}$	$1.08\times10^{-5}\pm1.24\times10^{-6}$	$1.33\times10^{-5}\pm5.30\times10^{-6}$
PG 34:2|PG 16:0_18:2	$4.73\times10^{-6}\pm1.99\times10^{-6}$	$7.94\times10^{-7}\pm5.69\times10^{-7}$	$5.66\times10^{-7}\pm2.03\times10^{-7}$	$1.51\times10^{-5}\pm2.92\times10^{-6}$
PG 34:1|PG 16:0_18:1	$1.58\times10^{-4}\pm1.37\times10^{-5}$	$6.39\times10^{-5}\pm1.19\times10^{-5}$	$1.17\times10^{-4}\pm1.86\times10^{-5}$	$2.41\times10^{-4}\pm2.55\times10^{-5}$
PG 32:1|PG 16:0_16:1	$1.55\times10^{-7}\pm7.24\times10^{-8}$	$1.46\times10^{-7}\pm1.53\times10^{-7}$	$6.18\times10^{-8}\pm4.45\times10^{-8}$	$4.39\times10^{-8}\pm1.63\times10^{-8}$
PG 32:0|PG 16:0_16:0	$7.93\times10^{-7}\pm2.84\times10^{-7}$	$4.24\times10^{-7}\pm1.68\times10^{-7}$	$2.87\times10^{-7}\pm3.30\times10^{-7}$	$3.70\times10^{-7}\pm1.13\times10^{-7}$
PE O-42:7|PE O-20:1_22:6	$8.59\times10^{-6}\pm1.38\times10^{-6}$	$9.03\times10^{-6}\pm3.54\times10^{-6}$	$7.20\times10^{-6}\pm2.65\times10^{-6}$	$7.27\times10^{-6}\pm1.49\times10^{-6}$
PE O-42:6|PE O-20:0_22:6	$9.81\times10^{-6}\pm3.50\times10^{-6}$	$6.85\times10^{-6}\pm1.80\times10^{-6}$	$1.10\times10^{-5}\pm4.03\times10^{-6}$	$1.62\times10^{-5}\pm1.84\times10^{-6}$
PE O-40:6|PE O-18:0_22:6	$2.25\times10^{-5}\pm3.14\times10^{-6}$	$2.35\times10^{-5}\pm6.22\times10^{-6}$	$2.87\times10^{-5}\pm5.78\times10^{-6}$	$1.49\times10^{-5}\pm4.04\times10^{-6}$
PE O-40:5|PE O-20:1_20:4	$3.82\times10^{-5}\pm8.45\times10^{-6}$	$3.39\times10^{-5}\pm9.55\times10^{-6}$	$3.40\times10^{-5}\pm1.10\times10^{-5}$	$2.85\times10^{-5}\pm5.41\times10^{-6}$
PE O-40:4|PE O-18:0_22:4	$9.64\times10^{-6}\pm1.82\times10^{-6}$	$1.21\times10^{-5}\pm2.25\times10^{-6}$	$1.24\times10^{-5}\pm4.99\times10^{-6}$	$1.18\times10^{-5}\pm1.94\times10^{-6}$
PE O-38:7|PE O-16:1_22:6	$2.24\times10^{-4}\pm4.56\times10^{-5}$	$1.08\times10^{-4}\pm3.93\times10^{-5}$	$1.76\times10^{-4}\pm6.01\times10^{-5}$	$7.85\times10^{-5}\pm6.10\times10^{-5}$
PE O-38:6|PE O-16:1_22:5	$1.46\times10^{-4}\pm3.05\times10^{-5}$	$1.30\times10^{-4}\pm1.74\times10^{-5}$	$1.71\times10^{-4}\pm1.41\times10^{-5}$	$9.37\times10^{-5}\pm9.15\times10^{-6}$
PE O-38:2|PE O-18:1_20:1	$3.66\times10^{-6}\pm5.13\times10^{-7}$	$7.70\times10^{-6}\pm4.08\times10^{-6}$	$4.24\times10^{-6}\pm9.85\times10^{-7}$	$4.21\times10^{-6}\pm3.74\times10^{-7}$
PE O-36:5|PE O-16:1_20:4	$1.11\times10^{-4}\pm7.10\times10^{-5}$	$2.29\times10^{-4}\pm5.02\times10^{-5}$	$3.31\times10^{-4}\pm1.80\times10^{-4}$	$8.04\times10^{-5}\pm6.81\times10^{-5}$
PE O-36:4|PE O-16:1_20:3	$1.91\times10^{-6}\pm6.29\times10^{-7}$	$3.74\times10^{-7}\pm2.97\times10^{-7}$	$9.67\times10^{-7}\pm4.26\times10^{-7}$	$2.46\times10^{-6}\pm7.06\times10^{-7}$
PE O-36:3|PE O-18:2_18:1	$3.94\times10^{-6}\pm1.18\times10^{-6}$	$1.71\times10^{-5}\pm7.67\times10^{-6}$	$7.08\times10^{-6}\pm1.51\times10^{-6}$	$7.40\times10^{-6}\pm1.38\times10^{-6}$
PE O-34:3|PE O-16:1_18:2	$2.23\times10^{-7}\pm6.88\times10^{-8}$	$8.17\times10^{-8}\pm5.47\times10^{-8}$	$1.18\times10^{-7}\pm7.81\times10^{-8}$	$2.42\times10^{-7}\pm5.10\times10^{-8}$
PE O-20:5	$2.10\times10^{-5}\pm3.87\times10^{-6}$	$3.07\times10^{-5}\pm1.16\times10^{-5}$	$3.92\times10^{-5}\pm1.23\times10^{-5}$	$3.45\times10^{-5}\pm6.57\times10^{-6}$
PE O-20:4	$1.48\times10^{-3}\pm2.72\times10^{-4}$	$1.56\times10^{-3}\pm4.50\times10^{-4}$	$1.96\times10^{-3}\pm6.54\times10^{-4}$	$1.60\times10^{-3}\pm2.81\times10^{-4}$
PE O-18:0	$3.79\times10^{-5}\pm8.80\times10^{-6}$	$2.61\times10^{-5}\pm5.00\times10^{-6}$	$2.94\times10^{-5}\pm7.59\times10^{-6}$	$3.28\times10^{-5}\pm9.79\times10^{-6}$
PE 44:12|PE 22:6_22:6	$1.17\times10^{-4}\pm4.20\times10^{-5}$	$8.39\times10^{-5}\pm3.71\times10^{-5}$	$7.94\times10^{-5}\pm3.16\times10^{-5}$	$7.55\times10^{-5}\pm3.30\times10^{-5}$
PE 42:9	$9.17\times10^{-5}\pm9.24\times10^{-6}$	$8.93\times10^{-5}\pm5.25\times10^{-5}$	$8.01\times10^{-5}\pm2.48\times10^{-5}$	$5.77\times10^{-5}\pm1.55\times10^{-5}$
PE 42:8|PE 20:3_22:5	$3.53\times10^{-6}\pm7.12\times10^{-7}$	$1.84\times10^{-6}\pm6.81\times10^{-7}$	$2.76\times10^{-6}\pm1.40\times10^{-6}$	$3.92\times10^{-6}\pm9.55\times10^{-7}$
PE 42:8|PE 20:2_22:6	$6.12\times10^{-6}\pm2.74\times10^{-7}$	$7.13\times10^{-6}\pm2.41\times10^{-6}$	$8.81\times10^{-6}\pm1.28\times10^{-6}$	$8.16\times10^{-6}\pm8.29\times10^{-7}$
PE 42:8	$5.76\times10^{-5}\pm2.09\times10^{-5}$	$4.91\times10^{-5}\pm1.85\times10^{-5}$	$5.50\times10^{-5}\pm1.55\times10^{-5}$	$5.37\times10^{-5}\pm1.58\times10^{-5}$
PE 42:6|PE 20:0_22:6	$6.30\times10^{-6}\pm3.54\times10^{-6}$	$1.10\times10^{-6}\pm9.93\times10^{-7}$	$4.38\times10^{-6}\pm2.06\times10^{-6}$	$8.63\times10^{-6}\pm3.79\times10^{-6}$

PE 42:6	$5.36\times10^{-6}\pm4.52\times10^{-6}$	$6.53\times10^{-6}\pm3.33\times10^{-6}$	$9.62\times10^{-6}\pm6.63\times10^{-6}$	$1.27\times10^{-5}\pm5.29\times10^{-6}$
PE 40:8|PE 18:2_22:6	$2.12\times10^{-4}\pm3.99\times10^{-5}$	$1.20\times10^{-4}\pm2.65\times10^{-5}$	$1.39\times10^{-4}\pm5.19\times10^{-5}$	$1.10\times10^{-4}\pm2.26\times10^{-5}$
PE 40:7|PE 18:1_22:6	$3.81\times10^{-4}\pm1.72\times10^{-4}$	$5.67\times10^{-4}\pm1.75\times10^{-4}$	$5.02\times10^{-4}\pm1.64\times10^{-4}$	$5.89\times10^{-4}\pm1.11\times10^{-4}$
PE 40:6|PE 18:1_22:5	$3.25\times10^{-6}\pm6.54\times10^{-7}$	$2.51\times10^{-6}\pm6.11\times10^{-7}$	$2.89\times10^{-6}\pm9.24\times10^{-7}$	$3.72\times10^{-6}\pm7.91\times10^{-7}$
PE 40:6|PE 18:0_22:6	$2.41\times10^{-3}\pm4.92\times10^{-4}$	$2.12\times10^{-3}\pm6.67\times10^{-4}$	$2.96\times10^{-3}\pm6.49\times10^{-4}$	$3.64\times10^{-2}\pm6.38\times10^{-2}$
PE 40:5|PE 18:0_22:5	$5.36\times10^{-5}\pm7.47\times10^{-6}$	$1.03\times10^{-5}\pm4.67\times10^{-6}$	$3.92\times10^{-5}\pm1.52\times10^{-5}$	$3.87\times10^{-5}\pm8.98\times10^{-6}$
PE 39:6|PE 17:0_22:6	$3.52\times10^{-5}\pm6.57\times10^{-6}$	$1.61\times10^{-5}\pm7.74\times10^{-6}$	$3.17\times10^{-5}\pm6.59\times10^{-6}$	$5.96\times10^{-5}\pm2.56\times10^{-5}$
PE 39:4|PE 19:0_20:4	$6.96\times10^{-6}\pm1.15\times10^{-6}$	$5.04\times10^{-6}\pm7.62\times10^{-7}$	$6.46\times10^{-6}\pm1.41\times10^{-6}$	$1.70\times10^{-5}\pm4.04\times10^{-6}$
PE 38:7|PE 18:3_20:4	$3.10\times10^{-6}\pm6.15\times10^{-7}$	$2.87\times10^{-6}\pm8.62\times10^{-7}$	$4.50\times10^{-6}\pm1.50\times10^{-6}$	$4.03\times10^{-6}\pm1.28\times10^{-6}$
PE 38:7|PE 18:2_20:5	$1.15\times10^{-5}\pm9.47\times10^{-6}$	$4.58\times10^{-6}\pm1.14\times10^{-6}$	$4.24\times10^{-6}\pm7.28\times10^{-7}$	$5.02\times10^{-5}\pm7.79\times10^{-5}$
PE 38:7|PE 16:1_22:6	$3.88\times10^{-5}\pm8.89\times10^{-6}$	$3.72\times10^{-5}\pm2.97\times10^{-6}$	$4.94\times10^{-5}\pm5.28\times10^{-6}$	$4.32\times10^{-5}\pm1.66\times10^{-5}$
PE 38:6|PE 18:2_20:4	$1.32\times10^{-4}\pm1.87\times10^{-5}$	$7.26\times10^{-5}\pm3.40\times10^{-5}$	$1.20\times10^{-4}\pm4.94\times10^{-5}$	$9.74\times10^{-5}\pm9.13\times10^{-6}$
PE 38:6|PE 16:0_22:6	$1.75\times10^{-4}\pm3.75\times10^{-5}$	$1.25\times10^{-4}\pm3.41\times10^{-5}$	$1.10\times10^{-4}\pm3.82\times10^{-5}$	$2.43\times10^{-4}\pm3.92\times10^{-5}$
PE 38:6;O|PE 16:0_22:6;O	$3.84\times10^{-6}\pm1.02\times10^{-6}$	$7.64\times10^{-6}\pm4.90\times10^{-6}$	$7.55\times10^{-6}\pm3.96\times10^{-6}$	$7.10\times10^{-6}\pm1.23\times10^{-6}$
PE 38:6	$3.07\times10^{-4}\pm1.76\times10^{-4}$	$1.53\times10^{-4}\pm6.42\times10^{-5}$	$3.39\times10^{-4}\pm1.34\times10^{-4}$	$2.62\times10^{-4}\pm9.51\times10^{-5}$
PE 38:5|PE 18:1_20:4	$7.27\times10^{-4}\pm1.45\times10^{-4}$	$3.39\times10^{-4}\pm7.16\times10^{-5}$	$5.16\times10^{-4}\pm1.20\times10^{-4}$	$1.23\times10^{-3}\pm3.82\times10^{-4}$
PE 38:5|PE 16:0_22:5	$1.33\times10^{-4}\pm2.40\times10^{-5}$	$1.05\times10^{-4}\pm3.03\times10^{-5}$	$1.21\times10^{-4}\pm3.22\times10^{-5}$	$1.92\times10^{-4}\pm5.08\times10^{-5}$
PE 38:5;O|PE 18:1_20:4;O	$1.62\times10^{-6}\pm6.48\times10^{-7}$	$2.34\times10^{-6}\pm1.06\times10^{-6}$	$3.22\times10^{-6}\pm1.53\times10^{-6}$	$1.80\times10^{-6}\pm3.29\times10^{-7}$
PE 38:5;2O|PE 18:1_20:4;2O	$1.43\times10^{-6}\pm6.17\times10^{-7}$	$3.70\times10^{-6}\pm3.89\times10^{-6}$	$2.49\times10^{-6}\pm1.52\times10^{-6}$	$7.92\times10^{-7}\pm2.32\times10^{-7}$
PE 38:4|PE 18:1_20:3	$3.22\times10^{-5}\pm4.39\times10^{-6}$	$6.74\times10^{-5}\pm3.06\times10^{-5}$	$6.25\times10^{-5}\pm1.69\times10^{-5}$	$5.61\times10^{-5}\pm7.40\times10^{-6}$
PE 38:4|PE 18:0_20:4	$2.50\times10^{-3}\pm5.62\times10^{-4}$	$2.57\times10^{-3}\pm4.95\times10^{-4}$	$2.84\times10^{-3}\pm6.47\times10^{-4}$	$3.06\times10^{-3}\pm2.03\times10^{-3}$
PE 38:4	$1.35\times10^{-4}\pm3.18\times10^{-5}$	$8.53\times10^{-5}\pm3.13\times10^{-5}$	$1.26\times10^{-4}\pm3.02\times10^{-5}$	$1.15\times10^{-4}\pm2.81\times10^{-5}$
PE 38:3|PE 18:0_20:3	$1.12\times10^{-3}\pm2.56\times10^{-4}$	$1.35\times10^{-3}\pm6.26\times10^{-4}$	$1.27\times10^{-3}\pm6.06\times10^{-5}$	$1.80\times10^{-3}\pm2.59\times10^{-4}$
PE 38:2|PE 18:1_20:1	$6.01\times10^{-5}\pm3.54\times10^{-6}$	$5.69\times10^{-5}\pm1.38\times10^{-5}$	$6.73\times10^{-5}\pm6.13\times10^{-6}$	$7.64\times10^{-5}\pm7.54\times10^{-6}$
PE 38:1|PE 18:0_20:1	$2.82\times10^{-5}\pm8.25\times10^{-6}$	$3.76\times10^{-5}\pm1.11\times10^{-5}$	$4.29\times10^{-5}\pm1.59\times10^{-5}$	$4.60\times10^{-5}\pm6.35\times10^{-6}$
PE 37:3|PE 17:0_20:3	$2.31\times10^{-6}\pm3.25\times10^{-7}$	$1.55\times10^{-6}\pm8.74\times10^{-7}$	$2.75\times10^{-6}\pm7.75\times10^{-7}$	$2.33\times10^{-6}\pm8.46\times10^{-7}$

PE 37:2|PE 19:0_18:2	$1.84\times10^{-6}\pm4.69\times10^{-7}$	$4.65\times10^{-6}\pm9.92\times10^{-7}$	$4.70\times10^{-6}\pm8.83\times10^{-7}$	$5.74\times10^{-6}\pm1.93\times10^{-6}$
PE 36:6|PE 14:0_22:6	$3.30\times10^{-6}\pm1.09\times10^{-6}$	$3.97\times10^{-6}\pm1.67\times10^{-6}$	$4.74\times10^{-6}\pm1.57\times10^{-6}$	$5.07\times10^{-6}\pm2.02\times10^{-6}$
PE 36:5|PE 18:2_18:3	$1.04\times10^{-6}\pm1.63\times10^{-6}$	$1.72\times10^{-9}\pm3.44\times10^{-9}$	$1.17\times10^{-6}\pm1.60\times10^{-6}$	$7.63\times10^{-7}\pm6.11\times10^{-7}$
PE 36:5|PE 16:1_20:4	$7.59\times10^{-6}\pm1.24\times10^{-6}$	$5.79\times10^{-6}\pm7.30\times10^{-7}$	$6.91\times10^{-6}\pm1.33\times10^{-6}$	$1.04\times10^{-5}\pm9.01\times10^{-7}$
PE 36:5|PE 16:0_20:5	$6.43\times10^{-6}\pm9.21\times10^{-7}$	$6.05\times10^{-6}\pm2.38\times10^{-6}$	$6.59\times10^{-6}\pm4.01\times10^{-6}$	$1.62\times10^{-5}\pm3.78\times10^{-6}$
PE 36:4|PE 18:2_18:2	$3.07\times10^{-4}\pm6.88\times10^{-5}$	$2.12\times10^{-4}\pm6.34\times10^{-5}$	$3.34\times10^{-4}\pm4.78\times10^{-5}$	$3.28\times10^{-4}\pm7.37\times10^{-5}$
PE 36:4|PE 16:0_20:4	$6.38\times10^{-4}\pm1.35\times10^{-4}$	$3.68\times10^{-4}\pm8.20\times10^{-5}$	$5.51\times10^{-4}\pm8.52\times10^{-5}$	$9.72\times10^{-4}\pm2.16\times10^{-4}$
PE 36:3|PE 18:1_18:2	$1.40\times10^{-3}\pm1.82\times10^{-4}$	$1.81\times10^{-3}\pm5.48\times10^{-4}$	$2.42\times10^{-3}\pm6.47\times10^{-4}$	$2.30\times10^{-3}\pm6.16\times10^{-4}$
PE 36:3|PE 16:0_20:3	$8.10\times10^{-5}\pm1.36\times10^{-5}$	$3.92\times10^{-5}\pm8.05\times10^{-6}$	$6.51\times10^{-5}\pm2.06\times10^{-5}$	$1.16\times10^{-4}\pm1.11\times10^{-5}$
PE 36:2|PE 18:1_18:1	$9.82\times10^{-5}\pm4.04\times10^{-5}$	$1.25\times10^{-4}\pm3.13\times10^{-5}$	$7.88\times10^{-5}\pm1.39\times10^{-5}$	$1.21\times10^{-4}\pm5.73\times10^{-5}$
PE 36:2|PE 18:0_18:2	$4.05\times10^{-3}\pm1.39\times10^{-3}$	$3.50\times10^{-3}\pm8.37\times10^{-4}$	$4.37\times10^{-3}\pm7.75\times10^{-4}$	$6.14\times10^{-3}\pm1.16\times10^{-3}$
PE 36:1|PE 18:0_18:1	$1.68\times10^{-3}\pm3.81\times10^{-4}$	$2.20\times10^{-3}\pm6.92\times10^{-4}$	$2.44\times10^{-3}\pm5.66\times10^{-4}$	$2.49\times10^{-3}\pm5.92\times10^{-4}$
PE 36:0|PE 18:0_18:0	$5.72\times10^{-5}\pm1.44\times10^{-5}$	$2.14\times10^{-5}\pm1.39\times10^{-5}$	$3.23\times10^{-5}\pm1.22\times10^{-5}$	$1.96\times10^{-5}\pm6.16\times10^{-6}$
PE 35:1|PE 17:0_18:1	$1.22\times10^{-5}\pm1.40\times10^{-6}$	$1.21\times10^{-5}\pm3.18\times10^{-6}$	$1.99\times10^{-5}\pm2.74\times10^{-6}$	$3.77\times10^{-5}\pm1.07\times10^{-5}$
PE 34:3|PE 16:1_18:2	$4.72\times10^{-5}\pm1.05\times10^{-5}$	$3.97\times10^{-5}\pm7.11\times10^{-6}$	$6.27\times10^{-5}\pm1.20\times10^{-5}$	$6.75\times10^{-5}\pm2.22\times10^{-5}$
PE 34:3|PE 16:0_18:3	$6.60\times10^{-6}\pm3.33\times10^{-6}$	$1.43\times10^{-5}\pm2.86\times10^{-6}$	$2.09\times10^{-5}\pm8.07\times10^{-6}$	$3.67\times10^{-5}\pm1.24\times10^{-5}$
PE 34:2|PE 16:0_18:2	$3.06\times10^{-4}\pm4.79\times10^{-5}$	$1.86\times10^{-4}\pm4.08\times10^{-5}$	$3.07\times10^{-4}\pm5.53\times10^{-5}$	$6.11\times10^{-4}\pm7.73\times10^{-5}$
PE 34:2;O|PE 16:0_18:2;O	$4.68\times10^{-7}\pm2.53\times10^{-7}$	$9.49\times10^{-7}\pm6.07\times10^{-7}$	$1.08\times10^{-6}\pm7.66\times10^{-7}$	$7.05\times10^{-7}\pm3.99\times10^{-7}$
PE 34:2;2O|PE 16:0_18:2;2O	$4.65\times10^{-7}\pm1.11\times10^{-7}$	$8.43\times10^{-7}\pm7.78\times10^{-7}$	$9.15\times10^{-7}\pm7.61\times10^{-7}$	$3.50\times10^{-7}\pm1.00\times10^{-7}$
PE 34:1|PE 16:0_18:1	$1.24\times10^{-3}\pm1.22\times10^{-4}$	$1.10\times10^{-3}\pm3.07\times10^{-4}$	$1.92\times10^{-3}\pm2.37\times10^{-4}$	$2.83\times10^{-3}\pm8.03\times10^{-4}$
PE 34:0|PE 16:0_18:0	$4.87\times10^{-4}\pm1.19\times10^{-4}$	$2.67\times10^{-4}\pm8.73\times10^{-5}$	$3.86\times10^{-4}\pm3.06\times10^{-4}$	$2.25\times10^{-4}\pm9.45\times10^{-5}$
PE 32:1|PE 16:0_16:1	$6.13\times10^{-6}\pm1.63\times10^{-6}$	$3.55\times10^{-6}\pm7.96\times10^{-7}$	$7.82\times10^{-6}\pm1.17\times10^{-6}$	$1.19\times10^{-5}\pm2.61\times10^{-6}$
PE 32:0|PE 16:0_16:0	$2.38\times10^{-5}\pm2.38\times10^{-5}$	$1.55\times10^{-5}\pm1.15\times10^{-5}$	$1.94\times10^{-5}\pm2.93\times10^{-5}$	$3.05\times10^{-6}\pm5.23\times10^{-6}$
PC O-47:6	$6.30\times10^{-7}\pm7.40\times10^{-7}$	$9.63\times10^{-7}\pm1.03\times10^{-6}$	$8.16\times10^{-7}\pm1.25\times10^{-6}$	$1.76\times10^{-6}\pm2.60\times10^{-6}$
PC O-44:7	$1.97\times10^{-5}\pm2.55\times10^{-6}$	$2.53\times10^{-5}\pm9.94\times10^{-6}$	$1.63\times10^{-5}\pm7.26\times10^{-6}$	$2.25\times10^{-5}\pm6.11\times10^{-6}$
PC O-44:6	$1.61\times10^{-5}\pm4.61\times10^{-6}$	$2.31\times10^{-5}\pm6.07\times10^{-6}$	$2.39\times10^{-5}\pm7.19\times10^{-6}$	$1.63\times10^{-5}\pm5.93\times10^{-6}$

PC O-44:5	$1.20\times10^{-5}\pm1.04\times10^{-6}$	$1.07\times10^{-5}\pm2.87\times10^{-6}$	$1.12\times10^{-5}\pm2.14\times10^{-6}$	$1.14\times10^{-5}\pm2.52\times10^{-6}$
PC O-44:12	$3.29\times10^{-5}\pm6.33\times10^{-6}$	$2.23\times10^{-5}\pm6.50\times10^{-6}$	$2.29\times10^{-5}\pm8.19\times10^{-6}$	$1.12\times10^{-5}\pm3.14\times10^{-6}$
PC O-42:9	$3.74\times10^{-5}\pm1.63\times10^{-5}$	$1.94\times10^{-5}\pm6.71\times10^{-6}$	$2.05\times10^{-5}\pm5.15\times10^{-6}$	$4.28\times10^{-5}\pm1.83\times10^{-5}$
PC O-42:7	$5.07\times10^{-5}\pm8.51\times10^{-6}$	$4.97\times10^{-5}\pm2.31\times10^{-5}$	$4.27\times10^{-5}\pm1.74\times10^{-5}$	$5.26\times10^{-5}\pm1.98\times10^{-5}$
PC O-42:6	$2.14\times10^{-4}\pm2.07\times10^{-5}$	$1.83\times10^{-4}\pm4.42\times10^{-5}$	$1.98\times10^{-4}\pm9.37\times10^{-5}$	$2.38\times10^{-4}\pm6.90\times10^{-5}$
PC O-42:5	$4.84\times10^{-5}\pm7.49\times10^{-6}$	$5.13\times10^{-5}\pm1.77\times10^{-5}$	$4.35\times10^{-5}\pm1.49\times10^{-5}$	$5.89\times10^{-5}\pm1.38\times10^{-5}$
PC O-42:4	$1.64\times10^{-5}\pm3.48\times10^{-6}$	$1.83\times10^{-5}\pm3.08\times10^{-6}$	$2.01\times10^{-5}\pm4.58\times10^{-6}$	$1.29\times10^{-5}\pm1.68\times10^{-6}$
PC O-40:7	$5.08\times10^{-4}\pm1.42\times10^{-4}$	$3.54\times10^{-4}\pm1.71\times10^{-4}$	$5.00\times10^{-4}\pm1.62\times10^{-4}$	$3.69\times10^{-4}\pm9.13\times10^{-5}$
PC O-40:6	$3.93\times10^{-4}\pm8.06\times10^{-5}$	$3.53\times10^{-4}\pm1.20\times10^{-4}$	$2.99\times10^{-4}\pm6.23\times10^{-5}$	$2.97\times10^{-4}\pm6.80\times10^{-5}$
PC O-40:5	$3.67\times10^{-5}\pm7.98\times10^{-6}$	$2.51\times10^{-5}\pm4.48\times10^{-6}$	$3.59\times10^{-5}\pm1.16\times10^{-5}$	$4.29\times10^{-5}\pm8.20\times10^{-6}$
PC O-40:4	$1.43\times10^{-4}\pm3.96\times10^{-5}$	$1.43\times10^{-4}\pm5.48\times10^{-5}$	$1.61\times10^{-4}\pm4.03\times10^{-5}$	$1.41\times10^{-4}\pm3.27\times10^{-5}$
PC O-40:3	$2.27\times10^{-5}\pm4.39\times10^{-6}$	$3.82\times10^{-5}\pm1.29\times10^{-5}$	$2.45\times10^{-5}\pm5.52\times10^{-6}$	$2.37\times10^{-5}\pm5.42\times10^{-6}$
PC O-39:7	$4.28\times10^{-5}\pm2.04\times10^{-5}$	$4.80\times10^{-5}\pm1.92\times10^{-5}$	$3.87\times10^{-5}\pm1.94\times10^{-5}$	$6.55\times10^{-5}\pm2.63\times10^{-5}$
PC O-39:6	$4.40\times10^{-5}\pm8.20\times10^{-6}$	$5.33\times10^{-5}\pm2.53\times10^{-5}$	$6.35\times10^{-5}\pm1.69\times10^{-5}$	$5.21\times10^{-5}\pm8.75\times10^{-6}$
PC O-38:7	$1.01\times10^{-4}\pm3.39\times10^{-5}$	$7.53\times10^{-5}\pm2.94\times10^{-5}$	$7.08\times10^{-5}\pm1.68\times10^{-5}$	$7.91\times10^{-5}\pm9.65\times10^{-6}$
PC O-38:6	$6.54\times10^{-4}\pm1.10\times10^{-4}$	$4.35\times10^{-4}\pm1.58\times10^{-4}$	$6.33\times10^{-4}\pm2.51\times10^{-4}$	$5.65\times10^{-4}\pm1.59\times10^{-4}$
PC O-38:5	$1.75\times10^{-3}\pm2.52\times10^{-4}$	$1.45\times10^{-3}\pm2.33\times10^{-4}$	$2.47\times10^{-3}\pm1.05\times10^{-3}$	$1.06\times10^{-3}\pm2.73\times10^{-4}$
PC O-38:3	$4.79\times10^{-5}\pm7.36\times10^{-6}$	$5.10\times10^{-5}\pm2.33\times10^{-5}$	$4.69\times10^{-5}\pm1.14\times10^{-5}$	$4.12\times10^{-5}\pm9.31\times10^{-6}$
PC O-37:7	$2.74\times10^{-4}\pm8.09\times10^{-5}$	$2.32\times10^{-4}\pm5.29\times10^{-5}$	$2.36\times10^{-4}\pm8.19\times10^{-5}$	$2.69\times10^{-4}\pm5.20\times10^{-5}$
PC O-36:5	$1.26\times10^{-4}\pm2.36\times10^{-5}$	$1.37\times10^{-4}\pm4.96\times10^{-5}$	$1.25\times10^{-4}\pm4.33\times10^{-5}$	$1.17\times10^{-4}\pm7.81\times10^{-6}$
PC O-36:4	$1.41\times10^{-3}\pm3.30\times10^{-4}$	$1.21\times10^{-3}\pm2.20\times10^{-4}$	$1.49\times10^{-3}\pm7.04\times10^{-4}$	$1.18\times10^{-3}\pm1.67\times10^{-4}$
PC O-36:3	$4.86\times10^{-5}\pm1.13\times10^{-5}$	$3.49\times10^{-5}\pm4.98\times10^{-6}$	$5.49\times10^{-5}\pm4.10\times10^{-5}$	$2.72\times10^{-5}\pm5.07\times10^{-6}$
PC O-36:2	$1.60\times10^{-4}\pm3.23\times10^{-5}$	$1.46\times10^{-4}\pm4.03\times10^{-5}$	$1.45\times10^{-4}\pm3.63\times10^{-5}$	$1.26\times10^{-4}\pm3.08\times10^{-5}$
PC O-36:0	$4.70\times10^{-5}\pm9.83\times10^{-6}$	$5.35\times10^{-5}\pm1.01\times10^{-5}$	$5.56\times10^{-5}\pm2.78\times10^{-5}$	$5.08\times10^{-5}\pm1.01\times10^{-5}$
PC O-35:7	$4.17\times10^{-4}\pm5.10\times10^{-5}$	$4.47\times10^{-4}\pm9.14\times10^{-5}$	$3.85\times10^{-4}\pm1.51\times10^{-4}$	$4.48\times10^{-4}\pm4.11\times10^{-5}$
PC O-35:2	$3.98\times10^{-8}\pm7.96\times10^{-8}$	$2.93\times10^{-5}\pm1.90\times10^{-5}$	$2.96\times10^{-6}\pm5.01\times10^{-6}$	$3.11\times10^{-6}\pm3.28\times10^{-6}$

PC O-34:3	$1.89\times10^{-5}\pm5.67\times10^{-6}$	$1.78\times10^{-5}\pm3.56\times10^{-6}$	$2.21\times10^{-5}\pm4.84\times10^{-6}$	$2.29\times10^{-5}\pm3.26\times10^{-6}$
PC O-34:2	$1.11\times10^{-4}\pm1.58\times10^{-5}$	$7.81\times10^{-5}\pm1.64\times10^{-5}$	$1.42\times10^{-4}\pm8.46\times10^{-5}$	$6.34\times10^{-5}\pm1.43\times10^{-5}$
PC O-34:1	$2.29\times10^{-3}\pm3.49\times10^{-4}$	$1.94\times10^{-3}\pm2.09\times10^{-4}$	$2.52\times10^{-3}\pm1.15\times10^{-3}$	$1.69\times10^{-3}\pm2.25\times10^{-4}$
PC O-34:0	$6.75\times10^{-4}\pm1.60\times10^{-4}$	$6.91\times10^{-4}\pm2.01\times10^{-4}$	$8.05\times10^{-4}\pm4.27\times10^{-4}$	$4.76\times10^{-4}\pm7.69\times10^{-5}$
PC O-33:6	$1.00\times10^{-5}\pm3.25\times10^{-6}$	$5.90\times10^{-6}\pm1.24\times10^{-6}$	$9.74\times10^{-6}\pm3.17\times10^{-6}$	$3.70\times10^{-6}\pm7.88\times10^{-7}$
PC O-33:2	$2.80\times10^{-6}\pm2.08\times10^{-6}$	$1.05\times10^{-5}\pm7.28\times10^{-6}$	$7.26\times10^{-6}\pm3.54\times10^{-6}$	$1.00\times10^{-5}\pm4.45\times10^{-6}$
PC O-32:2	$1.67\times10^{-5}\pm1.61\times10^{-6}$	$1.53\times10^{-5}\pm2.18\times10^{-6}$	$1.72\times10^{-5}\pm3.02\times10^{-6}$	$1.34\times10^{-5}\pm2.68\times10^{-6}$
PC O-32:1	$1.72\times10^{-4}\pm5.35\times10^{-5}$	$1.10\times10^{-4}\pm4.74\times10^{-5}$	$1.35\times10^{-4}\pm4.80\times10^{-5}$	$2.06\times10^{-4}\pm2.56\times10^{-5}$
PC O-32:0	$2.22\times10^{-3}\pm5.81\times10^{-4}$	$1.84\times10^{-3}\pm3.03\times10^{-4}$	$2.47\times10^{-3}\pm1.64\times10^{-3}$	$1.66\times10^{-3}\pm2.92\times10^{-4}$
PC O-31:0	$3.19\times10^{-6}\pm1.81\times10^{-6}$	$1.08\times10^{-6}\pm7.45\times10^{-7}$	$1.96\times10^{-6}\pm1.80\times10^{-6}$	$1.30\times10^{-6}\pm9.50\times10^{-7}$
PC O-30:0	$2.79\times10^{-5}\pm7.75\times10^{-6}$	$2.02\times10^{-5}\pm6.41\times10^{-6}$	$3.08\times10^{-5}\pm2.24\times10^{-5}$	$1.99\times10^{-5}\pm4.30\times10^{-6}$
PC 46:12	$4.30\times10^{-5}\pm7.70\times10^{-6}$	$3.29\times10^{-5}\pm9.44\times10^{-6}$	$3.04\times10^{-5}\pm1.17\times10^{-5}$	$2.86\times10^{-5}\pm7.93\times10^{-6}$
PC 44:7	$1.17\times10^{-5}\pm6.41\times10^{-6}$	$1.12\times10^{-5}\pm3.91\times10^{-6}$	$9.20\times10^{-6}\pm2.64\times10^{-6}$	$1.43\times10^{-5}\pm4.84\times10^{-6}$
PC 44:6	$2.13\times10^{-7}\pm4.25\times10^{-7}$	$7.99\times10^{-6}\pm5.93\times10^{-6}$	$8.39\times10^{-7}\pm1.68\times10^{-6}$	$5.38\times10^{-6}\pm5.37\times10^{-6}$
PC 44:4	-	$2.79\times10^{-6}\pm1.95\times10^{-6}$	-	$9.65\times10^{-8}\pm1.93\times10^{-7}$
PC 44:12|PC 22:6_22:6	$2.32\times10^{-3}\pm5.66\times10^{-4}$	$1.37\times10^{-3}\pm3.97\times10^{-4}$	$1.10\times10^{-3}\pm4.12\times10^{-4}$	$7.98\times10^{-4}\pm1.92\times10^{-4}$
PC 44:11	$8.15\times10^{-4}\pm2.59\times10^{-4}$	$4.32\times10^{-4}\pm1.66\times10^{-4}$	$4.12\times10^{-4}\pm2.61\times10^{-4}$	$3.36\times10^{-4}\pm1.76\times10^{-4}$
PC 44:10	$1.32\times10^{-4}\pm2.85\times10^{-5}$	$8.27\times10^{-5}\pm2.07\times10^{-5}$	$8.36\times10^{-5}\pm3.06\times10^{-5}$	$7.64\times10^{-5}\pm3.40\times10^{-5}$
PC 42:9	$1.96\times10^{-3}\pm2.53\times10^{-4}$	$1.86\times10^{-3}\pm1.10\times10^{-3}$	$1.30\times10^{-3}\pm4.16\times10^{-4}$	$9.64\times10^{-4}\pm2.13\times10^{-4}$
PC 42:8	$5.03\times10^{-4}\pm1.05\times10^{-4}$	$3.80\times10^{-4}\pm1.64\times10^{-4}$	$3.21\times10^{-4}\pm1.07\times10^{-4}$	$2.90\times10^{-4}\pm5.66\times10^{-5}$
PC 42:7	$1.67\times10^{-4}\pm2.34\times10^{-5}$	$1.64\times10^{-4}\pm4.99\times10^{-5}$	$1.78\times10^{-4}\pm4.25\times10^{-5}$	$2.18\times10^{-4}\pm4.21\times10^{-5}$
PC 42:6	$3.37\times10^{-5}\pm1.96\times10^{-5}$	$2.59\times10^{-5}\pm5.68\times10^{-6}$	$2.78\times10^{-5}\pm1.71\times10^{-5}$	$4.76\times10^{-5}\pm1.06\times10^{-5}$
PC 42:5	$2.53\times10^{-5}\pm1.16\times10^{-5}$	$3.77\times10^{-5}\pm1.16\times10^{-5}$	$2.36\times10^{-5}\pm5.49\times10^{-6}$	$2.87\times10^{-5}\pm6.66\times10^{-6}$
PC 42:4	$2.85\times10^{-5}\pm1.80\times10^{-5}$	$5.19\times10^{-5}\pm1.38\times10^{-5}$	$3.20\times10^{-5}\pm3.71\times10^{-6}$	$2.47\times10^{-5}\pm8.96\times10^{-6}$
PC 42:3	$1.99\times10^{-5}\pm6.81\times10^{-6}$	$2.20\times10^{-5}\pm9.76\times10^{-6}$	$1.94\times10^{-5}\pm5.43\times10^{-6}$	$2.16\times10^{-5}\pm5.34\times10^{-6}$
PC 42:2	$1.27\times10^{-5}\pm5.41\times10^{-6}$	$2.24\times10^{-5}\pm8.19\times10^{-6}$	$2.25\times10^{-5}\pm6.31\times10^{-6}$	$2.08\times10^{-5}\pm3.07\times10^{-6}$

PC 42:11	$1.85\times10^{-5}\pm4.32\times10^{-6}$	$2.63\times10^{-5}\pm8.75\times10^{-6}$	$1.72\times10^{-5}\pm5.37\times10^{-6}$	$1.74\times10^{-5}\pm9.45\times10^{-6}$
PC 42:10	$8.39\times10^{-3}\pm1.10\times10^{-3}$	$4.42\times10^{-3}\pm1.46\times10^{-3}$	$3.72\times10^{-3}\pm1.05\times10^{-3}$	$3.94\times10^{-3}\pm1.83\times10^{-3}$
PC 42:1	$1.19\times10^{-5}\pm4.27\times10^{-6}$	$1.83\times10^{-5}\pm4.28\times10^{-6}$	$1.81\times10^{-5}\pm6.37\times10^{-6}$	$1.04\times10^{-5}\pm2.62\times10^{-6}$
PC 41:7	$3.29\times10^{-5}\pm1.02\times10^{-5}$	$3.94\times10^{-5}\pm1.27\times10^{-5}$	$3.42\times10^{-5}\pm1.08\times10^{-5}$	$3.93\times10^{-5}\pm1.25\times10^{-5}$
PC 41:6	$5.61\times10^{-5}\pm1.37\times10^{-5}$	$6.85\times10^{-5}\pm3.65\times10^{-5}$	$5.01\times10^{-5}\pm9.94\times10^{-6}$	$7.35\times10^{-5}\pm1.01\times10^{-5}$
PC 41:5	$1.22\times10^{-5}\pm3.95\times10^{-6}$	$2.03\times10^{-5}\pm4.20\times10^{-6}$	$1.71\times10^{-5}\pm4.50\times10^{-6}$	$1.30\times10^{-5}\pm5.36\times10^{-6}$
PC 40:9	$5.69\times10^{-5}\pm9.47\times10^{-6}$	$6.10\times10^{-5}\pm1.60\times10^{-5}$	$4.76\times10^{-5}\pm7.08\times10^{-6}$	$5.65\times10^{-5}\pm1.51\times10^{-5}$
PC 40:8	$1.27\times10^{-2}\pm2.26\times10^{-3}$	$9.75\times10^{-3}\pm1.97\times10^{-3}$	$8.35\times10^{-3}\pm2.30\times10^{-3}$	$7.65\times10^{-3}\pm1.48\times10^{-3}$
PC 40:7	$1.12\times10^{-2}\pm2.88\times10^{-3}$	$1.22\times10^{-2}\pm1.06\times10^{-3}$	$8.61\times10^{-3}\pm1.28\times10^{-3}$	$1.10\times10^{-2}\pm2.84\times10^{-3}$
PC 40:6	$2.97\times10^{-2}\pm6.42\times10^{-3}$	$2.78\times10^{-2}\pm3.72\times10^{-3}$	$3.31\times10^{-2}\pm8.86\times10^{-3}$	$4.52\times10^{-2}\pm1.18\times10^{-2}$
PC 40:5	$2.39\times10^{-3}\pm8.24\times10^{-4}$	$2.16\times10^{-3}\pm8.31\times10^{-4}$	$1.78\times10^{-3}\pm3.99\times10^{-4}$	$3.00\times10^{-3}\pm7.93\times10^{-4}$
PC 40:4	$1.12\times10^{-4}\pm7.26\times10^{-5}$	$1.03\times10^{-4}\pm2.42\times10^{-5}$	$1.15\times10^{-4}\pm7.09\times10^{-5}$	$1.24\times10^{-4}\pm2.69\times10^{-5}$
PC 40:3	$1.77\times10^{-4}\pm7.07\times10^{-5}$	$2.10\times10^{-4}\pm4.45\times10^{-5}$	$1.98\times10^{-4}\pm3.80\times10^{-5}$	$1.78\times10^{-4}\pm2.92\times10^{-5}$
PC 40:2	$7.84\times10^{-5}\pm2.31\times10^{-5}$	$7.39\times10^{-5}\pm2.04\times10^{-5}$	$6.30\times10^{-5}\pm1.54\times10^{-5}$	$9.58\times10^{-5}\pm2.24\times10^{-5}$
PC 40:10	$2.26\times10^{-5}\pm5.72\times10^{-6}$	$2.88\times10^{-5}\pm7.56\times10^{-6}$	$2.31\times10^{-5}\pm9.08\times10^{-6}$	$3.14\times10^{-5}\pm7.25\times10^{-6}$
PC 40:1	$3.34\times10^{-5}\pm7.50\times10^{-6}$	$4.42\times10^{-5}\pm1.13\times10^{-5}$	$3.81\times10^{-5}\pm1.52\times10^{-5}$	$3.31\times10^{-5}\pm1.15\times10^{-5}$
PC 39:8	$2.78\times10^{-5}\pm4.15\times10^{-6}$	$3.11\times10^{-5}\pm1.46\times10^{-5}$	$2.83\times10^{-5}\pm4.92\times10^{-6}$	$3.05\times10^{-5}\pm7.76\times10^{-6}$
PC 39:7	$5.79\times10^{-5}\pm1.66\times10^{-5}$	$9.12\times10^{-5}\pm1.53\times10^{-5}$	$7.15\times10^{-5}\pm2.55\times10^{-5}$	$8.86\times10^{-5}\pm2.55\times10^{-5}$
PC 39:6	$1.46\times10^{-3}\pm3.85\times10^{-4}$	$1.83\times10^{-3}\pm6.36\times10^{-4}$	$1.11\times10^{-3}\pm1.95\times10^{-4}$	$2.00\times10^{-3}\pm5.02\times10^{-4}$
PC 39:5	$1.29\times10^{-5}\pm2.04\times10^{-5}$	$2.24\times10^{-5}\pm9.75\times10^{-6}$	$1.73\times10^{-5}\pm1.46\times10^{-5}$	$2.41\times10^{-5}\pm9.91\times10^{-6}$
PC 39:4	$2.71\times10^{-4}\pm9.10\times10^{-5}$	$3.47\times10^{-4}\pm1.01\times10^{-4}$	$2.69\times10^{-4}\pm1.02\times10^{-4}$	$3.16\times10^{-4}\pm1.04\times10^{-4}$
PC 39:3	$7.07\times10^{-5}\pm8.49\times10^{-6}$	$1.65\times10^{-4}\pm6.10\times10^{-5}$	$9.46\times10^{-5}\pm2.37\times10^{-5}$	$9.97\times10^{-5}\pm9.53\times10^{-6}$
PC 39:1	$1.76\times10^{-6}\pm2.60\times10^{-6}$	$7.83\times10^{-6}\pm4.07\times10^{-6}$	$3.06\times10^{-6}\pm2.91\times10^{-6}$	$2.43\times10^{-6}\pm1.68\times10^{-6}$
PC 38:7	$4.04\times10^{-4}\pm1.60\times10^{-4}$	$4.55\times10^{-4}\pm1.16\times10^{-4}$	$4.08\times10^{-4}\pm1.67\times10^{-4}$	$5.03\times10^{-4}\pm1.61\times10^{-4}$
PC 38:6	$6.61\times10^{-2}\pm1.56\times10^{-2}$	$4.39\times10^{-2}\pm7.20\times10^{-3}$	$4.75\times10^{-2}\pm1.42\times10^{-2}$	$4.82\times10^{-2}\pm1.85\times10^{-3}$
PC 38:5	$1.06\times10^{-2}\pm2.94\times10^{-3}$	$9.38\times10^{-3}\pm9.32\times10^{-4}$	$9.51\times10^{-3}\pm1.22\times10^{-3}$	$1.15\times10^{-2}\pm2.98\times10^{-3}$

PC 38:4	$2.98\times10^{-2}\pm1.05\times10^{-2}$	$2.58\times10^{-2}\pm4.08\times10^{-3}$	$2.27\times10^{-2}\pm5.37\times10^{-3}$	$2.86\times10^{-2}\pm4.93\times10^{-3}$
PC 38:3	$1.45\times10^{-2}\pm4.39\times10^{-3}$	$1.33\times10^{-2}\pm4.08\times10^{-3}$	$1.27\times10^{-2}\pm2.73\times10^{-3}$	$1.62\times10^{-2}\pm2.63\times10^{-3}$
PC 38:2	$3.87\times10^{-3}\pm1.23\times10^{-3}$	$5.06\times10^{-3}\pm8.16\times10^{-4}$	$3.71\times10^{-3}\pm6.80\times10^{-4}$	$3.96\times10^{-3}\pm1.15\times10^{-3}$
PC 38:1	$8.63\times10^{-4}\pm3.09\times10^{-4}$	$6.41\times10^{-4}\pm2.28\times10^{-4}$	$8.05\times10^{-4}\pm1.35\times10^{-4}$	$8.59\times10^{-4}\pm1.15\times10^{-4}$
PC 37:6	$2.16\times10^{-4}\pm8.94\times10^{-5}$	$3.70\times10^{-4}\pm1.07\times10^{-4}$	$2.85\times10^{-4}\pm9.78\times10^{-5}$	$3.40\times10^{-4}\pm8.81\times10^{-5}$
PC 37:5	$1.08\times10^{-4}\pm3.67\times10^{-5}$	$1.24\times10^{-4}\pm2.86\times10^{-5}$	$1.15\times10^{-4}\pm3.55\times10^{-5}$	$1.37\times10^{-4}\pm3.59\times10^{-5}$
PC 37:4	$1.55\times10^{-3}\pm6.11\times10^{-4}$	$1.58\times10^{-3}\pm2.80\times10^{-4}$	$1.55\times10^{-3}\pm6.51\times10^{-4}$	$1.58\times10^{-3}\pm5.65\times10^{-4}$
PC 37:3	$2.34\times10^{-4}\pm8.74\times10^{-5}$	$2.51\times10^{-4}\pm5.63\times10^{-5}$	$2.23\times10^{-4}\pm7.26\times10^{-5}$	$2.77\times10^{-4}\pm6.85\times10^{-5}$
PC 37:1	$2.39\times10^{-4}\pm7.29\times10^{-5}$	$2.34\times10^{-4}\pm8.69\times10^{-5}$	$2.51\times10^{-4}\pm3.47\times10^{-5}$	$3.67\times10^{-4}\pm4.95\times10^{-5}$
PC 37:0	$1.96\times10^{-5}\pm7.75\times10^{-6}$	$1.67\times10^{-5}\pm1.27\times10^{-5}$	$1.15\times10^{-5}\pm5.79\times10^{-6}$	$1.39\times10^{-5}\pm1.35\times10^{-6}$
PC 36:6	$6.51\times10^{-5}\pm1.53\times10^{-5}$	$1.28\times10^{-4}\pm4.47\times10^{-5}$	$7.55\times10^{-5}\pm2.34\times10^{-5}$	$9.96\times10^{-5}\pm3.73\times10^{-5}$
PC 36:5	$5.15\times10^{-4}\pm1.65\times10^{-4}$	$9.73\times10^{-4}\pm1.73\times10^{-4}$	$7.73\times10^{-4}\pm3.52\times10^{-4}$	$1.17\times10^{-3}\pm3.02\times10^{-4}$
PC 36:4	$3.15\times10^{-2}\pm1.00\times10^{-2}$	$3.16\times10^{-2}\pm3.74\times10^{-3}$	$3.25\times10^{-2}\pm4.39\times10^{-3}$	$3.76\times10^{-2}\pm3.09\times10^{-3}$
PC 36:3	$1.70\times10^{-2}\pm4.60\times10^{-3}$	$2.20\times10^{-2}\pm2.51\times10^{-3}$	$1.54\times10^{-2}\pm3.81\times10^{-3}$	$2.53\times10^{-2}\pm4.28\times10^{-3}$
PC 36:2;2O|PC 18:0_18:2;2O	$2.27\times10^{-6}\pm7.00\times10^{-7}$	$3.95\times10^{-6}\pm2.83\times10^{-6}$	$3.43\times10^{-6}\pm2.69\times10^{-6}$	$1.12\times10^{-6}\pm4.27\times10^{-7}$
PC 36:2	$5.04\times10^{-2}\pm1.20\times10^{-2}$	$4.85\times10^{-2}\pm8.71\times10^{-3}$	$4.63\times10^{-2}\pm1.01\times10^{-2}$	$8.09\times10^{-2}\pm1.57\times10^{-2}$
PC 36:1	$1.85\times10^{-2}\pm3.00\times10^{-3}$	$1.85\times10^{-2}\pm2.50\times10^{-3}$	$1.64\times10^{-2}\pm9.90\times10^{-4}$	$2.15\times10^{-2}\pm1.09\times10^{-3}$
PC 36:0|PC 18:0_18:0	$1.16\times10^{-3}\pm4.02\times10^{-4}$	$7.27\times10^{-4}\pm1.83\times10^{-4}$	$6.90\times10^{-4}\pm3.46\times10^{-4}$	$4.42\times10^{-4}\pm1.38\times10^{-4}$
PC 35:4	$2.37\times10^{-4}\pm1.09\times10^{-4}$	$3.09\times10^{-4}\pm7.67\times10^{-5}$	$2.80\times10^{-4}\pm1.17\times10^{-4}$	$3.35\times10^{-4}\pm8.95\times10^{-5}$
PC 35:3	$8.10\times10^{-5}\pm1.09\times10^{-5}$	$7.96\times10^{-5}\pm8.93\times10^{-6}$	$1.06\times10^{-4}\pm4.16\times10^{-5}$	$9.87\times10^{-5}\pm1.80\times10^{-5}$
PC 35:2	$2.07\times10^{-3}\pm6.41\times10^{-4}$	$2.86\times10^{-3}\pm4.35\times10^{-4}$	$2.69\times10^{-3}\pm8.88\times10^{-4}$	$2.72\times10^{-3}\pm1.16\times10^{-3}$
PC 35:1	$1.97\times10^{-3}\pm2.58\times10^{-4}$	$2.34\times10^{-3}\pm5.98\times10^{-4}$	$2.68\times10^{-3}\pm4.68\times10^{-4}$	$3.05\times10^{-3}\pm6.25\times10^{-4}$
PC 35:0	$2.27\times10^{-4}\pm5.22\times10^{-5}$	$1.84\times10^{-4}\pm5.19\times10^{-5}$	$1.82\times10^{-4}\pm5.57\times10^{-5}$	$1.34\times10^{-4}\pm2.09\times10^{-5}$
PC 34:4	$1.21\times10^{-4}\pm3.41\times10^{-5}$	$2.40\times10^{-4}\pm8.95\times10^{-5}$	$1.60\times10^{-4}\pm6.99\times10^{-5}$	$2.18\times10^{-4}\pm6.38\times10^{-5}$
PC 34:3	$1.04\times10^{-3}\pm2.51\times10^{-4}$	$1.12\times10^{-3}\pm1.60\times10^{-4}$	$1.38\times10^{-3}\pm3.70\times10^{-4}$	$1.09\times10^{-3}\pm3.62\times10^{-4}$
PC 34:2;2O|PC 16:0_18:2;2O	$1.45\times10^{-6}\pm3.46\times10^{-7}$	$5.52\times10^{-6}\pm5.20\times10^{-6}$	$3.73\times10^{-6}\pm3.54\times10^{-6}$	$1.23\times10^{-6}\pm5.48\times10^{-7}$

PC 34:2	$7.44\times10^{-2}\pm1.51\times10^{-2}$	$8.99\times10^{-2}\pm1.21\times10^{-2}$	$8.71\times10^{-2}\pm3.20\times10^{-2}$	$8.94\times10^{-2}\pm6.77\times10^{-3}$
PC 34:1	$5.22\times10^{-2}\pm1.28\times10^{-2}$	$4.96\times10^{-2}\pm1.04\times10^{-2}$	$5.81\times10^{-2}\pm6.67\times10^{-3}$	$5.16\times10^{-2}\pm1.29\times10^{-2}$
PC 34:0	$1.07\times10^{-2}\pm1.93\times10^{-3}$	$7.40\times10^{-3}\pm1.92\times10^{-3}$	$8.62\times10^{-3}\pm4.41\times10^{-3}$	$6.42\times10^{-3}\pm2.00\times10^{-3}$
PC 33:2	$7.03\times10^{-5}\pm5.97\times10^{-5}$	$2.17\times10^{-4}\pm7.19\times10^{-5}$	$2.00\times10^{-4}\pm1.32\times10^{-4}$	$2.37\times10^{-4}\pm1.35\times10^{-4}$
PC 33:1	$1.20\times10^{-3}\pm3.75\times10^{-4}$	$1.61\times10^{-3}\pm1.70\times10^{-4}$	$1.78\times10^{-3}\pm4.73\times10^{-4}$	$1.84\times10^{-3}\pm3.07\times10^{-4}$
PC 33:0	$6.53\times10^{-4}\pm8.68\times10^{-5}$	$4.47\times10^{-4}\pm9.94\times10^{-5}$	$5.90\times10^{-4}\pm2.22\times10^{-4}$	$4.39\times10^{-4}\pm1.36\times10^{-4}$
PC 32:2	$4.71\times10^{-4}\pm1.96\times10^{-4}$	$9.84\times10^{-4}\pm4.20\times10^{-4}$	$7.61\times10^{-4}\pm3.80\times10^{-4}$	$8.51\times10^{-4}\pm1.72\times10^{-4}$
PC 32:1	$1.36\times10^{-3}\pm3.23\times10^{-4}$	$1.40\times10^{-3}\pm2.45\times10^{-4}$	$1.61\times10^{-3}\pm3.74\times10^{-4}$	$1.53\times10^{-3}\pm4.75\times10^{-4}$
PC 32:0|PC 16:0_16:0	$3.35\times10^{-2}\pm2.30\times10^{-3}$	$2.63\times10^{-2}\pm4.59\times10^{-3}$	$3.20\times10^{-2}\pm1.64\times10^{-2}$	$1.92\times10^{-2}\pm3.99\times10^{-3}$
PC 31:1	$5.98\times10^{-7}\pm1.20\times10^{-6}$	$1.95\times10^{-6}\pm1.77\times10^{-6}$	$4.63\times10^{-6}\pm4.76\times10^{-6}$	$8.29\times10^{-6}\pm7.40\times10^{-6}$
PC 30:0	$2.05\times10^{-4}\pm6.24\times10^{-5}$	$2.42\times10^{-4}\pm7.26\times10^{-5}$	$2.71\times10^{-4}\pm1.82\times10^{-4}$	$2.77\times10^{-4}\pm1.36\times10^{-4}$
PC 28:0	$3.03\times10^{-6}\pm1.07\times10^{-6}$	$3.78\times10^{-6}\pm1.71\times10^{-6}$	$2.42\times10^{-6}\pm1.39\times10^{-6}$	$5.64\times10^{-6}\pm2.17\times10^{-6}$
PA 38:6|PA 16:0_22:6	$1.12\times10^{-5}\pm2.80\times10^{-6}$	$9.47\times10^{-6}\pm1.76\times10^{-6}$	$7.83\times10^{-6}\pm1.13\times10^{-6}$	$1.12\times10^{-5}\pm3.69\times10^{-6}$
PA 36:4|PA 18:2_18:2	$4.52\times10^{-6}\pm3.43\times10^{-7}$	$4.11\times10^{-6}\pm6.18\times10^{-7}$	$4.66\times10^{-6}\pm5.50\times10^{-7}$	$3.36\times10^{-6}\pm4.60\times10^{-7}$
PA 34:2|PA 16:0_18:2	$6.70\times10^{-7}\pm1.23\times10^{-6}$	$6.37\times10^{-7}\pm6.23\times10^{-7}$	$1.71\times10^{-5}\pm1.19\times10^{-5}$	$1.13\times10^{-7}\pm1.64\times10^{-7}$
PA 32:0|PA 16:0_16:0	$2.39\times10^{-6}\pm2.01\times10^{-6}$	$1.48\times10^{-7}\pm1.38\times10^{-7}$	$2.64\times10^{-6}\pm2.38\times10^{-6}$	$2.04\times10^{-6}\pm1.55\times10^{-6}$
Phenylalanine	$1.85\times10^{-3}\pm2.55\times10^{-4}$	$1.51\times10^{-3}\pm4.47\times10^{-4}$	$2.01\times10^{-3}\pm9.06\times10^{-4}$	$2.28\times10^{-3}\pm4.34\times10^{-4}$
Dodecylbenzenesulfonic acid	$7.84\times10^{-5}\pm1.32\times10^{-5}$	$1.44\times10^{-4}\pm6.40\times10^{-5}$	$1.07\times10^{-4}\pm2.96\times10^{-5}$	$4.34\times10^{-5}\pm2.19\times10^{-5}$
Diisodecyl phthalate (also known as the production of plastic)	$1.83\times10^{-5}\pm3.44\times10^{-6}$	$1.65\times10^{-5}\pm2.86\times10^{-6}$	$1.91\times10^{-5}\pm6.40\times10^{-6}$	$1.31\times10^{-5}\pm5.02\times10^{-7}$
Carnitine	$2.12\times10^{-3}\pm8.33\times10^{-4}$	$1.34\times10^{-3}\pm5.18\times10^{-4}$	$1.87\times10^{-3}\pm8.82\times10^{-4}$	$1.98\times10^{-3}\pm3.91\times10^{-4}$
BMP 40:8|BMP 18:2_22:6	$8.09\times10^{-6}\pm1.77\times10^{-6}$	$1.03\times10^{-5}\pm1.36\times10^{-6}$	$8.43\times10^{-6}\pm2.72\times10^{-6}$	$6.26\times10^{-6}\pm2.34\times10^{-6}$
LPS 19:0	$1.26\times10^{-5}\pm3.40\times10^{-6}$	$1.58\times10^{-5}\pm7.20\times10^{-6}$	$1.06\times10^{-5}\pm3.73\times10^{-6}$	$9.15\times10^{-6}\pm2.50\times10^{-6}$
LPS 18:0	$3.26\times10^{-4}\pm6.25\times10^{-5}$	$3.32\times10^{-4}\pm9.09\times10^{-5}$	$3.17\times10^{-4}\pm1.39\times10^{-4}$	$2.32\times10^{-4}\pm5.18\times10^{-5}$
LPS 16:0	$2.06\times10^{-5}\pm4.36\times10^{-6}$	$2.55\times10^{-5}\pm8.88\times10^{-6}$	$2.44\times10^{-5}\pm8.04\times10^{-6}$	$1.88\times10^{-5}\pm4.66\times10^{-6}$
LPI 22:6	$5.27\times10^{-5}\pm1.92\times10^{-5}$	$4.78\times10^{-5}\pm2.12\times10^{-5}$	$4.51\times10^{-5}\pm1.35\times10^{-5}$	$3.78\times10^{-5}\pm9.02\times10^{-6}$

LPI 22:5	$3.76\times10^{-5}\pm1.22\times10^{-5}$	$3.84\times10^{-5}\pm1.12\times10^{-5}$	$4.94\times10^{-5}\pm1.63\times10^{-5}$	$2.43\times10^{-5}\pm9.02\times10^{-6}$
LPI 22:4	$1.05\times10^{-5}\pm3.58\times10^{-6}$	$8.60\times10^{-6}\pm1.22\times10^{-6}$	$1.29\times10^{-5}\pm5.58\times10^{-6}$	$7.60\times10^{-6}\pm3.17\times10^{-6}$
LPI 20:4	$6.88\times10^{-4}\pm2.48\times10^{-4}$	$7.89\times10^{-4}\pm4.18\times10^{-4}$	$6.79\times10^{-4}\pm2.49\times10^{-4}$	$5.68\times10^{-4}\pm1.43\times10^{-4}$
LPI 20:3	$2.14\times10^{-4}\pm8.14\times10^{-5}$	$2.26\times10^{-4}\pm6.89\times10^{-5}$	$2.95\times10^{-4}\pm1.31\times10^{-4}$	$1.76\times10^{-4}\pm4.81\times10^{-5}$
LPI 20:2	$9.29\times10^{-6}\pm2.66\times10^{-6}$	$1.24\times10^{-5}\pm2.41\times10^{-6}$	$1.52\times10^{-5}\pm4.25\times10^{-6}$	$8.48\times10^{-6}\pm2.11\times10^{-6}$
LPI 20:0	$7.65\times10^{-6}\pm2.32\times10^{-6}$	$4.15\times10^{-6}\pm1.77\times10^{-6}$	$4.96\times10^{-6}\pm2.44\times10^{-6}$	$6.15\times10^{-6}\pm1.96\times10^{-6}$
LPI 19:0	$4.45\times10^{-5}\pm1.34\times10^{-5}$	$3.90\times10^{-5}\pm9.92\times10^{-6}$	$3.94\times10^{-5}\pm1.42\times10^{-5}$	$3.12\times10^{-5}\pm5.88\times10^{-6}$
LPI 18:2	$8.42\times10^{-5}\pm3.34\times10^{-5}$	$7.87\times10^{-5}\pm4.19\times10^{-5}$	$1.17\times10^{-4}\pm5.84\times10^{-5}$	$8.01\times10^{-5}\pm2.23\times10^{-5}$
LPI 18:1	$1.75\times10^{-4}\pm6.09\times10^{-5}$	$2.22\times10^{-4}\pm1.01\times10^{-4}$	$2.32\times10^{-4}\pm8.04\times10^{-5}$	$1.67\times10^{-4}\pm3.75\times10^{-5}$
LPI 18:0	$7.28\times10^{-3}\pm1.03\times10^{-3}$	$9.41\times10^{-3}\pm4.67\times10^{-3}$	$6.82\times10^{-3}\pm1.59\times10^{-3}$	$5.30\times10^{-3}\pm9.03\times10^{-4}$
LPI 17:0	$3.27\times10^{-5}\pm5.03\times10^{-6}$	$3.17\times10^{-5}\pm9.38\times10^{-6}$	$3.68\times10^{-5}\pm8.16\times10^{-6}$	$2.83\times10^{-5}\pm4.33\times10^{-6}$
LPI 16:0	$3.14\times10^{-4}\pm4.45\times10^{-5}$	$2.72\times10^{-4}\pm3.65\times10^{-5}$	$3.38\times10^{-4}\pm6.34\times10^{-5}$	$2.66\times10^{-4}\pm4.78\times10^{-5}$
LPG 22:6	$1.16\times10^{-4}\pm4.46\times10^{-5}$	$8.83\times10^{-5}\pm2.10\times10^{-5}$	$8.30\times10^{-5}\pm3.29\times10^{-5}$	$7.48\times10^{-5}\pm1.59\times10^{-5}$
LPG 22:5	$9.51\times10^{-6}\pm1.80\times10^{-6}$	$1.02\times10^{-5}\pm2.98\times10^{-6}$	$9.86\times10^{-6}\pm5.06\times10^{-6}$	$6.96\times10^{-6}\pm2.93\times10^{-6}$
LPG 20:4	$6.18\times10^{-5}\pm1.84\times10^{-5}$	$7.26\times10^{-5}\pm5.38\times10^{-5}$	$5.35\times10^{-5}\pm1.47\times10^{-5}$	$4.92\times10^{-5}\pm1.08\times10^{-5}$
LPG 20:3	$3.80\times10^{-5}\pm1.71\times10^{-5}$	$6.45\times10^{-5}\pm4.55\times10^{-5}$	$5.71\times10^{-5}\pm3.39\times10^{-5}$	$3.13\times10^{-5}\pm8.31\times10^{-6}$
LPG 20:2	$2.92\times10^{-5}\pm9.43\times10^{-6}$	$3.25\times10^{-5}\pm2.27\times10^{-5}$	$3.92\times10^{-5}\pm1.67\times10^{-5}$	$2.61\times10^{-5}\pm4.21\times10^{-6}$
LPG 19:0	$1.07\times10^{-5}\pm3.49\times10^{-6}$	$1.25\times10^{-5}\pm8.41\times10^{-6}$	$7.33\times10^{-6}\pm2.75\times10^{-6}$	$5.01\times10^{-6}\pm1.28\times10^{-6}$
LPG 18:2	$5.98\times10^{-4}\pm2.52\times10^{-4}$	$7.97\times10^{-4}\pm6.20\times10^{-4}$	$7.79\times10^{-4}\pm3.55\times10^{-4}$	$5.33\times10^{-4}\pm1.10\times10^{-4}$
LPG 18:1	$1.52\times10^{-4}\pm4.42\times10^{-5}$	$2.27\times10^{-4}\pm1.47\times10^{-4}$	$2.18\times10^{-4}\pm9.40\times10^{-5}$	$1.53\times10^{-4}\pm1.54\times10^{-5}$
LPG 18:0	$1.97\times10^{-4}\pm3.15\times10^{-5}$	$1.89\times10^{-4}\pm6.82\times10^{-5}$	$1.46\times10^{-4}\pm5.95\times10^{-5}$	$9.08\times10^{-5}\pm3.13\times10^{-5}$
LPG 16:1	$1.40\times10^{-5}\pm8.89\times10^{-6}$	$1.59\times10^{-5}\pm1.44\times10^{-5}$	$1.31\times10^{-5}\pm8.51\times10^{-6}$	$9.96\times10^{-6}\pm3.15\times10^{-6}$
LPG 16:0	$1.88\times10^{-4}\pm2.31\times10^{-5}$	$2.54\times10^{-4}\pm1.16\times10^{-4}$	$1.56\times10^{-4}\pm3.43\times10^{-5}$	$1.29\times10^{-4}\pm2.24\times10^{-5}$
LPE O-20:1	$6.75\times10^{-6}\pm1.60\times10^{-6}$	$6.61\times10^{-6}\pm2.02\times10^{-6}$	$4.49\times10^{-6}\pm3.23\times10^{-6}$	$5.37\times10^{-6}\pm1.77\times10^{-6}$
LPE O-18:2	$7.93\times10^{-5}\pm2.07\times10^{-5}$	$4.59\times10^{-5}\pm1.82\times10^{-5}$	$5.99\times10^{-5}\pm6.52\times10^{-5}$	$3.60\times10^{-5}\pm1.61\times10^{-5}$
LPE O-18:1	$1.04\times10^{-4}\pm3.00\times10^{-5}$	$6.28\times10^{-5}\pm1.64\times10^{-5}$	$4.90\times10^{-5}\pm2.42\times10^{-5}$	$4.39\times10^{-5}\pm1.05\times10^{-5}$

LPE O-16:1	$1.44\times10^{-4}\pm3.31\times10^{-5}$	$8.70\times10^{-5}\pm2.62\times10^{-5}$	$1.10\times10^{-4}\pm1.02\times10^{-4}$	$5.57\times10^{-5}\pm1.44\times10^{-5}$
LPE 22:6	$2.07\times10^{-3}\pm3.41\times10^{-4}$	$2.69\times10^{-3}\pm5.94\times10^{-4}$	$2.67\times10^{-3}\pm6.12\times10^{-4}$	$2.66\times10^{-3}\pm4.22\times10^{-4}$
LPE 22:5	$1.31\times10^{-4}\pm1.54\times10^{-5}$	$2.11\times10^{-4}\pm7.66\times10^{-5}$	$2.31\times10^{-4}\pm8.02\times10^{-5}$	$1.49\times10^{-4}\pm5.72\times10^{-5}$
LPE 22:4	$3.42\times10^{-5}\pm4.61\times10^{-6}$	$4.45\times10^{-5}\pm8.93\times10^{-6}$	$5.96\times10^{-5}\pm1.83\times10^{-5}$	$4.53\times10^{-5}\pm1.35\times10^{-5}$
LPE 22:1	$3.14\times10^{-6}\pm6.58\times10^{-7}$	$3.87\times10^{-6}\pm2.97\times10^{-7}$	$3.57\times10^{-6}\pm6.07\times10^{-7}$	$4.00\times10^{-6}\pm8.17\times10^{-7}$
LPE 22:0	$1.30\times10^{-6}\pm3.32\times10^{-7}$	$1.44\times10^{-6}\pm4.90\times10^{-7}$	$1.30\times10^{-6}\pm2.06\times10^{-7}$	$1.17\times10^{-6}\pm1.60\times10^{-7}$
LPE 20:4	$1.03\times10^{-3}\pm1.89\times10^{-4}$	$1.53\times10^{-3}\pm6.94\times10^{-4}$	$1.59\times10^{-3}\pm4.75\times10^{-4}$	$1.27\times10^{-3}\pm1.85\times10^{-4}$
LPE 20:3	$1.49\times10^{-4}\pm3.67\times10^{-5}$	$2.80\times10^{-4}\pm1.23\times10^{-4}$	$2.83\times10^{-4}\pm1.28\times10^{-4}$	$2.00\times10^{-4}\pm3.81\times10^{-5}$
LPE 20:2	$4.17\times10^{-5}\pm1.29\times10^{-5}$	$5.48\times10^{-5}\pm8.86\times10^{-6}$	$7.88\times10^{-5}\pm2.30\times10^{-5}$	$5.89\times10^{-5}\pm3.69\times10^{-6}$
LPE 20:1	$7.74\times10^{-5}\pm1.79\times10^{-5}$	$1.01\times10^{-4}\pm1.60\times10^{-5}$	$1.12\times10^{-4}\pm2.59\times10^{-5}$	$1.04\times10^{-4}\pm1.16\times10^{-5}$
LPE 20:0	$2.40\times10^{-5}\pm9.21\times10^{-6}$	$1.67\times10^{-5}\pm7.78\times10^{-6}$	$2.37\times10^{-5}\pm6.49\times10^{-6}$	$2.68\times10^{-5}\pm6.18\times10^{-6}$
LPE 19:0	$2.47\times10^{-5}\pm3.70\times10^{-6}$	$2.17\times10^{-5}\pm5.01\times10^{-6}$	$2.23\times10^{-5}\pm6.08\times10^{-6}$	$2.33\times10^{-5}\pm6.24\times10^{-6}$
LPE 18:3	$9.28\times10^{-6}\pm1.82\times10^{-6}$	$2.04\times10^{-5}\pm5.94\times10^{-6}$	$2.14\times10^{-5}\pm5.15\times10^{-6}$	$1.88\times10^{-5}\pm3.21\times10^{-6}$
LPE 18:2	$9.79\times10^{-4}\pm3.26\times10^{-4}$	$1.05\times10^{-3}\pm4.76\times10^{-4}$	$1.55\times10^{-3}\pm5.27\times10^{-4}$	$1.31\times10^{-3}\pm2.22\times10^{-4}$
LPE 18:1	$6.85\times10^{-3}\pm6.40\times10^{-3}$	$1.99\times10^{-3}\pm9.48\times10^{-4}$	$1.85\times10^{-3}\pm5.47\times10^{-4}$	$2.12\times10^{-2}\pm3.07\times10^{-2}$
LPE 18:0	$7.15\times10^{-3}\pm1.84\times10^{-3}$	$8.55\times10^{-3}\pm1.28\times10^{-3}$	$7.50\times10^{-3}\pm2.92\times10^{-3}$	$7.54\times10^{-3}\pm2.08\times10^{-3}$
LPE 17:0	$1.57\times10^{-4}\pm2.28\times10^{-5}$	$1.79\times10^{-4}\pm2.69\times10^{-5}$	$2.11\times10^{-4}\pm3.59\times10^{-5}$	$1.79\times10^{-4}\pm2.31\times10^{-5}$
LPE 16:1	$4.54\times10^{-5}\pm1.78\times10^{-5}$	$4.21\times10^{-5}\pm1.91\times10^{-5}$	$5.36\times10^{-5}\pm1.42\times10^{-5}$	$5.92\times10^{-5}\pm1.91\times10^{-5}$
LPE 16:0	$2.76\times10^{-3}\pm3.53\times10^{-4}$	$2.97\times10^{-3}\pm2.80\times10^{-4}$	$3.20\times10^{-3}\pm4.95\times10^{-4}$	$2.90\times10^{-3}\pm1.35\times10^{-4}$
LPC O-24:1	$8.01\times10^{-6}\pm8.50\times10^{-7}$	$6.27\times10^{-6}\pm1.67\times10^{-6}$	$8.58\times10^{-6}\pm3.39\times10^{-6}$	$4.78\times10^{-6}\pm5.49\times10^{-7}$
LPC O-24:0	$1.44\times10^{-5}\pm1.27\times10^{-6}$	$1.10\times10^{-5}\pm4.60\times10^{-6}$	$1.41\times10^{-5}\pm3.99\times10^{-6}$	$1.15\times10^{-5}\pm1.07\times10^{-6}$
LPC O-22:1	$1.24\times10^{-5}\pm2.51\times10^{-6}$	$1.03\times10^{-5}\pm3.13\times10^{-6}$	$1.05\times10^{-5}\pm8.58\times10^{-6}$	$6.50\times10^{-6}\pm1.42\times10^{-6}$
LPC O-22:0	$1.61\times10^{-5}\pm1.52\times10^{-6}$	$1.39\times10^{-5}\pm4.28\times10^{-6}$	$1.72\times10^{-5}\pm6.74\times10^{-6}$	$1.16\times10^{-5}\pm5.30\times10^{-7}$
LPC O-20:2	$7.13\times10^{-5}\pm2.07\times10^{-5}$	$2.55\times10^{-5}\pm5.97\times10^{-6}$	$4.30\times10^{-5}\pm3.68\times10^{-5}$	$1.85\times10^{-5}\pm4.44\times10^{-6}$
LPC O-20:0	$6.13\times10^{-5}\pm1.26\times10^{-5}$	$4.85\times10^{-5}\pm1.49\times10^{-5}$	$5.62\times10^{-5}\pm2.78\times10^{-5}$	$3.33\times10^{-5}\pm7.84\times10^{-6}$
LPC O-18:2	$8.50\times10^{-5}\pm2.81\times10^{-5}$	$2.85\times10^{-5}\pm9.18\times10^{-6}$	$5.43\times10^{-5}\pm6.04\times10^{-5}$	$2.05\times10^{-5}\pm4.22\times10^{-6}$

LPC O-18:1	$2.59\times10^{-3}\pm7.46\times10^{-4}$	$1.00\times10^{-3}\pm3.88\times10^{-4}$	$1.86\times10^{-3}\pm1.93\times10^{-3}$	$7.96\times10^{-4}\pm2.22\times10^{-4}$
LPC O-18:0	$2.87\times10^{-4}\pm6.86\times10^{-5}$	$1.85\times10^{-4}\pm6.60\times10^{-5}$	$2.32\times10^{-4}\pm1.59\times10^{-4}$	$1.28\times10^{-4}\pm3.68\times10^{-5}$
LPC O-16:0	$1.75\times10^{-3}\pm4.35\times10^{-4}$	$1.02\times10^{-3}\pm1.90\times10^{-4}$	$1.43\times10^{-3}\pm1.07\times10^{-3}$	$7.08\times10^{-4}\pm1.77\times10^{-4}$
LPC 24:6	$9.26\times10^{-5}\pm2.09\times10^{-5}$	$1.01\times10^{-4}\pm3.75\times10^{-5}$	$1.39\times10^{-4}\pm3.37\times10^{-5}$	$1.35\times10^{-4}\pm4.74\times10^{-5}$
LPC 24:5/0:0	$2.03\times10^{-5}\pm3.14\times10^{-6}$	$3.02\times10^{-5}\pm9.32\times10^{-6}$	$3.73\times10^{-5}\pm6.65\times10^{-6}$	$3.09\times10^{-5}\pm5.05\times10^{-6}$
LPC 24:2/0:0	$2.44\times10^{-6}\pm5.07\times10^{-7}$	$3.02\times10^{-6}\pm6.42\times10^{-7}$	$3.84\times10^{-6}\pm6.58\times10^{-7}$	$2.68\times10^{-6}\pm2.08\times10^{-7}$
LPC 24:1/0:0	$1.00\times10^{-5}\pm3.37\times10^{-6}$	$1.32\times10^{-5}\pm1.95\times10^{-6}$	$1.58\times10^{-5}\pm5.28\times10^{-6}$	$8.87\times10^{-6}\pm1.56\times10^{-6}$
LPC 24:0/0:0	$2.67\times10^{-5}\pm3.80\times10^{-6}$	$3.10\times10^{-5}\pm3.98\times10^{-6}$	$3.23\times10^{-5}\pm7.79\times10^{-6}$	$2.31\times10^{-5}\pm3.35\times10^{-6}$
LPC 23:0/0:0	$3.41\times10^{-6}\pm1.29\times10^{-7}$	$3.62\times10^{-6}\pm5.44\times10^{-7}$	$4.54\times10^{-6}\pm9.31\times10^{-7}$	$3.73\times10^{-6}\pm5.29\times10^{-7}$
LPC 22:6/0:0	$3.90\times10^{-2}\pm8.97\times10^{-3}$	$3.12\times10^{-2}\pm7.49\times10^{-3}$	$3.38\times10^{-2}\pm4.26\times10^{-3}$	$2.98\times10^{-2}\pm4.80\times10^{-3}$
LPC 22:5/0:0	$3.77\times10^{-3}\pm7.74\times10^{-4}$	$3.41\times10^{-3}\pm1.11\times10^{-3}$	$4.21\times10^{-3}\pm1.37\times10^{-3}$	$3.08\times10^{-3}\pm1.18\times10^{-3}$
LPC 22:4/0:0	$3.04\times10^{-4}\pm9.34\times10^{-5}$	$3.75\times10^{-4}\pm1.32\times10^{-4}$	$4.28\times10^{-4}\pm7.86\times10^{-5}$	$3.58\times10^{-4}\pm1.07\times10^{-4}$
LPC 22:3/0:0	$4.86\times10^{-5}\pm1.08\times10^{-5}$	$4.99\times10^{-5}\pm1.12\times10^{-5}$	$6.04\times10^{-5}\pm2.00\times10^{-5}$	$4.68\times10^{-5}\pm1.31\times10^{-5}$
LPC 22:2/0:0	$8.81\times10^{-6}\pm1.02\times10^{-6}$	$9.80\times10^{-6}\pm1.66\times10^{-6}$	$1.16\times10^{-5}\pm3.38\times10^{-6}$	$8.79\times10^{-6}\pm8.17\times10^{-7}$
LPC 22:1/0:0	$1.95\times10^{-5}\pm4.47\times10^{-6}$	$2.56\times10^{-5}\pm3.89\times10^{-6}$	$2.52\times10^{-5}\pm4.54\times10^{-6}$	$1.91\times10^{-5}\pm9.98\times10^{-7}$
LPC 22:0/0:0	$2.68\times10^{-5}\pm4.35\times10^{-6}$	$3.29\times10^{-5}\pm4.04\times10^{-6}$	$3.73\times10^{-5}\pm8.29\times10^{-6}$	$2.56\times10^{-5}\pm1.72\times10^{-6}$
LPC 21:0/0:0	$2.33\times10^{-6}\pm3.32\times10^{-7}$	$2.97\times10^{-6}\pm9.28\times10^{-7}$	$3.44\times10^{-6}\pm9.54\times10^{-7}$	$2.32\times10^{-6}\pm3.75\times10^{-7}$
LPC 20:5	$4.44\times10^{-4}\pm1.06\times10^{-4}$	$7.69\times10^{-4}\pm2.06\times10^{-4}$	$9.00\times10^{-4}\pm2.42\times10^{-4}$	$7.15\times10^{-4}\pm1.57\times10^{-4}$
LPC 20:4/0:0	$2.24\times10^{-2}\pm4.45\times10^{-3}$	$2.22\times10^{-2}\pm6.52\times10^{-3}$	$2.46\times10^{-2}\pm4.78\times10^{-3}$	$2.22\times10^{-2}\pm3.39\times10^{-3}$
LPC 20:3/0:0	$1.02\times10^{-2}\pm3.00\times10^{-3}$	$1.16\times10^{-2}\pm4.93\times10^{-3}$	$1.28\times10^{-2}\pm4.99\times10^{-3}$	$1.15\times10^{-2}\pm2.29\times10^{-3}$
LPC 20:2/0:0	$1.50\times10^{-3}\pm3.62\times10^{-4}$	$1.98\times10^{-3}\pm5.49\times10^{-4}$	$2.42\times10^{-3}\pm9.24\times10^{-4}$	$1.96\times10^{-3}\pm3.72\times10^{-4}$
LPC 20:1	$1.10\times10^{-4}\pm2.59\times10^{-5}$	$9.89\times10^{-5}\pm2.27\times10^{-5}$	$1.33\times10^{-4}\pm6.10\times10^{-5}$	$7.74\times10^{-5}\pm8.87\times10^{-6}$
LPC 20:0/0:0	$1.65\times10^{-4}\pm8.15\times10^{-5}$	$1.42\times10^{-4}\pm6.08\times10^{-5}$	$1.68\times10^{-4}\pm4.22\times10^{-5}$	$1.60\times10^{-4}\pm3.30\times10^{-5}$
LPC 19:1	$1.12\times10^{-4}\pm2.72\times10^{-5}$	$1.50\times10^{-4}\pm3.42\times10^{-5}$	$1.92\times10^{-4}\pm7.66\times10^{-5}$	$1.34\times10^{-4}\pm2.82\times10^{-5}$
LPC 19:0	$1.76\times10^{-5}\pm2.57\times10^{-6}$	$2.79\times10^{-5}\pm5.85\times10^{-6}$	$2.16\times10^{-5}\pm3.22\times10^{-6}$	$1.69\times10^{-5}\pm1.52\times10^{-6}$
LPC 18:3/0:0	$3.62\times10^{-4}\pm7.54\times10^{-5}$	$4.16\times10^{-4}\pm2.75\times10^{-4}$	$4.88\times10^{-4}\pm1.45\times10^{-4}$	$3.44\times10^{-4}\pm1.32\times10^{-4}$

LPC 18:2	$3.02\times10^{-2}\pm9.22\times10^{-3}$	$3.25\times10^{-2}\pm1.15\times10^{-2}$	$4.10\times10^{-2}\pm1.19\times10^{-2}$	$3.88\times10^{-2}\pm5.60\times10^{-3}$
LPC 18:1/0:0	$2.29\times10^{-2}\pm4.20\times10^{-3}$	$2.66\times10^{-2}\pm4.62\times10^{-3}$	$3.69\times10^{-2}\pm8.83\times10^{-3}$	$2.85\times10^{-2}\pm4.59\times10^{-3}$
LPC 18:0	$3.75\times10^{-3}\pm4.73\times10^{-4}$	$4.06\times10^{-3}\pm8.28\times10^{-4}$	$3.38\times10^{-3}\pm5.90\times10^{-4}$	$3.23\times10^{-3}\pm5.07\times10^{-4}$
LPC 17:1/0:0	$1.68\times10^{-4}\pm3.93\times10^{-5}$	$1.82\times10^{-4}\pm5.92\times10^{-5}$	$2.20\times10^{-4}\pm8.61\times10^{-5}$	$1.86\times10^{-4}\pm1.99\times10^{-5}$
LPC 17:0	$2.74\times10^{-4}\pm7.94\times10^{-5}$	$3.20\times10^{-4}\pm5.61\times10^{-5}$	$3.24\times10^{-4}\pm7.71\times10^{-5}$	$2.63\times10^{-4}\pm1.65\times10^{-5}$
LPC 16:1/0:0	$9.35\times10^{-4}\pm2.73\times10^{-4}$	$8.09\times10^{-4}\pm2.18\times10^{-4}$	$1.07\times10^{-3}\pm2.87\times10^{-4}$	$8.22\times10^{-4}\pm1.04\times10^{-4}$
LPC 16:0/0:0	$6.78\times10^{-2}\pm7.03\times10^{-3}$	$6.40\times10^{-2}\pm5.41\times10^{-3}$	$7.46\times10^{-2}\pm8.34\times10^{-3}$	$6.33\times10^{-2}\pm9.55\times10^{-3}$
LPC 15:0/0:0	$2.32\times10^{-4}\pm2.20\times10^{-5}$	$2.58\times10^{-4}\pm2.38\times10^{-5}$	$3.03\times10^{-4}\pm9.54\times10^{-5}$	$2.36\times10^{-4}\pm2.43\times10^{-5}$
LPC 14:0/0:0	$1.46\times10^{-4}\pm3.46\times10^{-5}$	$1.51\times10^{-4}\pm3.35\times10^{-5}$	$1.64\times10^{-4}\pm6.36\times10^{-5}$	$1.45\times10^{-4}\pm2.10\times10^{-5}$
FAHFA 25:6|FAHFA 22:6/3:0	$6.89\times10^{-5}\pm1.90\times10^{-5}$	$6.13\times10^{-5}\pm4.40\times10^{-5}$	$5.26\times10^{-5}\pm3.43\times10^{-5}$	$3.66\times10^{-5}\pm1.08\times10^{-5}$
FAHFA 25:5|FAHFA 22:5/3:0	$3.46\times10^{-6}\pm1.16\times10^{-6}$	$2.62\times10^{-6}\pm1.48\times10^{-6}$	$3.49\times10^{-6}\pm3.47\times10^{-6}$	$1.80\times10^{-6}\pm2.86\times10^{-7}$
FAHFA 23:4|FAHFA 20:4/3:0	$7.11\times10^{-5}\pm2.33\times10^{-5}$	$6.96\times10^{-5}\pm3.83\times10^{-5}$	$6.96\times10^{-5}\pm6.36\times10^{-5}$	$3.04\times10^{-5}\pm1.37\times10^{-5}$
FAHFA 23:3|FAHFA 20:3/3:0	$3.29\times10^{-5}\pm1.25\times10^{-5}$	$3.18\times10^{-5}\pm1.73\times10^{-5}$	$2.88\times10^{-5}\pm2.16\times10^{-5}$	$1.61\times10^{-5}\pm5.58\times10^{-6}$
FAHFA 21:2|FAHFA 18:2/3:0	$3.06\times10^{-4}\pm8.06\times10^{-5}$	$3.05\times10^{-4}\pm2.94\times10^{-4}$	$2.06\times10^{-4}\pm1.42\times10^{-4}$	$1.11\times10^{-4}\pm3.83\times10^{-5}$
FAHFA 21:1|FAHFA 18:1/3:0	$2.08\times10^{-4}\pm8.23\times10^{-5}$	$1.43\times10^{-4}\pm9.98\times10^{-5}$	$1.14\times10^{-4}\pm6.15\times10^{-5}$	$9.21\times10^{-5}\pm4.39\times10^{-5}$
FAHFA 20:1|FAHFA 18:1/2:0	$1.08\times10^{-5}\pm1.01\times10^{-5}$	$2.12\times10^{-5}\pm2.33\times10^{-5}$	$6.42\times10^{-6}\pm3.32\times10^{-6}$	$9.64\times10^{-6}\pm1.29\times10^{-5}$
FAHFA 20:0|FAHFA 18:0/2:0	$4.90\times10^{-5}\pm3.57\times10^{-5}$	$9.95\times10^{-5}\pm1.11\times10^{-4}$	$4.94\times10^{-5}\pm3.48\times10^{-5}$	$3.45\times10^{-5}\pm3.49\times10^{-5}$
FAHFA 18:0|FAHFA 16:0/2:0	$9.77\times10^{-5}\pm8.09\times10^{-5}$	$1.76\times10^{-4}\pm1.86\times10^{-4}$	$1.11\times10^{-4}\pm9.15\times10^{-5}$	$4.50\times10^{-5}\pm4.17\times10^{-5}$
FA 28:6	$1.07\times10^{-5}\pm1.55\times10^{-6}$	$1.01\times10^{-5}\pm2.21\times10^{-6}$	$1.06\times10^{-5}\pm7.71\times10^{-6}$	$8.31\times10^{-6}\pm3.14\times10^{-6}$
FA 24:5	$1.62\times10^{-6}\pm4.05\times10^{-7}$	$9.20\times10^{-7}\pm5.78\times10^{-7}$	$5.84\times10^{-7}\pm1.35\times10^{-7}$	$8.35\times10^{-7}\pm3.42\times10^{-7}$
FA 22:6	$1.71\times10^{-3}\pm4.67\times10^{-4}$	$1.19\times10^{-3}\pm3.65\times10^{-4}$	$1.20\times10^{-3}\pm6.84\times10^{-4}$	$9.29\times10^{-4}\pm2.10\times10^{-4}$
FA 22:5	$1.85\times10^{-5}\pm3.27\times10^{-6}$	$1.23\times10^{-5}\pm6.17\times10^{-6}$	$1.22\times10^{-5}\pm5.66\times10^{-6}$	$1.25\times10^{-5}\pm1.11\times10^{-6}$
FA 22:4	$2.96\times10^{-5}\pm8.44\times10^{-6}$	$1.26\times10^{-5}\pm1.83\times10^{-6}$	$1.75\times10^{-5}\pm9.45\times10^{-6}$	$9.67\times10^{-6}\pm2.60\times10^{-6}$
FA 20:5	$2.39\times10^{-5}\pm7.89\times10^{-6}$	$2.96\times10^{-5}\pm1.87\times10^{-5}$	$2.10\times10^{-5}\pm5.28\times10^{-6}$	$2.20\times10^{-5}\pm5.53\times10^{-6}$
FA 20:4	$1.15\times10^{-3}\pm2.48\times10^{-4}$	$9.46\times10^{-4}\pm3.09\times10^{-4}$	$8.39\times10^{-4}\pm5.04\times10^{-4}$	$5.47\times10^{-4}\pm1.55\times10^{-4}$
FA 20:3	$9.25\times10^{-5}\pm2.79\times10^{-5}$	$7.05\times10^{-5}\pm2.44\times10^{-5}$	$7.84\times10^{-5}\pm2.61\times10^{-5}$	$5.04\times10^{-5}\pm1.52\times10^{-5}$

FA 20:2	$4.16\times10^{-6}\pm1.30\times10^{-6}$	$5.14\times10^{-6}\pm2.23\times10^{-6}$	$2.35\times10^{-6}\pm1.12\times10^{-6}$	$3.01\times10^{-6}\pm4.94\times10^{-7}$
FA 20:0	$6.96\times10^{-8}\pm3.26\times10^{-8}$	$4.00\times10^{-8}\pm7.06\times10^{-8}$	$9.94\times10^{-9}\pm3.82\times10^{-9}$	$1.07\times10^{-7}\pm2.57\times10^{-8}$
FA 18:2	$3.66\times10^{-4}\pm8.38\times10^{-5}$	$3.24\times10^{-4}\pm1.76\times10^{-4}$	$2.46\times10^{-4}\pm9.01\times10^{-5}$	$1.79\times10^{-4}\pm3.74\times10^{-5}$
FA 18:1	$4.06\times10^{-4}\pm1.14\times10^{-4}$	$2.58\times10^{-4}\pm1.20\times10^{-4}$	$2.76\times10^{-4}\pm7.48\times10^{-5}$	$2.25\times10^{-4}\pm6.09\times10^{-5}$
FA 18:0	$1.28\times10^{-4}\pm2.43\times10^{-5}$	$9.43\times10^{-5}\pm3.45\times10^{-5}$	$1.01\times10^{-4}\pm4.20\times10^{-5}$	$7.32\times10^{-5}\pm1.60\times10^{-5}$
FA 16:0	$1.39\times10^{-4}\pm3.13\times10^{-5}$	$1.24\times10^{-4}\pm5.13\times10^{-5}$	$1.30\times10^{-4}\pm3.84\times10^{-5}$	$8.54\times10^{-5}\pm1.76\times10^{-5}$
DG 52:3|DG 34:1_18:2	$3.84\times10^{-5}\pm1.78\times10^{-5}$	$5.44\times10^{-5}\pm1.34\times10^{-5}$	$2.98\times10^{-5}\pm1.26\times10^{-5}$	$3.03\times10^{-5}\pm1.39\times10^{-5}$
DG 44:6|DG 18:0_26:6	$7.74\times10^{-4}\pm4.34\times10^{-4}$	$6.66\times10^{-4}\pm1.62\times10^{-4}$	$8.82\times10^{-4}\pm1.46\times10^{-4}$	$1.04\times10^{-3}\pm2.62\times10^{-4}$
DG 42:7|DG 18:1_24:6	$3.83\times10^{-5}\pm1.47\times10^{-5}$	$3.52\times10^{-5}\pm6.03\times10^{-6}$	$4.79\times10^{-5}\pm1.06\times10^{-5}$	$5.25\times10^{-5}\pm1.23\times10^{-5}$
DG 40:7|DG 18:2_22:5	$3.74\times10^{-6}\pm2.66\times10^{-6}$	$5.98\times10^{-7}\pm5.47\times10^{-7}$	$7.92\times10^{-7}\pm6.83\times10^{-7}$	$3.79\times10^{-7}\pm4.93\times10^{-7}$
DG 40:7|DG 18:1_22:6	$4.83\times10^{-4}\pm1.06\times10^{-4}$	$1.41\times10^{-4}\pm3.31\times10^{-5}$	$2.85\times10^{-4}\pm1.01\times10^{-4}$	$2.17\times10^{-4}\pm5.80\times10^{-5}$
DG 40:6|DG 18:2_22:4	$1.32\times10^{-5}\pm9.18\times10^{-6}$	$6.75\times10^{-6}\pm4.60\times10^{-6}$	$8.38\times10^{-6}\pm8.27\times10^{-6}$	$2.16\times10^{-6}\pm2.42\times10^{-6}$
DG 40:6|DG 16:0_24:6	$9.87\times10^{-5}\pm5.33\times10^{-5}$	$9.23\times10^{-5}\pm3.44\times10^{-5}$	$1.45\times10^{-4}\pm4.81\times10^{-5}$	$1.36\times10^{-4}\pm2.36\times10^{-5}$
DG 40:4|DG 18:0_22:4	$8.67\times10^{-5}\pm3.90\times10^{-5}$	$4.35\times10^{-5}\pm9.76\times10^{-6}$	$6.02\times10^{-5}\pm2.78\times10^{-5}$	$4.31\times10^{-5}\pm5.93\times10^{-6}$
DG 40:3|DG 22:1_18:2	$4.18\times10^{-5}\pm2.85\times10^{-5}$	$2.59\times10^{-5}\pm4.08\times10^{-6}$	$2.43\times10^{-5}\pm4.37\times10^{-6}$	$2.71\times10^{-5}\pm1.30\times10^{-5}$
DG 40:2|DG 22:0_18:2	$2.23\times10^{-5}\pm1.18\times10^{-5}$	$1.75\times10^{-5}\pm2.26\times10^{-6}$	$1.40\times10^{-5}\pm5.35\times10^{-6}$	$2.66\times10^{-5}\pm1.51\times10^{-5}$
DG 38:5|DG 18:1_20:4	$2.82\times10^{-4}\pm8.84\times10^{-5}$	$1.69\times10^{-4}\pm3.24\times10^{-5}$	$2.19\times10^{-4}\pm2.95\times10^{-5}$	$1.55\times10^{-4}\pm4.04\times10^{-5}$
DG 38:3|DG 18:0_20:3	$7.25\times10^{-4}\pm2.01\times10^{-4}$	$5.16\times10^{-4}\pm7.46\times10^{-5}$	$5.78\times10^{-4}\pm7.08\times10^{-5}$	$4.26\times10^{-4}\pm1.19\times10^{-4}$
DG 38:2|DG 20:0_18:2	$3.14\times10^{-4}\pm9.00\times10^{-5}$	$2.76\times10^{-4}\pm5.98\times10^{-5}$	$2.45\times10^{-4}\pm4.83\times10^{-5}$	$2.44\times10^{-4}\pm1.01\times10^{-4}$
DG 38:1|DG 20:0_18:1	$4.16\times10^{-5}\pm9.31\times10^{-6}$	$3.74\times10^{-5}\pm1.74\times10^{-5}$	$3.44\times10^{-5}\pm1.03\times10^{-5}$	$4.50\times10^{-5}\pm1.35\times10^{-5}$
DG 37:3|DG 19:1_18:2	$2.46\times10^{-5}\pm7.86\times10^{-6}$	$2.58\times10^{-5}\pm8.78\times10^{-6}$	$2.27\times10^{-5}\pm4.91\times10^{-6}$	$1.19\times10^{-5}\pm4.90\times10^{-6}$
DG 36:6|DG 18:2_18:4	$9.40\times10^{-7}\pm9.43\times10^{-7}$	$1.55\times10^{-6}\pm1.79\times10^{-6}$	$7.88\times10^{-7}\pm6.23\times10^{-7}$	$8.06\times10^{-8}\pm1.40\times10^{-7}$
DG 36:5|DG 18:2_18:3	$2.25\times10^{-6}\pm1.79\times10^{-6}$	$3.18\times10^{-6}\pm4.26\times10^{-6}$	$1.27\times10^{-6}\pm7.25\times10^{-7}$	$3.79\times10^{-7}\pm5.79\times10^{-7}$
DG 36:4|DG 18:2_18:2	$1.05\times10^{-4}\pm5.69\times10^{-5}$	$1.13\times10^{-4}\pm8.77\times10^{-5}$	$6.53\times10^{-5}\pm2.18\times10^{-5}$	$3.35\times10^{-5}\pm1.73\times10^{-5}$
DG 36:4|DG 16:0_20:4	$4.53\times10^{-5}\pm1.63\times10^{-5}$	$2.91\times10^{-5}\pm9.42\times10^{-6}$	$4.46\times10^{-5}\pm2.00\times10^{-5}$	$2.71\times10^{-5}\pm8.03\times10^{-6}$
DG 36:3|DG 18:1_18:2	$2.19\times10^{-4}\pm7.86\times10^{-5}$	$2.94\times10^{-4}\pm1.44\times10^{-4}$	$2.15\times10^{-4}\pm1.05\times10^{-4}$	$1.11\times10^{-4}\pm4.65\times10^{-5}$

DG 36:2|DG 18:0_18:2	$2.30\times10^{-3}\pm5.73\times10^{-4}$	$2.22\times10^{-3}\pm6.63\times10^{-4}$	$2.26\times10^{-3}\pm4.12\times10^{-4}$	$1.63\times10^{-3}\pm5.56\times10^{-4}$
DG 36:1|DG 18:0_18:1	$3.98\times10^{-4}\pm3.99\times10^{-5}$	$5.46\times10^{-4}\pm1.06\times10^{-4}$	$4.49\times10^{-4}\pm1.75\times10^{-4}$	$2.66\times10^{-4}\pm9.60\times10^{-5}$
DG 36:0|DG 18:0_18:0	$1.94\times10^{-4}\pm6.24\times10^{-5}$	$1.44\times10^{-4}\pm6.45\times10^{-5}$	$2.03\times10^{-4}\pm4.67\times10^{-5}$	$1.97\times10^{-4}\pm4.70\times10^{-5}$
DG 35:3|DG 17:1_18:2	$1.24\times10^{-6}\pm9.71\times10^{-7}$	$1.04\times10^{-6}\pm1.32\times10^{-6}$	$6.00\times10^{-7}\pm3.46\times10^{-7}$	$2.16\times10^{-7}\pm2.67\times10^{-7}$
DG 35:2|DG 17:0_18:2	$2.87\times10^{-5}\pm7.03\times10^{-6}$	$2.86\times10^{-5}\pm9.39\times10^{-6}$	$3.15\times10^{-5}\pm1.07\times10^{-5}$	$2.04\times10^{-5}\pm9.45\times10^{-6}$
DG 34:3|DG 16:1_18:2	$1.32\times10^{-5}\pm1.13\times10^{-5}$	$1.85\times10^{-5}\pm1.68\times10^{-5}$	$8.99\times10^{-6}\pm2.34\times10^{-6}$	$2.25\times10^{-6}\pm9.63\times10^{-7}$
DG 34:3|DG 16:0_18:3	$5.21\times10^{-6}\pm4.44\times10^{-6}$	$7.02\times10^{-6}\pm7.17\times10^{-6}$	$3.28\times10^{-6}\pm2.57\times10^{-6}$	$1.80\times10^{-6}\pm1.67\times10^{-6}$
DG 34:2|DG 16:0_18:2	$2.17\times10^{-4}\pm8.83\times10^{-5}$	$1.92\times10^{-4}\pm8.55\times10^{-5}$	$1.90\times10^{-4}\pm4.38\times10^{-5}$	$1.12\times10^{-4}\pm3.88\times10^{-5}$
DG 34:1|DG 16:0_18:1	$6.58\times10^{-4}\pm9.01\times10^{-5}$	$5.83\times10^{-4}\pm1.96\times10^{-4}$	$6.81\times10^{-4}\pm8.12\times10^{-5}$	$5.07\times10^{-4}\pm1.55\times10^{-4}$
DG 34:0|DG 16:0_18:0	$1.19\times10^{-4}\pm4.28\times10^{-5}$	$9.99\times10^{-5}\pm2.04\times10^{-5}$	$1.30\times10^{-4}\pm2.16\times10^{-5}$	$1.35\times10^{-4}\pm2.16\times10^{-5}$
DG 32:2|DG 14:0_18:2	$1.44\times10^{-6}\pm7.70\times10^{-7}$	$4.97\times10^{-6}\pm3.34\times10^{-6}$	$2.49\times10^{-6}\pm1.22\times10^{-6}$	$1.51\times10^{-6}\pm1.03\times10^{-6}$
DG 32:0|DG 16:0_16:0	$2.53\times10^{-5}\pm6.53\times10^{-6}$	$2.27\times10^{-5}\pm5.67\times10^{-6}$	$3.20\times10^{-5}\pm2.32\times10^{-6}$	$3.75\times10^{-5}\pm6.00\times10^{-6}$
CE 22:6	$8.74\times10^{-5}\pm1.42\times10^{-5}$	$4.76\times10^{-5}\pm3.95\times10^{-5}$	$8.63\times10^{-5}\pm4.74\times10^{-5}$	$6.52\times10^{-5}\pm1.06\times10^{-5}$
CE 18:1	$5.33\times10^{-5}\pm6.05\times10^{-5}$	$1.06\times10^{-5}\pm1.18\times10^{-5}$	$2.74\times10^{-5}\pm2.35\times10^{-5}$	$6.59\times10^{-6}\pm2.11\times10^{-6}$
CAR 24:2	$8.71\times10^{-6}\pm3.31\times10^{-6}$	$5.62\times10^{-6}\pm1.19\times10^{-6}$	$5.50\times10^{-6}\pm2.21\times10^{-6}$	$7.08\times10^{-6}\pm2.12\times10^{-6}$
CAR 24:1	$3.11\times10^{-5}\pm1.71\times10^{-5}$	$1.68\times10^{-5}\pm5.41\times10^{-6}$	$1.97\times10^{-5}\pm7.80\times10^{-6}$	$2.68\times10^{-5}\pm1.10\times10^{-5}$
CAR 24:0	$1.41\times10^{-5}\pm8.48\times10^{-6}$	$1.26\times10^{-5}\pm6.04\times10^{-6}$	$1.05\times10^{-5}\pm3.67\times10^{-6}$	$1.40\times10^{-5}\pm3.89\times10^{-6}$
CAR 23:4	$5.73\times10^{-5}\pm7.28\times10^{-6}$	$3.00\times10^{-5}\pm4.80\times10^{-6}$	$3.19\times10^{-5}\pm2.70\times10^{-5}$	$2.29\times10^{-5}\pm9.37\times10^{-6}$
CAR 23:3	$3.21\times10^{-5}\pm3.25\times10^{-6}$	$2.26\times10^{-5}\pm4.14\times10^{-6}$	$2.17\times10^{-5}\pm1.11\times10^{-5}$	$1.94\times10^{-5}\pm5.80\times10^{-6}$
CAR 23:2	$1.40\times10^{-5}\pm7.84\times10^{-6}$	$9.74\times10^{-6}\pm6.02\times10^{-6}$	$1.09\times10^{-5}\pm4.30\times10^{-6}$	$1.16\times10^{-5}\pm4.87\times10^{-6}$
CAR 23:1	$2.81\times10^{-5}\pm1.52\times10^{-5}$	$1.82\times10^{-5}\pm6.95\times10^{-6}$	$3.11\times10^{-5}\pm1.82\times10^{-5}$	$2.26\times10^{-5}\pm8.77\times10^{-6}$
CAR 22:6	$5.26\times10^{-5}\pm1.17\times10^{-5}$	$3.34\times10^{-5}\pm6.79\times10^{-6}$	$3.43\times10^{-5}\pm1.50\times10^{-5}$	$3.02\times10^{-5}\pm8.90\times10^{-6}$
CAR 22:1	$5.66\times10^{-5}\pm3.10\times10^{-5}$	$3.21\times10^{-5}\pm3.81\times10^{-6}$	$3.48\times10^{-5}\pm1.37\times10^{-5}$	$3.99\times10^{-5}\pm8.13\times10^{-6}$
CAR 22:0	$6.40\times10^{-5}\pm2.92\times10^{-5}$	$3.60\times10^{-5}\pm1.02\times10^{-5}$	$2.97\times10^{-5}\pm7.74\times10^{-6}$	$6.21\times10^{-5}\pm1.63\times10^{-5}$
CAR 21:1	$1.62\times10^{-4}\pm5.01\times10^{-5}$	$9.64\times10^{-5}\pm5.78\times10^{-5}$	$1.35\times10^{-4}\pm8.37\times10^{-5}$	$1.04\times10^{-4}\pm4.49\times10^{-5}$
CAR 21:0	$1.30\times10^{-4}\pm4.84\times10^{-5}$	$7.66\times10^{-5}\pm1.78\times10^{-5}$	$7.78\times10^{-5}\pm4.11\times10^{-5}$	$9.57\times10^{-5}\pm2.46\times10^{-5}$

CAR 20:4	$1.30\times10^{-4}\pm2.05\times10^{-5}$	$7.67\times10^{-5}\pm1.08\times10^{-5}$	$7.89\times10^{-5}\pm2.02\times10^{-5}$	$5.83\times10^{-5}\pm1.66\times10^{-5}$
CAR 20:0	$4.43\times10^{-5}\pm1.97\times10^{-5}$	$2.50\times10^{-5}\pm4.16\times10^{-6}$	$2.37\times10^{-5}\pm1.02\times10^{-5}$	$2.93\times10^{-5}\pm2.37\times10^{-6}$
CAR 19:0	$8.48\times10^{-5}\pm1.22\times10^{-5}$	$5.88\times10^{-5}\pm2.81\times10^{-5}$	$5.87\times10^{-5}\pm2.96\times10^{-5}$	$4.89\times10^{-5}\pm9.74\times10^{-6}$
CAR 18:2	$1.20\times10^{-4}\pm1.40\times10^{-5}$	$6.84\times10^{-5}\pm6.75\times10^{-6}$	$8.59\times10^{-5}\pm2.48\times10^{-5}$	$6.43\times10^{-5}\pm2.83\times10^{-5}$
CAR 18:1	$1.92\times10^{-4}\pm5.52\times10^{-5}$	$9.58\times10^{-5}\pm4.64\times10^{-5}$	$1.41\times10^{-4}\pm6.37\times10^{-5}$	$8.56\times10^{-5}\pm6.32\times10^{-5}$
CAR 18:0	$2.71\times10^{-4}\pm3.78\times10^{-5}$	$1.78\times10^{-4}\pm1.36\times10^{-5}$	$2.05\times10^{-4}\pm9.17\times10^{-5}$	$1.62\times10^{-4}\pm3.34\times10^{-5}$

"-" indicates not detected.

Table S4. VIP score plots of PLS-DA showed a total of 113 lipids for VIP>1

Name	VIP
TG 72:2|TG 36:0_18:1_18:1	1.0339
TG 70:3|TG 18:1_18:1_34:1	1.3869
TG 70:2|TG 18:0_18:1_34:1	1.1329
TG 70:2|TG 16:0_18:1_36:1	1.0588
TG 64:3|TG 18:1_18:1_28:1	2.4883
TG 64:2|TG 28:0_18:1_18:1	1.0543
TG 60:3|TG 20:1_20:1_20:1	3.6512
TG 60:3|TG 18:1_18:1_24:1	1.7778
TG 58:2|TG 16:0_20:1_22:1	1.3961
TG 58:2|TG 16:0_18:1_24:1	2.7396
TG 57:2|TG 18:0_21:0_18:2	2.9003
TG 56:2|TG 20:0_18:1_18:1	1.1087
TG 56:1|TG 16:0_22:0_18:1	1.1847

TG 55:5|TG 19:1_18:2_18:2	1.3837
TG 55:2|TG 19:0_18:1_18:1	2.016
TG 55:2|TG 17:0_18:1_20:1	2.2569
TG 55:1|TG 18:0_19:0_18:1	2.9634
TG 54:7|TG 18:2_18:2_18:3	1.3723
TG 54:6|TG 18:2_18:2_18:2	1.51
TG 54:4|TG 18:1_18:1_18:2	1.2334
TG 54:3|TG 18:0_18:1_18:2	1.3644
TG 54:2|TG 18:0_18:1_18:1	1.2008
TG 53:5|TG 17:1_18:2_18:2	2.1231
TG 53:4|TG 17:0_18:2_18:2	1.8564
TG 53:2|TG 17:0_18:1_18:1	2.0177
TG 53:2|TG 16:0_18:1_19:1	1.2593
TG 53:1|TG 16:0_19:0_18:1	2.0121
TG 52:6|TG 16:1_18:2_18:3	1.722
TG 52:5|TG 16:0_16:0_20:5	3.029
TG 52:4|TG 16:0_18:1_18:3	1.2524
TG 52:1|TG 16:0_18:0_18:1	1.2062
TG 51:4|TG 16:1_17:1_18:2	3.5623
TG 51:4|TG 15:0_18:2_18:2	2.7603
TG 51:2|TG 15:0_18:1_18:1	1.8027
TG 51:1|TG 16:0_17:0_18:1	2.3498
TG 51:0|TG 17:0_17:0_17:0	2.4218
TG 51:0|TG 16:0_17:0_18:0	3.4764
TG 51:0|TG 16:0_16:0_19:0	2.5582
TG 50:5|TG 16:1_16:1_18:3	3.9516

TG 50:4|TG 16:0_16:1_18:3	2.4707
TG 50:4|TG 14:0_18:2_18:2	2.0437
TG 50:3|TG 16:0_16:0_18:3	1.6382
TG 50:1|TG 16:0_16:0_18:1	1.4872
TG 49:3|TG 15:0_16:1_18:2	2.4854
TG 49:1|TG 16:0_16:0_17:1	2.0047
TG 49:0|TG 16:0_16:0_17:0	1.9079
TG 48:3|TG 16:1_16:1_16:1	2.4236
TG 48:3|TG 14:0_16:1_18:2	2.4862
TG 48:0|TG 16:0_16:0_16:0	2.3214
TG 47:2|TG 14:0_15:0_18:2	7.0317
TG 46:3|TG 14:1_16:1_16:1	3.5928
TG 46:3|TG 14:0_14:0_18:3	2.5869
TG 46:2|TG 14:0_14:0_18:2	2.6421
TG 44:1|TG 14:0_14:0_16:1	1.2288
TG 40:0|TG 8:0_16:0_16:0	3.2633
TG 36:0|TG 12:0_12:0_12:0	1.9456
SM 58:3;2O	1.5853
SM 52:9;2O	1.584
PMeOH 38:6|PMeOH 16:0_22:6	1.7057
PI 40:4|PI 20:1_20:3	1.4632
PI 40:4|PI 20:0_20:4	2.024
PI 40:3|PI 20:0_20:3	2.7849
PI 40:3|PI 18:0_22:3	1.5916
PI 38:5|PI 18:1_20:4	1.2927
PI 38:5;O|PI 18:1_20:4;O	1.0806

PI 38:4;3O|PI 18:0_20:4;3O	1.1477
PI 38:3|PI 18:0_20:3	5.0262
PI 37:4|PI 17:0_20:4	1.03
PI 36:3;O|PI 18:1_18:2;O	1.5804
PI 36:1|PI 18:0_18:1	1.2425
PI 34:2|PI 16:0_18:2	1.4004
PI 34:2;O|PI 16:0_18:2;O	1.0598
PI 34:1|PI 16:0_18:1	1.6464
PI 34:0|PI 16:0_18:0	3.4023
PG O-36:3|PG O-18:2_18:1	1.6404
PG O-34:2|PG O-16:1_18:1	1.5244
PG 38:6|PG 16:0_22:6	1.0563
PG 38:2|PG 18:0_20:2	2.9117
PG 36:4|PG 16:0_20:4	1.2008
PG 36:1|PG 18:0_18:1	1.134
PG 35:2|PG 17:0_18:2	1.5154
PG 35:1|PG 17:0_18:1	2.2877
PG 34:2|PG 16:0_18:2	3.1898
PG 34:1|PG 16:0_18:1	1.2243
PE O-36:5|PE O-16:1_20:4	1.6561
PE O-36:4|PE O-16:1_20:3	2.4258
PE O-34:3|PE O-16:1_18:2	1.3877
PE 42:6|PE 20:0_22:6	2.0721
PE 40:6|PE 18:0_22:6	1.2156
PE 40:5|PE 18:0_22:5	1.3099
PE 39:6|PE 17:0_22:6	1.222

PE 39:4|PE 19:0_20:4	1.0488
PE 38:7|PE 18:2_20:5	1.2114
PE 38:5|PE 18:1_20:4	1.1814
PE 38:5;2O|PE 18:1_20:4;2O	1.0636
PE 36:5|PE 18:2_18:3	4.3858
PE 36:3|PE 16:0_20:3	1.0132
PE 34:2|PE 16:0_18:2	1.0294
PE 32:0|PE 16:0_16:0	2.1283
PC O-35:2	2.4944
PC 44:4	1.9101
PC 39:1	1.4355
PC 34:2;2O|PC 16:0_18:2;2O	1.1382
PA 34:2|PA 16:0_18:2	2.2072
PA 32:0|PA 16:0_16:0	2.271
LPE 18:1	1.6124
FAHFA 18:0|FAHFA 16:0/2:0	1.1539
FA 20:0	2.9577
Dodecylbenzenesulfonic acid	1.0696
DG 36:6|DG 18:2_18:4	2.7172
DG 36:5|DG 18:2_18:3	1.0057
DG 34:3|DG 16:1_18:2	1.136
DG 34:3|DG 16:0_18:3	1.9369

Table S5. ANOVA analysis lipids were found to be significantly different between HFD, HAD, HBD, and HGD groups

Name	f.value	p.value	FDR
PG 34:2|PG 16:0_18:2	55.037	1.189e-08	6.8204e-06
PG 36:1|PG 18:0_18:1	49.212	2.6652e-08	6.8204e-06
PG 34:1|PG 16:0_18:1	47.491	3.4389e-08	6.8204e-06
PI 34:2|PI 16:0_18:2	41.595	8.8085e-08	1.3103e-05
PI 40:4|PI 20:1_20:3	35.178	2.8307e-07	3.3685e-05
PI 38:3|PI 18:0_20:3	33.144	4.2593e-07	4.2238e-05
PE 35:1|PE 17:0_18:1	31.159	6.4824e-07	5.0398e-05
PI 40:4|PI 20:0_20:4	30.955	6.7762e-07	5.0398e-05
PE 38:5|PE 18:1_20:4	29.269	9.8779e-07	6.5304e-05
PE 34:2|PE 16:0_18:2	28.326	1.2295e-06	7.3154e-05
PG O-34:2|PG O-16:1_18:1	27.368	1.5453e-06	8.3588e-05
PE 32:1|PE 16:0_16:1	26.571	1.8786e-06	9.3149e-05
PI 38:4|PI 18:0_20:4	24.474	3.2159e-06	0.00014719
PG O-36:3|PG O-18:2_18:1	23.417	4.2775e-06	0.00018179
PE 40:5|PE 18:0_22:5	22.676	5.2575e-06	0.00020855
PE 39:4|PE 19:0_20:4	22.309	5.8356e-06	0.00021701
PE 36:3|PE 16:0_20:3	20.435	1.0153e-05	0.00035537
PE 36:5|PE 18:2_18:3	16.853	3.3012e-05	0.0010912
PE 36:4|PE 16:0_20:4	15.654	5.1079e-05	0.0015996
PE 34:1|PE 16:0_18:1	15.259	5.9298e-05	0.0017641
PE 37:2|PE 19:0_18:2	14.626	7.5749e-05	0.0021339
LPE 18:3	14.523	7.8899e-05	0.0021339
PI 36:3|PI 16:0_20:3	14.211	8.9311e-05	0.0023104

PG 35:2|PG 17:0_18:2	13.878	0.00010222	0.0025343
PE 36:5|PE 16:1_20:4	13.379	0.00012561	0.0029894
PG 38:3|PG 20:1_18:2	12.953	0.0001504	0.0033857
PI 38:5|PI 16:0_22:5	12.852	0.00015705	0.0033857
PC 39:3	12.819	0.00015933	0.0033857
PE 34:3|PE 16:0_18:3	12.273	0.00020231	0.0039249
PI 40:3|PI 20:0_20:3	12.268	0.00020272	0.0039249
PI 34:0|PI 16:0_18:0	12.249	0.00020449	0.0039249
PC 44:4	11.846	0.0002451	0.0045574
PE O-36:3|PE O-18:2_18:1	11.495	0.00028795	0.0051918
PI 40:6|PI 18:0_22:6	11.243	0.00032382	0.0056669
PI 38:6|PI 18:2_20:4	11.02	0.00035988	0.0061179
PI 42:10|PI 20:4_22:6	10.945	0.00037312	0.0061668
PG 35:1|PG 17:0_18:1	10.642	0.00043192	0.0066312
PC O-38:5	10.634	0.00043348	0.0066312
DG 40:7|DG 18:1_22:6	10.614	0.00043775	0.0066312
PE O-38:6|PE O-16:1_22:5	10.577	0.00044579	0.0066312
PA 34:2|PA 16:0_18:2	10.404	0.00048537	0.0070437
PE 42:6|PE 20:0_22:6	10.129	0.00055669	0.0078864
FA 22:4	9.9368	0.00061369	0.0083612
PE O-42:6|PE O-20:0_22:6	9.9221	0.0006183	0.0083612
Dodecylbenzenesulfonic acid	9.6255	0.0007202	0.0095226
PI 39:3|PI 19:0_20:3	9.5731	0.00074008	0.0095323
TG 49:1|TG 16:0_16:0_17:1	9.5251	0.00075886	0.0095323
PC 44:12|PC 22:6_22:6	9.4455	0.00079119	0.0095323
PI 36:4|PI 16:0_20:4	9.4141	0.00080434	0.0095323

PE 38:4|PE 18:1_20:3	9.3544	0.00083006	0.0095323
PG 38:2|PG 18:0_20:2	9.3306	0.00084061	0.0095323
LPC 23:0/0:0	9.3268	0.00084229	0.0095323
SM 58:3;2O	9.285	0.00086114	0.0095323
CAR 20:4	9.2764	0.00086512	0.0095323
PI 36:3|PI 18:1_18:2	9.07	0.00096605	0.010296
PI 36:2|PI 18:1_18:1	9.0642	0.00096903	0.010296
TG 53:2|TG 17:0_18:1_18:1	8.9696	0.0010199	0.010639
LPC 24:5/0:0	8.9387	0.0010371	0.010639
LPC O-20:2	8.8059	0.001115	0.011245
TG 49:3|TG 15:0_16:1_18:2	8.7255	0.0011654	0.011557
PI 40:4|PI 18:0_22:4	8.6363	0.0012242	0.011941
PI 37:4|PI 17:0_20:4	8.5903	0.0012559	0.012052
PC O-44:12	8.0855	0.0016711	0.015707
LPE 20:2	8.0477	0.0017079	0.015707
PE 39:6|PE 17:0_22:6	8.0396	0.0017158	0.015707
DG 32:0|DG 16:0_16:0	7.9888	0.001767	0.015929
PI 38:6|PI 16:0_22:6	7.9059	0.0018543	0.016467
TG 54:2|TG 18:0_18:1_18:1	7.8469	0.0019193	0.016794
PE O-36:4|PE O-16:1_20:3	7.689	0.0021064	0.018164
TG 50:3|TG 16:0_16:0_18:3	7.5967	0.0022251	0.018913
PI 34:1	7.3013	0.0026586	0.02228
SM 34:2;2O	7.2706	0.0027089	0.022386
LPC 19:0	7.2259	0.002784	0.02251
PI 36:2	7.2168	0.0027996	0.02251
PE O-40:6|PE O-18:0_22:6	7.1876	0.0028502	0.022612

PI 40:5|PI 18:0_22:5	7.1411	0.002933	0.022731
LPC O-18:2	7.1363	0.0029416	0.022731
PE 40:8|PE 18:2_22:6	7.0958	0.0030161	0.023008
PI 36:0	7.0453	0.0031122	0.02344
PA 32:0|PA 16:0_16:0	7.0242	0.0031533	0.023452
PE 42:8|PE 20:2_22:6	6.7554	0.0037335	0.027174
PMeOH 38:6|PMeOH 16:0_22:6	6.7506	0.003745	0.027174
PI 38:5|PI 18:2_20:3	6.7095	0.0038441	0.027557
PC 35:1	6.643	0.004011	0.028159
TG 52:4|TG 16:0_18:1_18:3	6.6384	0.0040227	0.028159
PE 38:6|PE 16:0_22:6	6.6164	0.0040798	0.028227
TG 36:0|TG 12:0_12:0_12:0	6.5931	0.0041415	0.028324
PI 38:2|PI 18:0_20:2	6.5202	0.0043408	0.029057
PG 36:4|PG 16:0_20:4	6.4993	0.0043999	0.029057
PE 42:8|PE 20:3_22:5	6.4881	0.0044318	0.029057
SM 40:1;2O|SM 18:1;2O/22:0	6.4839	0.004444	0.029057
FA 20:2	6.4145	0.0046492	0.030068
PC O-40:5	6.3753	0.0047695	0.030515
LPC 18:1/0:0	6.332	0.004907	0.03106
LPC O-24:1	6.2069	0.005329	0.033376
PI 40:3|PI 18:0_22:3	6.1686	0.005466	0.033878
LPE O-18:1	6.0956	0.0057389	0.034606
PI 40:6|PI 20:2_20:4	6.0867	0.0057729	0.034606
FA 20:0	6.0817	0.0057924	0.034606
PE 36:5|PE 16:0_20:5	6.0756	0.0058161	0.034606
TG 56:6|TG 16:0_18:1_22:5	6.0219	0.0060295	0.03552

PC 37:1	6.0029	0.0061074	0.035626
TG 55:2|TG 19:0_18:1_18:1	5.9765	0.0062168	0.035912
LPE 17:0	5.8315	0.0068603	0.039249
PC 42:10	5.6573	0.0077339	0.043801
TG 55:2|TG 17:0_18:1_20:1	5.633	0.0078655	0.043801
TG 53:4|TG 17:0_18:2_18:2	5.6139	0.0079708	0.043801
PE O-34:3|PE O-16:1_18:2	5.5983	0.0080578	0.043801
TG 64:3|TG 18:1_18:1_28:1	5.5939	0.0080824	0.043801
PE 38:3|PE 18:0_20:3	5.5912	0.0080976	0.043801
PC O-42:9	5.5239	0.0084876	0.045496
LPC O-18:1	5.4601	0.0088766	0.047157
PI 40:7|PI 18:1_22:6	5.3991	0.0092679	0.0488
TG 51:2|TG 15:0_18:1_18:1	5.3813	0.0093857	0.048987

Table S6. Concentrations of metabolites in mice cecum content from CON, HFD, HAD, HBD and HGD groups

Name	CON	HFD	HAD	HBD	HGD
Formate	$1.13\times10^{-4}\pm2.89\times10^{-5}$	$1.30\times10^{-4}\pm9.27\times10^{-5}$	$1.23\times10^{-4}\pm3.76\times10^{-5}$	$3.35\times10^{-4}\pm4.53\times10^{-4}$	$1.73\times10^{-4}\pm3.88\times10^{-5}$
Inosine	$2.11\times10^{-5}\pm3.63\times10^{-6}$	$8.91\times10^{-5}\pm1.29\times10^{-4}$	$5.10\times10^{-5}\pm4.42\times10^{-5}$	$4.68\times10^{-5}\pm3.27\times10^{-5}$	$2.32\times10^{-5}\pm1.20\times10^{-5}$
2'-Deoxyinosine	$3.44\times10^{-5}\pm2.76\times10^{-5}$	$3.04\times10^{-5}\pm1.41\times10^{-5}$	$6.55\times10^{-5}\pm2.81\times10^{-5}$	$5.34\times10^{-5}\pm4.24\times10^{-5}$	$2.44\times10^{-5}\pm1.16\times10^{-5}$
Oxypurinol	$2.65\times10^{-5}\pm2.61\times10^{-5}$	$2.31\times10^{-5}\pm1.35\times10^{-5}$	$5.37\times10^{-5}\pm2.95\times10^{-5}$	$4.83\times10^{-5}\pm4.60\times10^{-5}$	$1.76\times10^{-5}\pm8.28\times10^{-6}$

Hypoxanthine	$2.39\times10^{-4}\pm1.58\times10^{-4}$	$1.50\times10^{-4}\pm1.17\times10^{-4}$	$1.51\times10^{-4}\pm1.25\times10^{-4}$	$2.14\times10^{-4}\pm1.06\times10^{-4}$	$2.33\times10^{-4}\pm1.29\times10^{-4}$
Xanthine	$3.87\times10^{-4}\pm2.63\times10^{-4}$	$2.28\times10^{-4}\pm1.98\times10^{-4}$	$1.93\times10^{-4}\pm1.94\times10^{-4}$	$3.33\times10^{-4}\pm1.77\times10^{-4}$	$3.35\times10^{-4}\pm2.33\times10^{-4}$
Tryptophan	$6.02\times10^{-4}\pm3.37\times10^{-4}$	$7.14\times10^{-4}\pm3.94\times10^{-4}$	$7.74\times10^{-4}\pm2.61\times10^{-4}$	$5.14\times10^{-4}\pm1.74\times10^{-4}$	$5.97\times10^{-4}\pm3.01\times10^{-4}$
Thymidine	$5.86\times10^{-5}\pm4.52\times10^{-5}$	$4.83\times10^{-5}\pm1.57\times10^{-5}$	$7.02\times10^{-5}\pm1.98\times10^{-5}$	$5.27\times10^{-5}\pm3.12\times10^{-5}$	$3.37\times10^{-5}\pm1.61\times10^{-5}$
Phenylalanine	$2.26\times10^{-3}\pm9.85\times10^{-4}$	$3.03\times10^{-3}\pm2.32\times10^{-3}$	$3.02\times10^{-3}\pm1.14\times10^{-3}$	$1.97\times10^{-3}\pm4.62\times10^{-4}$	$3.09\times10^{-3}\pm2.00\times10^{-3}$
Phenylacetate	$1.28\times10^{-4}\pm1.03\times10^{-4}$	$7.38\times10^{-5}\pm3.32\times10^{-5}$	$6.35\times10^{-5}\pm3.43\times10^{-5}$	$5.48\times10^{-5}\pm2.48\times10^{-5}$	$6.87\times10^{-5}\pm3.09\times10^{-5}$
Tyrosine	$1.67\times10^{-3}\pm7.58\times10^{-4}$	$2.51\times10^{-3}\pm1.95\times10^{-3}$	$2.34\times10^{-3}\pm8.99\times10^{-4}$	$1.57\times10^{-3}\pm4.56\times10^{-4}$	$2.54\times10^{-3}\pm1.65\times10^{-3}$
4-Hydroxyphenylacetate	$9.91\times10^{-5}\pm7.44\times10^{-5}$	$8.55\times10^{-5}\pm6.24\times10^{-5}$	$5.15\times10^{-5}\pm2.44\times10^{-5}$	$4.97\times10^{-5}\pm1.94\times10^{-5}$	$5.69\times10^{-5}\pm1.86\times10^{-5}$
p-Methylhistidine	$4.09\times10^{-4}\pm3.50\times10^{-4}$	$7.43\times10^{-4}\pm6.64\times10^{-4}$	$5.87\times10^{-4}\pm2.27\times10^{-4}$	$4.06\times10^{-4}\pm2.63\times10^{-4}$	$5.10\times10^{-4}\pm5.17\times10^{-4}$
Ferulate	$4.45\times10^{-5}\pm1.24\times10^{-5}$	$8.96\times10^{-5}\pm7.10\times10^{-5}$	$3.20\times10^{-5}\pm1.87\times10^{-5}$	$7.33\times10^{-5}\pm3.19\times10^{-5}$	$2.72\times10^{-5}\pm1.45\times10^{-5}$
Fumarate	$1.66\times10^{-4}\pm1.14\times10^{-4}$	$1.44\times10^{-4}\pm8.86\times10^{-5}$	$8.10\times10^{-5}\pm5.33\times10^{-5}$	$1.23\times10^{-4}\pm4.87\times10^{-5}$	$1.84\times10^{-4}\pm1.31\times10^{-4}$
2'-Deoxyuridine	$6.57\times10^{-5}\pm4.99\times10^{-5}$	$4.97\times10^{-5}\pm1.19\times10^{-5}$	$8.64\times10^{-5}\pm3.58\times10^{-5}$	$8.06\times10^{-5}\pm5.41\times10^{-5}$	$3.37\times10^{-5}\pm1.53\times10^{-5}$
Cytosine	$4.69\times10^{-5}\pm8.68\times10^{-6}$	$4.07\times10^{-5}\pm2.17\times10^{-5}$	$3.40\times10^{-5}\pm8.52\times10^{-6}$	$6.94\times10^{-5}\pm2.71\times10^{-5}$	$3.25\times10^{-5}\pm2.03\times10^{-5}$
Uridine	$5.69\times10^{-5}\pm4.45\times10^{-5}$	$6.54\times10^{-5}\pm3.18\times10^{-5}$	$9.51\times10^{-5}\pm5.74\times10^{-5}$	$6.51\times10^{-5}\pm7.58\times10^{-5}$	$5.19\times10^{-5}\pm4.36\times10^{-5}$
Uracil	$7.01\times10^{-4}\pm3.23\times10^{-4}$	$9.44\times10^{-4}\pm4.45\times10^{-4}$	$5.33\times10^{-4}\pm1.73\times10^{-4}$	$6.42\times10^{-4}\pm2.31\times10^{-4}$	$9.56\times10^{-4}\pm4.31\times10^{-4}$
Galactose	$2.08\times10^{-3}\pm1.02\times10^{-3}$	$2.01\times10^{-3}\pm1.57\times10^{-3}$	$1.45\times10^{-3}\pm1.19\times10^{-3}$	$1.98\times10^{-3}\pm7.33\times10^{-4}$	$1.27\times10^{-3}\pm6.74\times10^{-4}$
Xylose	$2.71\times10^{-3}\pm1.50\times10^{-3}$	$3.04\times10^{-3}\pm2.85\times10^{-3}$	$1.89\times10^{-3}\pm1.82\times10^{-3}$	$2.34\times10^{-3}\pm1.46\times10^{-3}$	$2.61\times10^{-3}\pm1.60\times10^{-3}$
Mannose	$6.78\times10^{-5}\pm4.38\times10^{-5}$	$9.78\times10^{-5}\pm2.24\times10^{-5}$	$5.00\times10^{-5}\pm3.80\times10^{-5}$	$1.10\times10^{-4}\pm3.12\times10^{-5}$	$1.69\times10^{-4}\pm8.41\times10^{-5}$
Threonine	$1.59\times10^{-3}\pm5.98\times10^{-4}$	$2.53\times10^{-3}\pm2.07\times10^{-3}$	$2.01\times10^{-3}\pm5.98\times10^{-4}$	$1.72\times10^{-3}\pm3.44\times10^{-4}$	$2.51\times10^{-3}\pm1.66\times10^{-3}$
Serine	$2.03\times10^{-3}\pm1.10\times10^{-3}$	$2.89\times10^{-3}\pm2.53\times10^{-3}$	$2.78\times10^{-3}\pm9.91\times10^{-4}$	$2.24\times10^{-3}\pm7.20\times10^{-4}$	$2.86\times10^{-3}\pm1.76\times10^{-3}$
Guanidoacetate	$6.79\times10^{-4}\pm6.49\times10^{-5}$	$7.80\times10^{-4}\pm4.38\times10^{-4}$	$6.78\times10^{-4}\pm7.75\times10^{-5}$	$5.82\times10^{-4}\pm5.33\times10^{-5}$	$7.06\times10^{-4}\pm2.83\times10^{-4}$
Glycerol	$1.66\times10^{-3}\pm7.53\times10^{-4}$	$3.16\times10^{-3}\pm1.65\times10^{-3}$	$3.02\times10^{-3}\pm1.43\times10^{-3}$	$3.28\times10^{-3}\pm1.72\times10^{-3}$	$4.28\times10^{-3}\pm1.10\times10^{-3}$
Fructose	$2.01\times10^{-3}\pm2.72\times10^{-3}$	$6.28\times10^{-4}\pm4.33\times10^{-4}$	$1.24\times10^{-3}\pm6.14\times10^{-4}$	$8.15\times10^{-4}\pm8.24\times10^{-4}$	$1.08\times10^{-3}\pm1.37\times10^{-3}$
Glycine	$3.46\times10^{-3}\pm1.30\times10^{-3}$	$4.68\times10^{-3}\pm3.60\times10^{-3}$	$3.85\times10^{-3}\pm1.23\times10^{-3}$	$3.65\times10^{-3}\pm1.29\times10^{-3}$	$4.66\times10^{-3}\pm2.68\times10^{-3}$
Glucose	$5.08\times10^{-3}\pm8.77\times10^{-3}$	$3.69\times10^{-3}\pm2.13\times10^{-3}$	$1.24\times10^{-2}\pm8.01\times10^{-3}$	$2.19\times10^{-3}\pm7.15\times10^{-4}$	$7.73\times10^{-3}\pm3.58\times10^{-3}$
Methanol	$6.65\times10^{-4}\pm9.76\times10^{-5}$	$5.60\times10^{-4}\pm2.17\times10^{-4}$	$5.02\times10^{-4}\pm1.28\times10^{-4}$	$5.21\times10^{-4}\pm1.81\times10^{-4}$	$4.86\times10^{-4}\pm1.53\times10^{-4}$

Proline	$2.52\times10^{-3}\pm7.54\times10^{-4}$	$3.35\times10^{-3}\pm2.26\times10^{-3}$	$3.35\times10^{-3}\pm9.36\times10^{-4}$	$2.33\times10^{-3}\pm5.90\times10^{-4}$	$3.35\times10^{-3}\pm1.88\times10^{-3}$
Taurine	$6.33\times10^{-3}\pm4.27\times10^{-3}$	$2.66\times10^{-3}\pm9.21\times10^{-4}$	$2.69\times10^{-3}\pm1.26\times10^{-3}$	$3.75\times10^{-3}\pm1.21\times10^{-3}$	$2.70\times10^{-3}\pm1.39\times10^{-3}$
O-Acetylcholine	$2.56\times10^{-5}\pm1.92\times10^{-5}$	$7.14\times10^{-5}\pm8.02\times10^{-5}$	$1.61\times10^{-4}\pm1.47\times10^{-4}$	$2.36\times10^{-5}\pm5.52\times10^{-6}$	$6.08\times10^{-5}\pm7.68\times10^{-5}$
Choline	$6.39\times10^{-4}\pm3.37\times10^{-4}$	$1.72\times10^{-3}\pm1.00\times10^{-3}$	$1.23\times10^{-3}\pm3.59\times10^{-4}$	$1.20\times10^{-3}\pm3.15\times10^{-4}$	$1.28\times10^{-3}\pm5.91\times10^{-4}$
Dimethyl sulfone	$3.67\times10^{-5}\pm1.18\times10^{-5}$	$4.78\times10^{-5}\pm3.04\times10^{-5}$	$3.08\times10^{-5}\pm3.86\times10^{-6}$	$3.91\times10^{-5}\pm1.16\times10^{-5}$	$4.24\times10^{-5}\pm1.35\times10^{-5}$
Malonate	$1.53\times10^{-4}\pm3.97\times10^{-5}$	$1.91\times10^{-4}\pm1.20\times10^{-4}$	$1.44\times10^{-4}\pm2.54\times10^{-5}$	$1.44\times10^{-4}\pm3.52\times10^{-5}$	$2.06\times10^{-4}\pm8.30\times10^{-5}$
Creatine	$4.29\times10^{-4}\pm1.82\times10^{-4}$	$6.64\times10^{-4}\pm4.04\times10^{-4}$	$7.02\times10^{-4}\pm3.23\times10^{-4}$	$4.85\times10^{-4}\pm6.99\times10^{-5}$	$6.80\times10^{-4}\pm4.82\times10^{-4}$
Lysine	$3.02\times10^{-3}\pm8.31\times10^{-4}$	$4.29\times10^{-3}\pm2.79\times10^{-3}$	$3.63\times10^{-3}\pm1.14\times10^{-3}$	$2.73\times10^{-3}\pm5.80\times10^{-4}$	$4.12\times10^{-3}\pm2.34\times10^{-3}$
2-Oxoglutarate	$6.46\times10^{-5}\pm3.65\times10^{-5}$	$2.84\times10^{-5}\pm1.44\times10^{-5}$	$5.66\times10^{-5}\pm3.93\times10^{-5}$	$6.36\times10^{-5}\pm8.58\times10^{-6}$	$4.83\times10^{-5}\pm2.50\times10^{-5}$
Asparagine	$1.18\times10^{-3}\pm1.24\times10^{-3}$	$2.20\times10^{-3}\pm2.22\times10^{-3}$	$2.37\times10^{-3}\pm1.17\times10^{-3}$	$1.25\times10^{-3}\pm9.25\times10^{-4}$	$2.31\times10^{-3}\pm1.87\times10^{-3}$
N-Methylhydantoin	$4.16\times10^{-5}\pm1.28\times10^{-5}$	$2.08\times10^{-5}\pm1.20\times10^{-5}$	$4.28\times10^{-5}\pm1.90\times10^{-5}$	$2.83\times10^{-5}\pm1.44\times10^{-5}$	$2.91\times10^{-5}\pm1.72\times10^{-5}$
Trimethylamine	$1.04\times10^{-4}\pm4.15\times10^{-5}$	$2.19\times10^{-4}\pm4.32\times10^{-5}$	$1.34\times10^{-4}\pm5.48\times10^{-5}$	$1.55\times10^{-4}\pm4.69\times10^{-5}$	$2.08\times10^{-4}\pm6.06\times10^{-5}$
Sarcosine	$1.64\times10^{-4}\pm5.51\times10^{-5}$	$1.09\times10^{-4}\pm3.47\times10^{-5}$	$4.40\times10^{-4}\pm1.88\times10^{-4}$	$2.32\times10^{-4}\pm1.27\times10^{-4}$	$3.92\times10^{-4}\pm2.68\times10^{-4}$
Dimethylamine	$3.29\times10^{-5}\pm2.57\times10^{-5}$	$6.48\times10^{-5}\pm8.14\times10^{-5}$	$1.93\times10^{-5}\pm1.64\times10^{-5}$	$3.59\times10^{-5}\pm1.83\times10^{-5}$	$1.83\times10^{-5}\pm1.04\times10^{-5}$
Aspartate	$2.11\times10^{-3}\pm1.01\times10^{-3}$	$2.64\times10^{-3}\pm2.49\times10^{-3}$	$2.70\times10^{-3}\pm9.50\times10^{-4}$	$1.95\times10^{-3}\pm8.30\times10^{-4}$	$2.66\times10^{-3}\pm1.69\times10^{-3}$
2-Oxoisocaproate	$9.47\times10^{-5}\pm6.39\times10^{-5}$	$6.88\times10^{-5}\pm4.74\times10^{-5}$	$6.78\times10^{-5}\pm7.93\times10^{-5}$	$6.15\times10^{-5}\pm3.28\times10^{-5}$	$7.05\times10^{-5}\pm5.38\times10^{-5}$
Methylamine	$7.93\times10^{-5}\pm4.93\times10^{-5}$	$6.53\times10^{-5}\pm3.43\times10^{-5}$	$6.29\times10^{-5}\pm7.54\times10^{-5}$	$6.35\times10^{-5}\pm4.44\times10^{-5}$	$8.85\times10^{-5}\pm6.89\times10^{-5}$
Glutamine	$1.90\times10^{-3}\pm1.31\times10^{-3}$	$2.75\times10^{-3}\pm2.35\times10^{-3}$	$2.90\times10^{-3}\pm1.18\times10^{-3}$	$1.70\times10^{-3}\pm5.33\times10^{-4}$	$2.86\times10^{-3}\pm2.01\times10^{-3}$
Succinate	$1.27\times10^{-3}\pm8.49\times10^{-4}$	$2.60\times10^{-3}\pm2.46\times10^{-3}$	$5.09\times10^{-4}\pm3.59\times10^{-4}$	$3.80\times10^{-4}\pm1.03\times10^{-4}$	$6.39\times10^{-4}\pm2.47\times10^{-4}$
Pyruvate	$2.85\times10^{-4}\pm1.36\times10^{-4}$	$2.72\times10^{-4}\pm7.48\times10^{-5}$	$3.05\times10^{-4}\pm1.12\times10^{-4}$	$2.11\times10^{-4}\pm2.74\times10^{-5}$	$1.51\times10^{-4}\pm2.69\times10^{-5}$
Glutamate	$4.98\times10^{-3}\pm1.14\times10^{-3}$	$7.23\times10^{-3}\pm5.16\times10^{-3}$	$6.08\times10^{-3}\pm1.35\times10^{-3}$	$5.65\times10^{-3}\pm1.36\times10^{-3}$	$7.15\times10^{-3}\pm3.63\times10^{-3}$
5-Aminopentanoate	$5.63\times10^{-4}\pm2.53\times10^{-4}$	$8.37\times10^{-4}\pm6.03\times10^{-4}$	$2.99\times10^{-4}\pm3.01\times10^{-5}$	$5.06\times10^{-4}\pm2.07\times10^{-4}$	$3.65\times10^{-4}\pm2.56\times10^{-5}$
Methionine	$1.38\times10^{-3}\pm6.64\times10^{-4}$	$2.23\times10^{-3}\pm1.95\times10^{-3}$	$1.67\times10^{-3}\pm7.03\times10^{-4}$	$1.23\times10^{-3}\pm3.29\times10^{-4}$	$1.99\times10^{-3}\pm1.59\times10^{-3}$
Acetate	$1.61\times10^{-2}\pm1.04\times10^{-2}$	$1.54\times10^{-2}\pm1.14\times10^{-2}$	$8.35\times10^{-3}\pm9.64\times10^{-3}$	$1.05\times10^{-2}\pm7.93\times10^{-3}$	$1.18\times10^{-2}\pm7.24\times10^{-3}$
Thymine	$1.90\times10^{-4}\pm7.97\times10^{-5}$	$3.33\times10^{-4}\pm2.53\times10^{-4}$	$1.66\times10^{-4}\pm5.71\times10^{-5}$	$1.71\times10^{-4}\pm5.80\times10^{-5}$	$2.66\times10^{-4}\pm1.46\times10^{-4}$
Butyrate	$1.96\times10^{-3}\pm1.75\times10^{-3}$	$2.90\times10^{-3}\pm1.91\times10^{-3}$	$9.99\times10^{-4}\pm1.38\times10^{-3}$	$1.98\times10^{-3}\pm1.63\times10^{-3}$	$4.14\times10^{-3}\pm3.77\times10^{-3}$

Alanine	$5.65\times10^{-3}\pm7.98\times10^{-4}$	$6.54\times10^{-3}\pm3.70\times10^{-3}$	$5.64\times10^{-3}\pm1.09\times10^{-3}$	$4.68\times10^{-3}\pm8.32\times10^{-4}$	$6.67\times10^{-3}\pm3.36\times10^{-3}$
Acetoin	$7.77\times10^{-4}\pm8.62\times10^{-4}$	$1.75\times10^{-4}\pm1.19\times10^{-4}$	$4.72\times10^{-5}\pm7.10\times10^{-5}$	$1.63\times10^{-4}\pm2.39\times10^{-4}$	$9.57\times10^{-5}\pm9.34\times10^{-5}$
Lactate	$1.45\times10^{-2}\pm6.65\times10^{-3}$	$9.44\times10^{-3}\pm2.97\times10^{-3}$	$8.80\times10^{-3}\pm2.96\times10^{-3}$	$1.07\times10^{-2}\pm1.54\times10^{-3}$	$1.00\times10^{-2}\pm2.77\times10^{-3}$
Fucose	$2.23\times10^{-4}\pm6.11\times10^{-5}$	$2.68\times10^{-4}\pm2.43\times10^{-4}$	$1.16\times10^{-4}\pm7.71\times10^{-5}$	$1.68\times10^{-4}\pm1.13\times10^{-4}$	$1.06\times10^{-4}\pm4.32\times10^{-5}$
Ethanol	$1.04\times10^{-3}\pm5.39\times10^{-4}$	$1.68\times10^{-3}\pm1.47\times10^{-3}$	$5.04\times10^{-4}\pm2.44\times10^{-4}$	$1.85\times10^{-3}\pm1.64\times10^{-3}$	$8.36\times10^{-4}\pm4.02\times10^{-4}$
Propylene glycol	$1.96\times10^{-4}\pm1.46\times10^{-4}$	$1.52\times10^{-4}\pm1.35\times10^{-4}$	$7.72\times10^{-5}\pm9.18\times10^{-5}$	$1.01\times10^{-4}\pm3.59\times10^{-5}$	$8.27\times10^{-5}\pm6.65\times10^{-5}$
3-Methyl-2-oxovalerate	$1.01\times10^{-4}\pm4.26\times10^{-5}$	$5.25\times10^{-5}\pm3.18\times10^{-5}$	$4.71\times10^{-5}\pm4.02\times10^{-5}$	$4.39\times10^{-5}\pm2.24\times10^{-5}$	$6.19\times10^{-5}\pm3.77\times10^{-5}$
Propionate	$3.36\times10^{-3}\pm3.02\times10^{-3}$	$3.42\times10^{-3}\pm2.66\times10^{-3}$	$1.14\times10^{-3}\pm1.78\times10^{-3}$	$1.71\times10^{-3}\pm1.56\times10^{-3}$	$2.52\times10^{-3}\pm2.40\times10^{-3}$
Isoleucine	$1.72\times10^{-3}\pm7.56\times10^{-4}$	$2.59\times10^{-3}\pm2.13\times10^{-3}$	$2.42\times10^{-3}\pm9.13\times10^{-4}$	$1.81\times10^{-3}\pm5.58\times10^{-4}$	$2.61\times10^{-3}\pm1.73\times10^{-3}$
Valine	$2.09\times10^{-3}\pm8.88\times10^{-4}$	$3.39\times10^{-3}\pm2.90\times10^{-3}$	$2.94\times10^{-3}\pm1.19\times10^{-3}$	$2.12\times10^{-3}\pm5.33\times10^{-4}$	$3.47\times10^{-3}\pm2.33\times10^{-3}$
Leucine	$3.46\times10^{-3}\pm1.76\times10^{-3}$	$4.81\times10^{-3}\pm4.42\times10^{-3}$	$4.55\times10^{-3}\pm2.21\times10^{-3}$	$2.70\times10^{-3}\pm9.14\times10^{-4}$	$5.05\times10^{-3}\pm3.81\times10^{-3}$
Isovalerate	$3.33\times10^{-4}\pm2.96\times10^{-4}$	$1.70\times10^{-4}\pm1.50\times10^{-4}$	$1.28\times10^{-4}\pm1.70\times10^{-4}$	$2.43\times10^{-4}\pm1.84\times10^{-4}$	$1.04\times10^{-4}\pm5.45\times10^{-5}$
Valerate	$1.68\times10^{-4}\pm1.85\times10^{-4}$	$1.33\times10^{-4}\pm1.70\times10^{-4}$	$7.90\times10^{-5}\pm7.93\times10^{-5}$	$7.04\times10^{-5}\pm6.46\times10^{-5}$	$2.58\times10^{-4}\pm2.35\times10^{-4}$
Valproate	$3.01\times10^{-5}\pm2.73\times10^{-5}$	$1.75\times10^{-5}\pm1.49\times10^{-5}$	$1.20\times10^{-5}\pm2.11\times10^{-5}$	$1.65\times10^{-5}\pm1.31\times10^{-5}$	$1.77\times10^{-5}\pm1.64\times10^{-5}$
2-Hydroxyisovalerate	$3.42\times10^{-5}\pm3.39\times10^{-5}$	$7.53\times10^{-6}\pm6.36\times10^{-6}$	$6.25\times10^{-6}\pm1.10\times10^{-5}$	$6.58\times10^{-6}\pm5.79\times10^{-6}$	$7.49\times10^{-6}\pm3.86\times10^{-6}$
Cholate	$4.79\times10^{-4}\pm4.91\times10^{-4}$	$6.70\times10^{-5}\pm6.67\times10^{-5}$	$5.07\times10^{-5}\pm3.14\times10^{-5}$	$1.52\times10^{-4}\pm1.36\times10^{-4}$	$3.62\times10^{-5}\pm3.02\times10^{-5}$
